# Crystal structure and computational analysis of tetra­kis­(aceto­nitrile)­bis­(nona­fluoro-*tert*-butanolato)titanium(III) complex as a salt of the weakly coordinating [Al{OC(CF_3_)_3_}_4_]^−^ anion

**DOI:** 10.1107/S2056989025011314

**Published:** 2026-01-01

**Authors:** Przemysław Jan Malinowski, Kacper Koteras

**Affiliations:** a Centre of New Technologies University of Warsaw, Banacha 2c 02-097 Warsaw Poland; University of Massachusetts Dartmouth, USA

**Keywords:** crystal structure, titanium, weakly coordinating anion, aceto­nitrile, alkoxide

## Abstract

The title compound, [Ti(C_4_F_9_O)_2_(C_2_H_3_N)_4_][Al(C_4_F_9_O)_4_] or [Ti{OC(CF_3_)_3_}_2_(CH_3_CN)_4_][Al{OC(CF_3_)_3_}_4_], is the first structurally characterized complex of Ti^III^ containing four aceto­nitrile mol­ecules and perfluorinated alk­oxy­aluminate in its coordination sphere. The [Ti{OC(CF_3_)_3_}_2_(CH_3_CN)_4_]^+^ cation adopts a compressed octa­hedral geometry where aceto­nitrile occupies the equatorial positions with apically bound alkoxide anions.

## Abstract 2

The crystal structure of the first complex of titanium(III) with aceto­nitrile and perfluorinated *tert*-butoxide ligands crystallizes in space group *P*1 with the titanium(III) atoms coordinated octa­hedrally by four aceto­nitrile mol­ecules and two perfluorinated *tert*-butoxide anions.

## 1. Chemical context

The development of so-called weakly coordinating anions (WCAs) – species designed to inter­act with cations as weakly as possible, and often exhibiting exceptional chemical robustness – has enabled the synthesis and detailed characterization of many novel cationic species with unique structures and/or reactivity. This spans from a series of novel complexes containing ligands with poor coordinative properties such as dihalogen mol­ecules (Malinowski *et al.*, 2016 ▸), di­nitro­gen (Willrett *et al.*, 2024 ▸) or even pantane (Sellin *et al.*, 2025 ▸) through very reactive species like *M*(CO)_6_^+^ (*M* = Cr, Mo, W; Bohnenberger *et al.*, 2020 ▸) or a series of complexes containing neutral metal carbonyls (Sellin *et al.*, 2023 ▸; Wang *et al.*, 2017 ▸). Inter­estingly, there are only a few simple salts of divalent metal cations with the most advanced WCAs, such as halogenated carborates (*e.g.* Xu *et al.*, 2025 ▸) and perfluorinated alk­oxy­aluminates (*e.g.* Dabringhaus *et al.*, 2020 ▸) in which the cations are not coordinated by any auxiliary ligands. The reason for this is that in a weakly Lewis-basic environment provided by coordination exclusively by WCAs, the reactivity of even simple dications can be sufficient to trigger a reaction with very robust species, including the WCA itself (Schorpp & Krossing, 2020 ▸; Jadwiszczak & Malinowski, 2023 ▸). For this reason, ligands like aceto­nitrile (ACN) have been used extensively to ‘tame’ the central dication (Rach & Kühn, 2009 ▸). This approach has been successful, and as a consequence, many structures of metal–aceto­nitrile complexes have been synthesized. However, there are only a few such structures reported for early transition metals like vanadium in low (*i.e.* +2) oxidation state (*e.g.* Chandrasekhar & Bird, 1985 ▸), while for Ti^II^ there are none.

In the current contribution, we present a crystal structure of a compound that formed during an attempt to extend the Ti^II^ coordination chemistry by obtaining a Ti^II^–aceto­nitrile complex stabilized by a very robust perfluorinated alk­oxy­aluminate: [Al{OC(CF_3_)_3_}_4_]^−^. However, the crystal obtained from the post reaction mixture is actually [Ti{OC(CF_3_)_3_}_2_(ACN)_4_][Al{OC(CF_3_)_3_}_4_] (**1**), a complex of Ti^III^ and perfluorinated alk­oxy­aluminate groups bound to the metal. This suggests that Ti^II^ is incompatible with the anion and causes its decomposition. Nonetheless, this is a rare example of a structure of Ti^III^ ligated with several aceto­nitrile ligands, and one of a very few in which perfluorinated alkoxide is present in the coordination sphere of titanium.
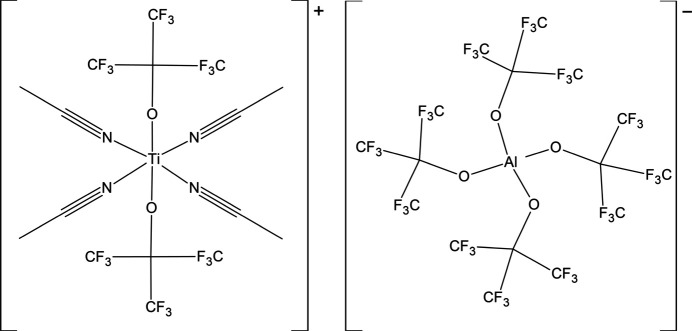


## 2. Structural commentary

**Figure 1:**
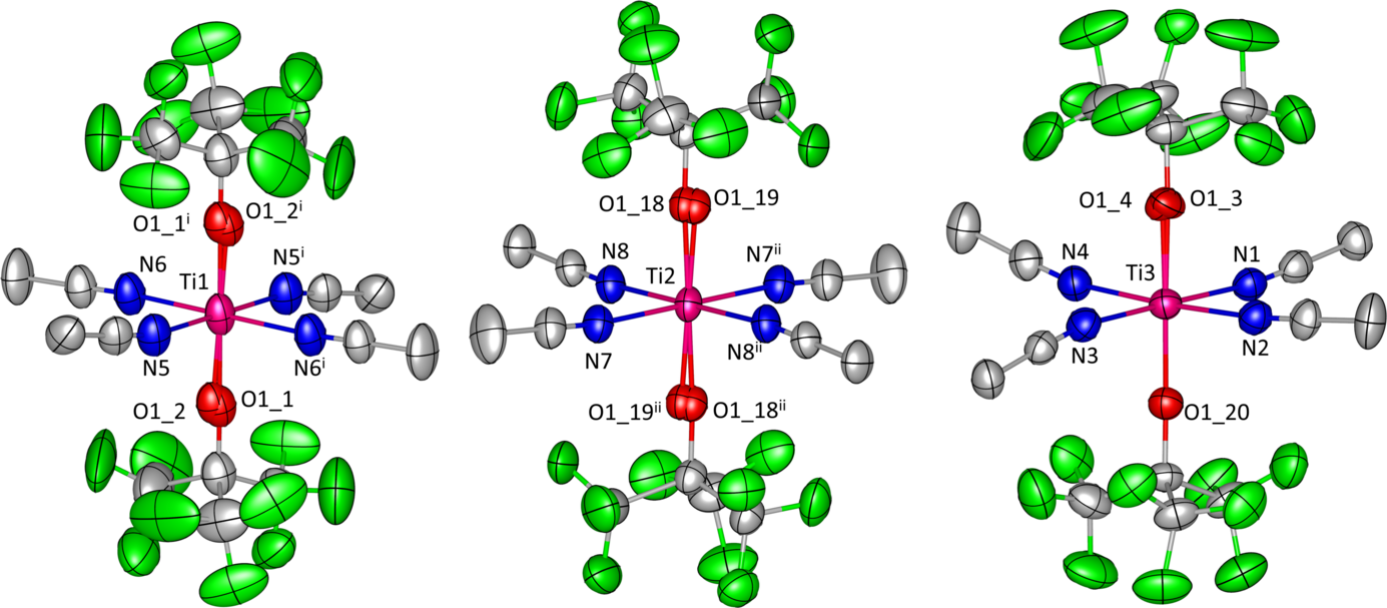
A view showing the labelling of the unique [Ti{OC(CF_3_)_3_}_2_(ACN)_4_]^+^ cations present in the crystal structure of **1**. Only oxygen atoms from disordered OC(CF_3_)_3_ groups with lower occupancy are shown for clarity. Symmetry codes: (i) −*x*, 1 − *y*, −*z*; (ii) −*x*, −*y*, −*z*.

The title compound crystallizes in the triclinic *P*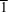 space group with a unit cell containing four structural units of [Til{OC(CF_3_)_3_}_2_(ACN)_4_][Al{OC(CF_3_)_3_}_4_]. The asymmetric unit contains three cations, two of which lie on inversion centres (Ti1 and Ti2) and they all have a very similar geometry (Fig. 1 ▸ and Table 1 ▸). The coordination sphere of titanium is a compressed octa­hedron with equatorial positions occupied by aceto­nitrile mol­ecules at 2.163 (4)–2.184 (4) Å for the Ti—N separations (Table 1 ▸) and alkoxide anions residing on apical positions with Ti—O distances of 1.818 (10)–1.897 (8) Å. Ligands are bound almost in a linear fashion, *i.e.* angles Ti—N—C (for aceto­nitriles) and Ti—O—C (for alkoxides) are all close to 180° with the exception of one aceto­nitrile mol­ecule (bound to Ti3) for which it is 166.4 (4)°. The structure exhibits a very high degree of disorder in the –OC(CF_3_)_3_ groups – out of fourteen unique ones, only four are not disordered. It is not uncommon in structures containing perfluorinated *tert*-butyl groups (Krossing & Reisinger, 2006 ▸). The refined occupancy factors vary from 0.57 to 0.93.

## 3. Supra­molecular features

**Figure 2:**
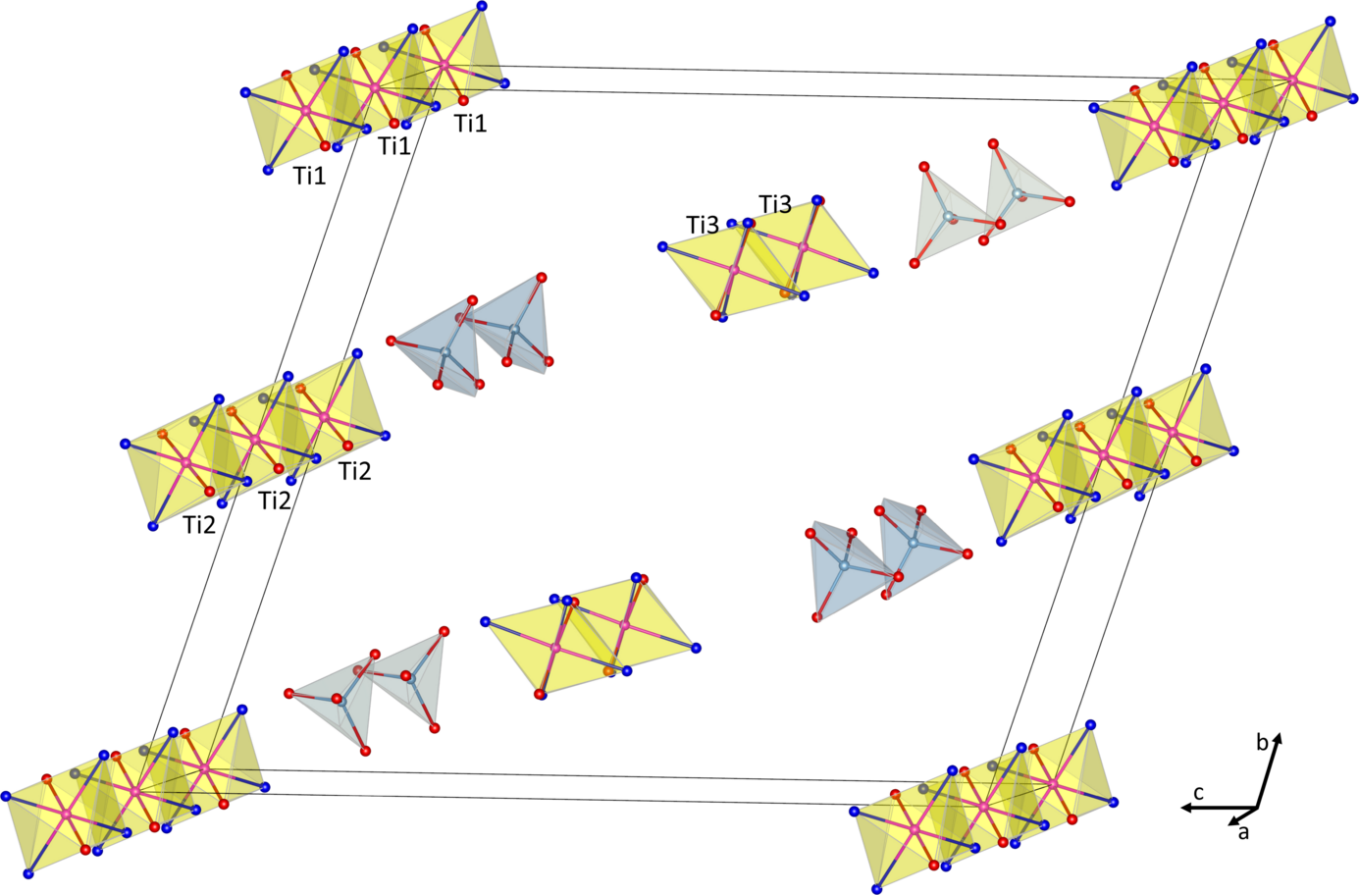
The arrangement of cations and anions in the crystal of **1**. Only TiO_4_N_2_ (yellow) and AlO_4_ (grey) polyhedra are shown for clarity.

The anions and cations in the structure do not form any strong and direct bonds, which is typical for salts of WCAs. The structure can be viewed as an array of cations residing on the (001) (Ti1, and Ti2) and (002) (Ti3) crystal planes and anions being placed between cations (Fig. 2 ▸).

**Figure 3:**
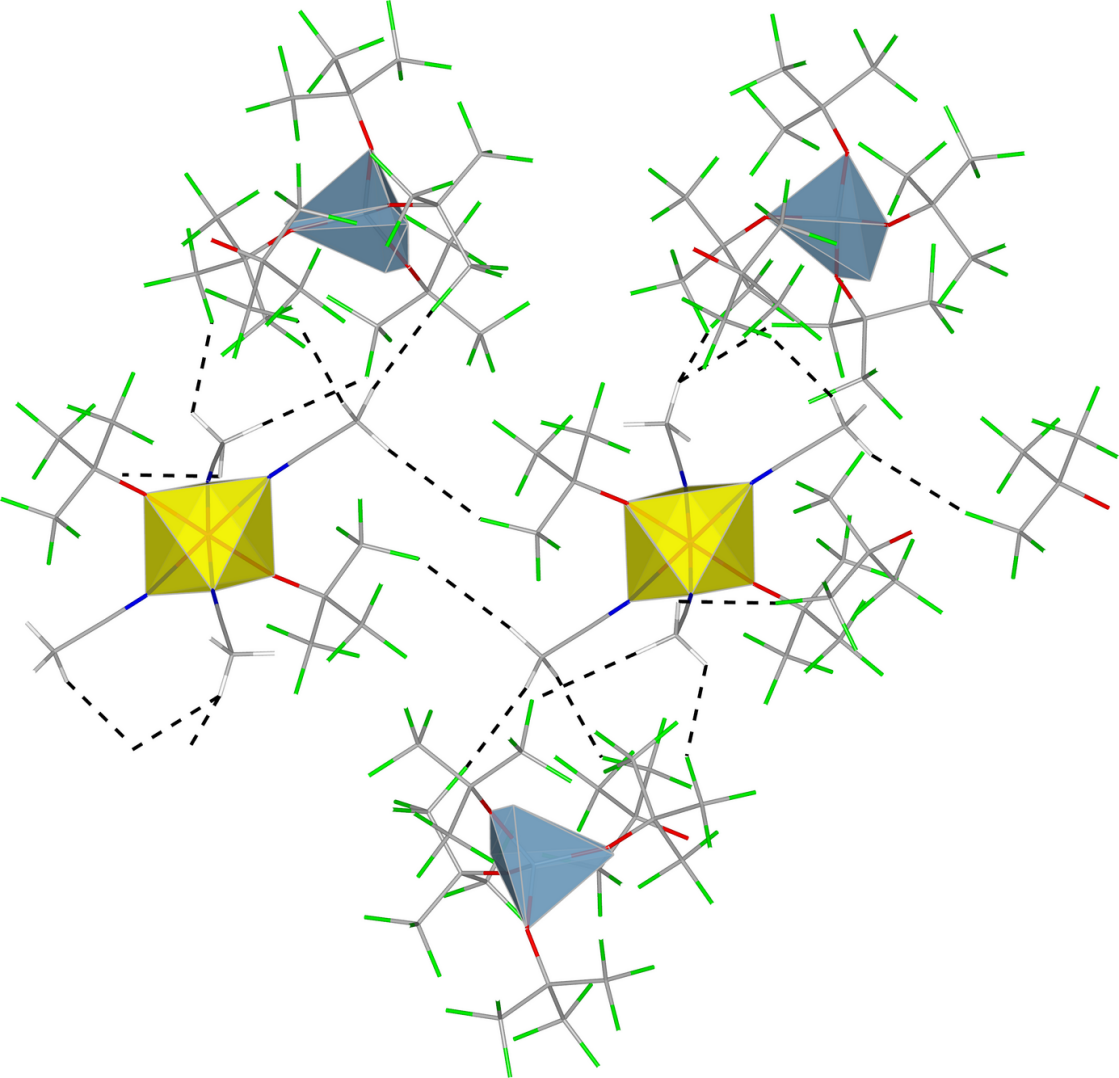
View of a fragment of the crystal structure of **1** showing H⋯F contacts (black dashed lines) shorter than the sum of their van der Waals radii from aceto­nitrile ligands belonging to cations containing Ti3. For clarity, only OC(CF_3_)_3_ groups with higher occupancies are shown.

The distances between hydrogen atoms from ACN mol­ecules and fluorine atoms belonging to anions suggest that there is a weak hydrogen bond between anions and cations (see Fig. 3 ▸). Of course, CF_3_ group is a very poor hydrogen-bond acceptor and its presence in such systems is a matter of long debate (Wang *et al.*, 2024 ▸). However, in this particular compound, it is not unlikely to see such inter­actions since aceto­nitrile, although not a good hydrogen-bond donor, has some positive charge on the CH_3_ group resulting from the presence of the –CN group and, additionally, the coordination to cationic species. Thus, in the absence of other negatively charged sites, the CF_3_ group is the only one with which it could inter­act. Therefore, this and other salts of [Al{OC(CF_3_)_3_}_4_]^−^ anions can be regarded as good model systems to investigate weak hydrogen bonds involving tri­fluoro­methyl groups.

## 4. Hirshfeld surface analysis and DFT computations

More details about contacts between ions in the crystal of **1** can be obtained from the analysis of Hirschfeld surfaces (HS), which were calculated using *CrystalExplorer 21.5* (Spackman *et al.*, 2021 ▸) and were mapped with the function *d*_norm_ using high resolution settings. The colour scheme for HS is typical with red regions marking separation of adjacent atoms below the sum of their van der Waals radii, blue where it is higher, and white where it is approximately equal to the sum.

**Figure 4:**
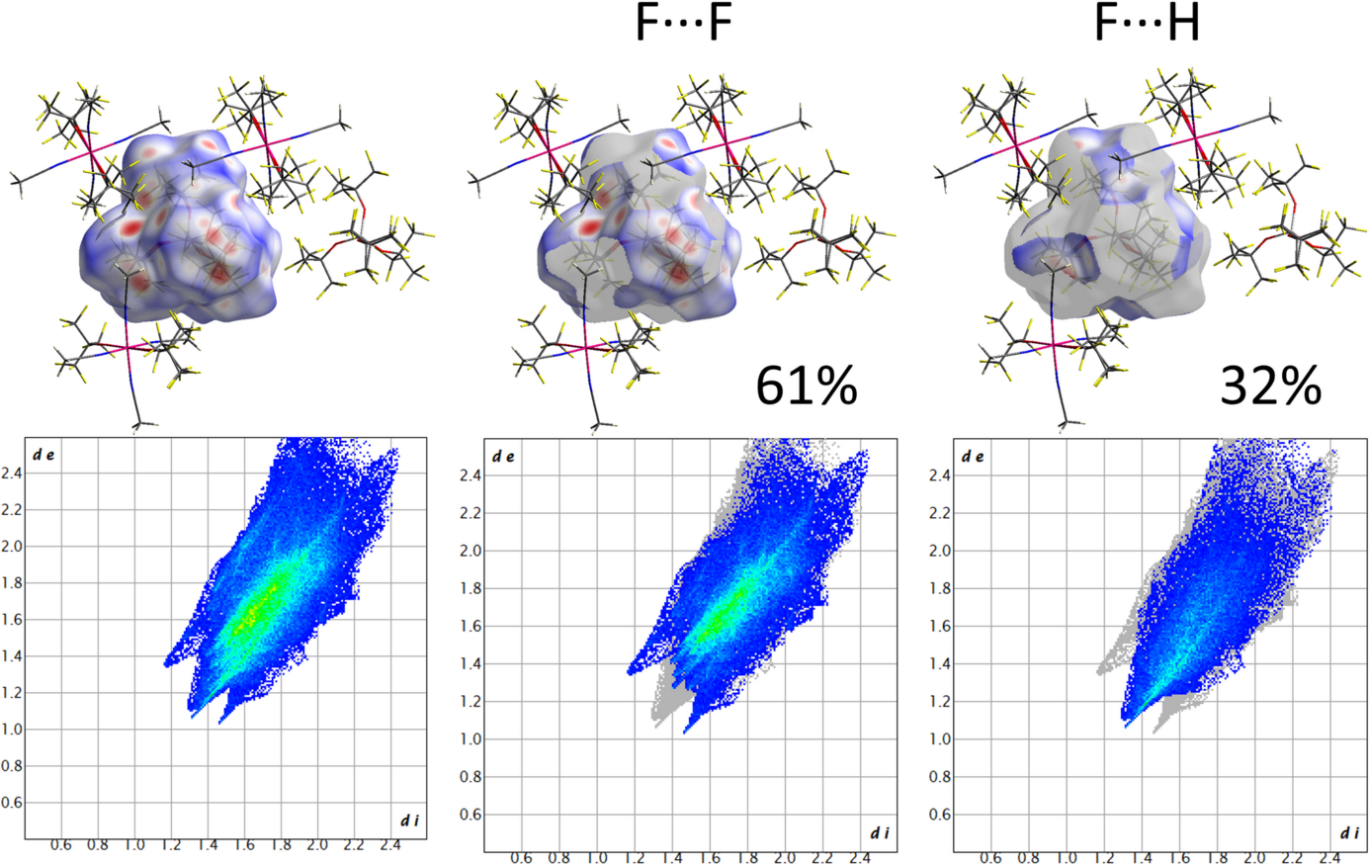
Top: Hirshfeld surface for the anion containing the Al2 atom and delineated into F⋯F and F⋯H contacts (the latter atom is outside the surface). Bottom: fingerprint plots corresponding to these surfaces.

**Figure 5:**
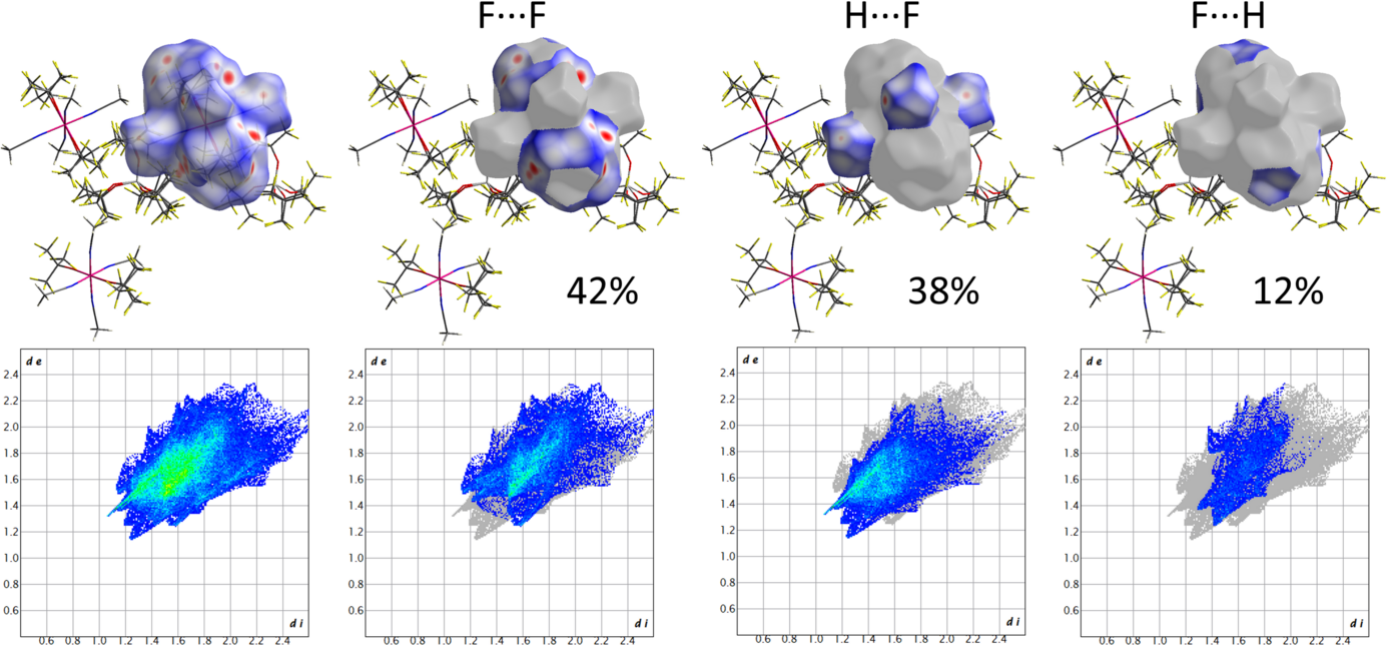
Top: Hirshfeld surface for the anion containing the Ti2 atom and delineated into F⋯F, H⋯F and F⋯H contacts (latter atom is outside the surface). Bottom: fingerprint plots corresponding to these surfaces.

Both anions form contacts exclusively through fluorine atoms and they have a similar contribution of F⋯F (*ca.* 61%) and F⋯H (*ca.* 33%) contacts (Fig. 4 ▸). Also cations have a similar profile of inter­actions with neighbouring species (Fig. 5 ▸). Between 40% and 45% of the Hirshfeld surface corresponds to F⋯F distances, while H⋯F makes up 38–39% and F⋯H an additional 7–14% (the former atom is inside, while the latter is outside the surface). Red areas on these surfaces indicate that there are direct inter­actions between CH_3_ and CF_3_ groups of neighbouring anions and cations.

An inter­esting feature of **1** is that CF_3_ groups from different entities are closer than the sum of van der Waals radii of two fluorine atoms and it deserves a short comment. It has to be noted that since **1** is an ionic compound, the most significant inter­actions between anions and cations are electrostatic forces. Therefore, short contacts between F atoms are not a result of the inter­action between two CF_3_ groups, but rather are a consequence of strong inter­actions between ions to which they belong. Analysis of other salts composed of cationic complexes containing perfluorinated alkoxides in their coordination spheres and fluorinated anions (see *Database survey*) also reveals the presence of similarly ‘short’ F⋯F contacts.

The structure of the cation is very well reproduced in a DFT optimization of the isolated cation. Computations performed using* Orca 6.0* (Neese, 2025 ▸) yield the structure with Ti—O bond lengths of 1.855 Å and Ti—N of 2.194 Å. It has to be noted that the method used: ωB97X-V functional (Mardirossian & Head-Gordon, 2014 ▸) with def2-TZVP basis set (Weigend & Ahlrichs, 2005 ▸), def2/j auxiliary basis set (Weigend, 2006 ▸) and D4 dispersion correction (Caldeweyher *et al.*, 2020 ▸) performs very well in terms of accuracy of geometry optimizations. Additionally, we wanted to verify what is the effect of perfluorination of *tert*-butyl groups on the geometry of the complex. It was performed by the optimization of a structure of the related non-fluorinated complex: [Ti{OC(CH_3_)_3_}_2_(ACN)_4_]^+^. It turns out that in such complex, the Ti—O distances are shorter (1.821 Å) while Ti—N are longer (2.238 Å) than in the fluorinated counterpart, meaning that without fluorine atoms the octa­hedron is more compressed. This is not unexpected, since fluorination reduces charge and basicity of oxygen atoms in alcoholate groups, which typically results in weakening of bonding with metal cations and longer metal–O bonds. Computational details can be found in ESI.

## 5. Database survey

A search of the Cambridge Structural Database (CSD, Version 6.00, Aug. 2025; Groom *et al.*, 2016 ▸) with the use of the ConQuest program (version 2025.2.0; Bruno *et al.*, 2002 ▸) did not reveal any compound that would have a cation with a similar structure to that in **1**. Generally, reports on metal complexes bearing four ACN mol­ecules and an additional two oxygen atoms in the coordination sphere of any transition metal are not abundant and gave only 19 hits, most of which are hydrates or triflates. The structures of complexes in which titanium (in any oxidation state) binds to at least four aceto­nitrile ligands without restricting other species ligating the metal, are also scarce, with only three structures of such complexes (all containing Ti^3+^) being reported in the CSD: [TiI_2_(ACN)_4_]^+^ [refcode KADVIU (Troyanov & Mazo, 1988 ▸); KADVIU01 (Leigh *et al.*, 2002 ▸)], [TiCp(ACN)_5_]^+^ (LEHROF; Willey *et al.*, 1994 ▸), [TiCl_2_(ACN)_4_]^+^ (YEWHOX; Kharisov *et al.*, 1994 ▸).

In addition, ionic metal complexes in which the cations are discrete (*i.e.* do not form one entity with an anion) and contain an –OC(CF_3_)_3_ group are rare and include only 14 species [APIYAP (Paul *et al.*, 2021 ▸); KOBVAC, KOBVIK, KOBVOQ (Petit *et al.*, 2023 ▸); LUMPER, LUMPOB (Bohnenberger *et al.*, 2020 ▸); MALTIG, MALTON, MALTUS, MALVAA (Benedikter *et al.*, 2020 ▸); SICMIB, SICMOH (Reisinger *et al.*, 2007 ▸); SOQMAQ (Probst *et al.*, 2024 ▸)].

## 6. Synthesis and crystallization

An equimolar mixture of of TiCl_2_ (50 mg, 0.42 mmol; Sigma Aldrich) and Li[Al{OC(CF_3_)_3_}_4_] (410 mg, 0.42 mmol) was added to 2 ml of dry and degassed aceto­nitrile. The mixture turned blue immediately and was left on stirring overnight. After that time, the solution was filtered through a glass frit (P4) and slowly evaporated to yield crystals suitable for X-ray diffraction studies. Li[Al{OC(CF_3_)_3_}_4_] was synthesized according to the literature procedure (Malinowski *et al.*, 2020 ▸). Although a typical approach to form transition metal–aceto­nitrile complexes utilizes silver(I) salt as the halide scavenger, in this case, the strongly reductive character of Ti^II^ makes it incompatible with silver(I) compounds. Therefore, the lithium salt was chosen.

Because the compounds used in the study are highly moisture and oxygen-sensitive, all manipulations and reactions were conducted in an argon-filled glovebox with O_2_ and H_2_O levels not exceeding 1 ppm. Crystals were covered with Krytox^®^ 1531 perfluorinated oil to protect them from the atmosphere during handling under microscope.

## 7. Refinement

Crystal data, data collection and structure refinement details are summarized in Table 2 ▸. Because of the very high disorder of the –OC(CF_3_)_3_ groups, the *DSR* program (Kratzert & Krossing, 2015 ▸) was used to facilitate its proper modelling. The program automatically generates a set of additional restraints and constraints on their geometry to keep it reasonable and these include the equality of C—C and C—F bond lengths within these groups, as well as C—C—C and C—C—F angles. Additionally, additional constraints on ADP parameters (EADP command) were applied on atoms from two disordered groups, which are located very close to each other.

## Supplementary Material

Crystal structure: contains datablock(s) I. DOI: 10.1107/S2056989025011314/yy2021sup1.cif

Structure factors: contains datablock(s) I. DOI: 10.1107/S2056989025011314/yy2021Isup2.hkl

Supporting information file. DOI: 10.1107/S2056989025011314/yy2021Isup3.cdx

Supporting information file. DOI: 10.1107/S2056989025011314/yy2021sup4.docx

CCDC reference: 2515927

Additional supporting information:  crystallographic information; 3D view; checkCIF report

## Figures and Tables

**Table 1:** Selected bond lengths (Å) within [Ti{OC(CF_3_)_3_}_2_(ACN)_4_]^+^ cations

Ti1—O1_2	1.818 (10)	Ti2—O1_18	1.867 (13)	Ti3—O1_3	1.863 (10)
Ti1—N5	2.163 (4)	Ti2—N7	2.170 (3)	Ti3—O1_4	1.854 (8)
Ti1—N6	2.170 (4)	Ti2—N8	2.173 (3)	Ti3—O1_20	1.850 (3)
				Ti3—N3	2.177 (4)
				Ti3—N4	2.182 (3)
				Ti3—N1	2.184 (4)
				Ti3—N2	2.177 (3)

**Table 2:** Experimental details

Crystal data	
Chemical formula	[Ti(C_4_F_9_O)_2_(C_2_H_3_N)_4_][Al(C_4_F_9_O)_4_]
*M*_r_	1649.34
Crystal system, space group	Triclinic, *P* 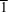
Temperature (K)	100
*a*, *b*, *c* (Å)	10.5592 (2), 21.6580 (3), 25.3018 (3)
α, β, γ (°)	106.706 (1), 91.638 (2), 99.588 (2)
*V* (Å^3^)	5446.71 (15)
*Z*	4
Radiation type	Cu *K*α
μ (mm^−1^)	3.70
Crystal size (mm)	0.24 × 0.21 × 0.13

Data collection	
Diffractometer	SuperNova, Single source at offset/far, Atlas
Absorption correction	Multi-scan (*CrysAlis PRO*; Rigaku OD, 2022 ▸)
*T*_min_, *T*_max_	0.490, 1.000
No. of measured, independent and observed [*I* > 2σ(*I*)] reflections	142670, 22208, 15553
*R*_int_	0.094
(sin θ/λ)_max_ (Å^−1^)	0.631

Refinement	
*R*[*F*^2^ > 2σ(*F*^2^)], *wR*(*F*^2^), *S*	0.072, 0.220, 1.05
No. of reflections	22208
No. of parameters	2534
No. of restraints	59905
H-atom treatment	H-atom parameters constrained
Δρ_max_, Δρ_min_ (e Å^−3^)	0.94, −0.76

Computer programs: *CrysAlis PRO* (Rigaku OD, 2022 ▸), *SHELXT2018/2* (Sheldrick, 2015*a* ▸), *SHELXL2025/1* (Sheldrick, 2015*b* ▸), *ShelXle* (Hübschle *et al.*, 2011 ▸) and *VESTA* (Momma & Izumi, 2011 ▸).

## supplementary crystallographic information

### Tetrakis(acetonitrile)bis(nonafluoro-tert-butanolato)titanium(III) tetrakis(nonafluoro-tert-butanolato)aluminate Crystal data

**Table:**

[Ti(C_4_F_9_O)_2_(C_2_H_3_N)_4_][Al(C_4_F_9_O)_4_]	*Z* = 4
*M**_r_* = 1649.34	*F*(000) = 3204
Triclinic, *P*1	*D*_x_ = 2.011 Mg m^−^^3^
*a* = 10.5592 (2) Å	Cu *K*α radiation, λ = 1.54184 Å
*b* = 21.6580 (3) Å	Cell parameters from 41497 reflections
*c* = 25.3018 (3) Å	θ = 2.2–75.6°
α = 106.706 (1)°	µ = 3.70 mm^−^^1^
β = 91.638 (2)°	*T* = 100 K
γ = 99.588 (2)°	Block, blue
*V* = 5446.71 (15) Å^3^	0.24 × 0.21 × 0.13 mm

### Tetrakis(acetonitrile)bis(nonafluoro-tert-butanolato)titanium(III) tetrakis(nonafluoro-tert-butanolato)aluminate Data collection

**Table:**

SuperNova, Single source at offset/far, Atlas diffractometer	22208 independent reflections
Radiation source: micro-focus sealed X-ray tube, SuperNova (Cu) X-ray Source	15553 reflections with *I* > 2σ(*I*)
Mirror monochromator	*R*_int_ = 0.094
ω scans	θ_max_ = 76.7°, θ_min_ = 2.2°
Absorption correction: multi-scan (CrysAlisPro; Rigaku OD, 2022)	*h* = −13→13
*T*_min_ = 0.490, *T*_max_ = 1.000	*k* = −27→26
142670 measured reflections	*l* = −31→31

### Tetrakis(acetonitrile)bis(nonafluoro-tert-butanolato)titanium(III) tetrakis(nonafluoro-tert-butanolato)aluminate Refinement

**Table:**

Refinement on *F*^2^	59905 restraints
Least-squares matrix: full	Hydrogen site location: inferred from neighbouring sites
*R*[*F*^2^ > 2σ(*F*^2^)] = 0.072	H-atom parameters constrained
*wR*(*F*^2^) = 0.220	*w* = 1/[σ^2^(*F*_o_^2^) + (0.1138*P*)^2^ + 5.6958*P*] where *P* = (*F*_o_^2^ + 2*F*_c_^2^)/3
*S* = 1.05	(Δ/σ)_max_ < 0.001
22208 reflections	Δρ_max_ = 0.94 e Å^−^^3^
2534 parameters	Δρ_min_ = −0.76 e Å^−^^3^

### Tetrakis(acetonitrile)bis(nonafluoro-tert-butanolato)titanium(III) tetrakis(nonafluoro-tert-butanolato)aluminate Special details

**Table:**

Geometry. All esds (except the esd in the dihedral angle between two l.s. planes) are estimated using the full covariance matrix. The cell esds are taken into account individually in the estimation of esds in distances, angles and torsion angles; correlations between esds in cell parameters are only used when they are defined by crystal symmetry. An approximate (isotropic) treatment of cell esds is used for estimating esds involving l.s. planes.
Refinement. Structure solution was performed using dual-space method (SHELXT-2018/2) and refined by full-matrix least squares on *F*^2^ (SHELXL-2025/1) using ShelXle as graphical interface (Hübschle *et al.*, 2011).

### Tetrakis(acetonitrile)bis(nonafluoro-tert-butanolato)titanium(III) tetrakis(nonafluoro-tert-butanolato)aluminate Fractional atomic coordinates and isotropic or equivalent isotropic displacement parameters (Å^2^)

**Table:**

	*x*	*y*	*z*	*U*_iso_*/*U*_eq_	Occ. (<1)
Ti1	0.000000	0.000000	0.000000	0.0510 (3)	
Al1	0.37160 (11)	0.84610 (5)	0.25070 (4)	0.0349 (2)	
N1	0.1506 (3)	0.32011 (16)	0.48778 (13)	0.0407 (7)	
C1	0.2269 (4)	0.3637 (2)	0.48840 (16)	0.0408 (9)	
Ti2	0.000000	0.500000	0.000000	0.0342 (2)	
Al2	0.33064 (11)	0.34855 (5)	0.23600 (4)	0.0329 (2)	
N2	0.0342 (3)	0.28392 (16)	0.58333 (14)	0.0416 (7)	
C2	0.3220 (4)	0.4211 (2)	0.48899 (19)	0.0493 (10)	
H2A	0.324657	0.425509	0.451549	0.074*	
H2B	0.407063	0.415876	0.501711	0.074*	
H2C	0.298339	0.460385	0.514110	0.074*	
C7	−0.1193 (4)	0.2038 (2)	0.36251 (18)	0.0451 (9)	
N4	−0.0699 (3)	0.21470 (16)	0.40553 (14)	0.0432 (8)	
C4	0.0990 (5)	0.3414 (3)	0.68741 (19)	0.0652 (14)	
H4A	0.118965	0.388922	0.693550	0.098*	
H4B	0.175116	0.326519	0.698783	0.098*	
H4C	0.027758	0.331088	0.709269	0.098*	
Ti3	−0.01351 (7)	0.24735 (3)	0.49423 (3)	0.03719 (17)	
N3	−0.1751 (3)	0.17524 (17)	0.50316 (14)	0.0424 (7)	
C3	0.0621 (4)	0.3086 (2)	0.62923 (18)	0.0453 (9)	
N5	−0.1492 (4)	−0.02926 (18)	−0.06732 (17)	0.0571 (10)	
C5	−0.2451 (4)	0.1303 (2)	0.50599 (16)	0.0405 (9)	
N6	0.0656 (4)	0.08682 (18)	−0.02533 (16)	0.0558 (10)	
C6	−0.3303 (5)	0.0723 (2)	0.50985 (18)	0.0491 (10)	
H6A	−0.419590	0.079159	0.508938	0.074*	
H6B	−0.307952	0.063712	0.544626	0.074*	
H6C	−0.320897	0.034623	0.478581	0.074*	
N7	−0.0593 (3)	0.40761 (16)	0.01765 (13)	0.0404 (7)	
N8	0.1491 (3)	0.52990 (15)	0.06799 (13)	0.0397 (7)	
C8	−0.1862 (5)	0.1918 (3)	0.30842 (19)	0.0612 (13)	
H8A	−0.161934	0.230178	0.295211	0.092*	
H8B	−0.279491	0.183977	0.311408	0.092*	
H8C	−0.162277	0.153340	0.282240	0.092*	
C9	−0.2335 (5)	−0.0355 (2)	−0.09886 (19)	0.0535 (11)	
C10	−0.3395 (6)	−0.0406 (3)	−0.1383 (2)	0.0654 (14)	
H10A	−0.305613	−0.034495	−0.172450	0.098*	
H10B	−0.390436	−0.006760	−0.122618	0.098*	
H10C	−0.394125	−0.084004	−0.146485	0.098*	
C11	0.0976 (5)	0.1293 (2)	−0.04217 (19)	0.0538 (11)	
C12	0.1371 (6)	0.1843 (3)	−0.0634 (3)	0.0795 (18)	
H12A	0.173087	0.169080	−0.099152	0.119*	
H12B	0.202700	0.216996	−0.037190	0.119*	
H12C	0.062401	0.204180	−0.068271	0.119*	
C13	−0.0981 (4)	0.3615 (2)	0.02924 (17)	0.0457 (10)	
C14	−0.1482 (7)	0.3017 (3)	0.0434 (2)	0.0744 (17)	
H14A	−0.233028	0.281905	0.023566	0.112*	
H14B	−0.156151	0.312412	0.083364	0.112*	
H14C	−0.089018	0.270659	0.032728	0.112*	
C15	0.2332 (4)	0.54247 (18)	0.10061 (16)	0.0391 (8)	
C16	0.3420 (4)	0.5584 (2)	0.14207 (18)	0.0494 (10)	
H16A	0.393392	0.523589	0.132975	0.074*	
H16B	0.309886	0.562253	0.178629	0.074*	
H16C	0.395631	0.600082	0.142439	0.074*	
O1_1	−0.1157 (19)	0.0456 (11)	0.0450 (8)	0.0555 (16)	0.570 (8)
C1_1	−0.1989 (9)	0.0788 (4)	0.0782 (4)	0.0587 (13)	0.570 (8)
C2_1	−0.1242 (11)	0.1457 (6)	0.1165 (4)	0.088 (3)	0.570 (8)
F1_1	−0.0488 (11)	0.1344 (7)	0.1532 (4)	0.163 (6)	0.570 (8)
F2_1	−0.2004 (9)	0.1855 (5)	0.1426 (5)	0.106 (3)	0.570 (8)
F3_1	−0.0551 (8)	0.1769 (4)	0.0861 (5)	0.119 (3)	0.570 (8)
C3_1	−0.3059 (9)	0.0925 (4)	0.0427 (4)	0.067 (2)	0.570 (8)
F4_1	−0.3572 (8)	0.0384 (5)	0.0030 (4)	0.106 (4)	0.570 (8)
F5_1	−0.2606 (10)	0.1385 (5)	0.0200 (4)	0.126 (3)	0.570 (8)
F6_1	−0.4004 (9)	0.1128 (4)	0.0721 (3)	0.084 (2)	0.570 (8)
C4_1	−0.2634 (12)	0.0361 (6)	0.1125 (5)	0.104 (4)	0.570 (8)
F7_1	−0.3581 (13)	−0.0084 (5)	0.0822 (5)	0.168 (5)	0.570 (8)
F8_1	−0.3310 (9)	0.0643 (5)	0.1534 (4)	0.108 (3)	0.570 (8)
F9_1	−0.1803 (15)	0.0102 (8)	0.1339 (6)	0.167 (6)	0.570 (8)
O1_2	−0.107 (3)	0.0416 (15)	0.0472 (11)	0.0555 (16)	0.430 (8)
C1_2	−0.1902 (11)	0.0693 (6)	0.0800 (5)	0.0587 (13)	0.430 (8)
C2_2	−0.1933 (15)	0.0444 (6)	0.1317 (5)	0.090 (4)	0.430 (8)
F1_2	−0.2358 (16)	−0.0188 (5)	0.1184 (6)	0.106 (5)	0.430 (8)
F2_2	−0.255 (2)	0.0816 (8)	0.1684 (6)	0.173 (7)	0.430 (8)
F3_2	−0.0755 (16)	0.0587 (9)	0.1562 (6)	0.181 (7)	0.430 (8)
C3_2	−0.1405 (15)	0.1448 (6)	0.1013 (7)	0.106 (5)	0.430 (8)
F4_2	−0.142 (2)	0.1661 (7)	0.0576 (8)	0.194 (8)	0.430 (8)
F5_2	−0.0219 (12)	0.1555 (5)	0.1251 (7)	0.124 (5)	0.430 (8)
F6_2	−0.214 (2)	0.1704 (11)	0.1397 (10)	0.219 (11)	0.430 (8)
C4_2	−0.3247 (13)	0.0523 (8)	0.0489 (5)	0.122 (5)	0.430 (8)
F7_2	−0.3174 (16)	0.0698 (10)	0.0028 (5)	0.136 (6)	0.430 (8)
F8_2	−0.4042 (14)	0.0856 (12)	0.0800 (8)	0.256 (12)	0.430 (8)
F9_2	−0.3612 (15)	−0.0129 (8)	0.0361 (7)	0.206 (8)	0.430 (8)
O1_3	−0.114 (2)	0.3119 (10)	0.5012 (12)	0.046 (5)	0.422 (8)
C1_3	−0.1719 (12)	0.3623 (6)	0.4988 (5)	0.0452 (9)	0.422 (8)
C2_3	−0.1081 (11)	0.3981 (6)	0.4588 (5)	0.047 (3)	0.422 (8)
F1_3	0.0065 (7)	0.4325 (4)	0.4803 (5)	0.088 (3)	0.422 (8)
F2_3	−0.178 (2)	0.4387 (10)	0.4466 (12)	0.066 (6)	0.422 (8)
F3_3	−0.0882 (14)	0.3567 (4)	0.4116 (4)	0.087 (4)	0.422 (8)
C3_3	−0.3154 (13)	0.3370 (7)	0.4792 (6)	0.089 (4)	0.422 (8)
F4_3	−0.3657 (12)	0.2960 (9)	0.5063 (9)	0.134 (7)	0.422 (8)
F5_3	−0.3304 (9)	0.3075 (5)	0.4253 (5)	0.113 (4)	0.422 (8)
F6_3	−0.381 (3)	0.3868 (12)	0.4884 (12)	0.117 (8)	0.422 (8)
C4_3	−0.1539 (15)	0.4122 (7)	0.5576 (5)	0.089 (4)	0.422 (8)
F7_3	−0.229 (2)	0.3877 (8)	0.5900 (6)	0.153 (7)	0.422 (8)
F8_3	−0.177 (2)	0.4708 (8)	0.5588 (9)	0.088 (5)	0.422 (8)
F9_3	−0.0335 (15)	0.4208 (5)	0.5798 (5)	0.124 (5)	0.422 (8)
O1_4	−0.1194 (14)	0.3085 (7)	0.4985 (8)	0.039 (3)	0.578 (8)
C1_4	−0.1805 (9)	0.3588 (5)	0.4987 (4)	0.0452 (9)	0.578 (8)
C2_4	−0.1334 (10)	0.4175 (5)	0.5523 (4)	0.063 (3)	0.578 (8)
F1_4	−0.1397 (9)	0.3952 (4)	0.5960 (3)	0.075 (2)	0.578 (8)
F2_4	−0.2140 (17)	0.4602 (7)	0.5584 (8)	0.112 (5)	0.578 (8)
F3_4	−0.0136 (7)	0.4451 (3)	0.5500 (3)	0.088 (2)	0.578 (8)
C3_4	−0.1476 (9)	0.3827 (5)	0.4478 (4)	0.053 (2)	0.578 (8)
F4_4	−0.2106 (8)	0.3396 (3)	0.40188 (19)	0.081 (2)	0.578 (8)
F5_4	−0.0235 (7)	0.3871 (5)	0.4412 (4)	0.088 (3)	0.578 (8)
F6_4	−0.1775 (14)	0.4411 (7)	0.4518 (8)	0.053 (3)	0.578 (8)
C4_4	−0.3257 (10)	0.3349 (5)	0.4974 (4)	0.073 (3)	0.578 (8)
F7_4	−0.3619 (8)	0.2761 (4)	0.4609 (4)	0.102 (3)	0.578 (8)
F8_4	−0.3953 (19)	0.3752 (9)	0.4828 (8)	0.103 (5)	0.578 (8)
F9_4	−0.3586 (10)	0.3327 (5)	0.5470 (4)	0.104 (3)	0.578 (8)
O1_5	0.3558 (8)	0.3351 (4)	0.1662 (2)	0.0405 (9)	0.842 (3)
C1_5	0.4120 (5)	0.3019 (2)	0.1219 (2)	0.0418 (9)	0.842 (3)
C2_5	0.5545 (6)	0.3007 (3)	0.1377 (2)	0.0567 (14)	0.842 (3)
F1_5	0.6293 (3)	0.35867 (17)	0.14337 (16)	0.0698 (10)	0.842 (3)
F2_5	0.6034 (4)	0.2554 (2)	0.10028 (18)	0.0644 (11)	0.842 (3)
F3_5	0.5654 (4)	0.2883 (2)	0.18611 (16)	0.0615 (11)	0.842 (3)
C3_5	0.3401 (6)	0.2305 (2)	0.0984 (2)	0.0531 (12)	0.842 (3)
F4_5	0.2136 (4)	0.2275 (2)	0.0978 (2)	0.0721 (11)	0.842 (3)
F5_5	0.3718 (4)	0.19280 (15)	0.12858 (14)	0.0631 (9)	0.842 (3)
F6_5	0.3654 (4)	0.20196 (18)	0.04603 (14)	0.0670 (10)	0.842 (3)
C4_5	0.4085 (6)	0.3377 (3)	0.0774 (2)	0.0565 (14)	0.842 (3)
F7_5	0.2896 (4)	0.32478 (18)	0.05213 (14)	0.0729 (11)	0.842 (3)
F8_5	0.4886 (4)	0.3197 (2)	0.03798 (15)	0.0702 (12)	0.842 (3)
F9_5	0.4386 (4)	0.40232 (15)	0.09910 (13)	0.0594 (9)	0.842 (3)
O1_6	0.365 (4)	0.3351 (19)	0.1717 (11)	0.0405 (9)	0.158 (3)
C1_6	0.3909 (15)	0.2976 (8)	0.1220 (8)	0.0418 (9)	0.158 (3)
C2_6	0.4360 (18)	0.3424 (9)	0.0854 (8)	0.057 (4)	0.158 (3)
F1_6	0.3588 (19)	0.3859 (8)	0.0899 (7)	0.0594 (9)	0.158 (3)
F2_6	0.423 (2)	0.3098 (11)	0.0317 (7)	0.0702 (12)	0.158 (3)
F3_6	0.5563 (16)	0.3741 (8)	0.1017 (8)	0.071 (4)	0.158 (3)
C3_6	0.5003 (16)	0.2601 (8)	0.1281 (7)	0.049 (4)	0.158 (3)
F4_6	0.459 (2)	0.2096 (7)	0.1467 (7)	0.0631 (9)	0.158 (3)
F5_6	0.592 (2)	0.2988 (12)	0.1659 (10)	0.071 (7)	0.158 (3)
F6_6	0.548 (2)	0.2374 (12)	0.0797 (8)	0.072 (6)	0.158 (3)
C4_6	0.2688 (17)	0.2473 (9)	0.0935 (7)	0.057 (5)	0.158 (3)
F7_6	0.211 (2)	0.2201 (12)	0.1293 (8)	0.096 (8)	0.158 (3)
F8_6	0.296 (2)	0.2007 (10)	0.0511 (8)	0.0670 (10)	0.158 (3)
F9_6	0.189 (2)	0.2765 (12)	0.0717 (10)	0.110 (8)	0.158 (3)
O1_7	0.2048 (3)	0.39037 (16)	0.24907 (13)	0.0382 (7)	0.855 (3)
C1_7	0.0870 (4)	0.3970 (2)	0.2309 (2)	0.0431 (9)	0.855 (3)
C2_7	0.0862 (6)	0.3999 (3)	0.1699 (3)	0.0648 (16)	0.855 (3)
F1_7	0.1934 (4)	0.4362 (2)	0.16227 (18)	0.0782 (11)	0.855 (3)
F2_7	−0.0122 (4)	0.4259 (2)	0.15611 (17)	0.0783 (11)	0.855 (3)
F3_7	0.0784 (4)	0.3406 (2)	0.13374 (14)	0.0774 (11)	0.855 (3)
C3_7	−0.0173 (5)	0.3381 (3)	0.2316 (3)	0.0583 (13)	0.855 (3)
F4_7	−0.0451 (5)	0.3419 (4)	0.2834 (2)	0.0829 (16)	0.855 (3)
F5_7	0.0231 (3)	0.28246 (15)	0.21197 (18)	0.0748 (11)	0.855 (3)
F6_7	−0.1279 (3)	0.33514 (18)	0.20280 (18)	0.0730 (11)	0.855 (3)
C4_7	0.0537 (5)	0.4612 (3)	0.2681 (3)	0.0627 (15)	0.855 (3)
F7_7	0.0823 (4)	0.4670 (2)	0.32076 (15)	0.0765 (11)	0.855 (3)
F8_7	−0.0704 (3)	0.46481 (19)	0.2626 (2)	0.0799 (12)	0.855 (3)
F9_7	0.1226 (3)	0.51297 (15)	0.2570 (2)	0.0813 (13)	0.855 (3)
O1_8	0.1673 (17)	0.3612 (9)	0.2417 (8)	0.0382 (7)	0.145 (3)
C1_8	0.0760 (15)	0.3980 (7)	0.2412 (6)	0.0431 (9)	0.145 (3)
C2_8	0.0794 (18)	0.4485 (9)	0.2990 (7)	0.070 (4)	0.145 (3)
F1_8	−0.028 (2)	0.4730 (11)	0.3060 (10)	0.081 (6)	0.145 (3)
F2_8	0.180 (2)	0.4973 (10)	0.3063 (9)	0.092 (7)	0.145 (3)
F3_8	0.091 (2)	0.4217 (10)	0.3397 (7)	0.0765 (11)	0.145 (3)
C3_8	0.0996 (17)	0.4354 (9)	0.1976 (8)	0.0648 (16)	0.145 (3)
F4_8	0.074 (3)	0.3946 (11)	0.1468 (7)	0.074 (7)	0.145 (3)
F5_8	0.2232 (16)	0.4617 (12)	0.1998 (10)	0.0782 (11)	0.145 (3)
F6_8	0.034 (2)	0.4833 (11)	0.2031 (11)	0.088 (7)	0.145 (3)
C4_8	−0.0594 (15)	0.3534 (8)	0.2280 (8)	0.0583 (13)	0.145 (3)
F7_8	−0.059 (2)	0.3007 (9)	0.1857 (8)	0.086 (6)	0.145 (3)
F8_8	−0.1459 (15)	0.3860 (9)	0.2146 (9)	0.0730 (11)	0.145 (3)
F9_8	−0.094 (2)	0.3366 (14)	0.2727 (10)	0.073 (7)	0.145 (3)
O1_9	0.2095 (4)	0.8364 (2)	0.25548 (17)	0.0478 (10)	0.753 (6)
C1_9	0.0991 (6)	0.8608 (3)	0.2613 (3)	0.0444 (10)	0.753 (6)
C2_9	0.0699 (7)	0.8769 (4)	0.3230 (3)	0.0508 (11)	0.753 (6)
F1_9	0.1754 (6)	0.9090 (4)	0.3550 (3)	0.074 (2)	0.753 (6)
F2_9	−0.0198 (5)	0.9140 (4)	0.3344 (3)	0.0668 (19)	0.753 (6)
F3_9	0.0302 (14)	0.8222 (4)	0.3372 (5)	0.0654 (14)	0.753 (6)
C3_9	−0.0098 (6)	0.8059 (3)	0.2259 (3)	0.0516 (15)	0.753 (6)
F4_9	−0.0053 (7)	0.8009 (5)	0.1723 (3)	0.0649 (18)	0.753 (6)
F5_9	0.0028 (5)	0.74784 (18)	0.23086 (17)	0.0589 (10)	0.753 (6)
F6_9	−0.1269 (5)	0.8164 (3)	0.2402 (3)	0.0727 (17)	0.753 (6)
C4_9	0.1067 (7)	0.9227 (3)	0.2427 (3)	0.0640 (16)	0.753 (6)
F7_9	0.1700 (7)	0.9159 (3)	0.1971 (3)	0.0811 (17)	0.753 (6)
F8_9	−0.0095 (6)	0.9347 (4)	0.2314 (4)	0.0801 (19)	0.753 (6)
F9_9	0.1727 (6)	0.9748 (2)	0.2818 (2)	0.0842 (15)	0.753 (6)
O1_10	0.2299 (10)	0.8769 (7)	0.2690 (5)	0.0478 (10)	0.247 (6)
C1_10	0.1005 (14)	0.8734 (7)	0.2654 (6)	0.0444 (10)	0.247 (6)
C2_10	0.0651 (16)	0.9295 (7)	0.2444 (7)	0.0640 (16)	0.247 (6)
F1_10	0.0958 (14)	0.9865 (5)	0.2841 (5)	0.064 (3)	0.247 (6)
F2_10	−0.0600 (14)	0.9206 (11)	0.2297 (9)	0.064 (4)	0.247 (6)
F3_10	0.1279 (19)	0.9335 (12)	0.2003 (8)	0.089 (6)	0.247 (6)
C3_10	0.0304 (16)	0.8075 (7)	0.2246 (7)	0.0516 (15)	0.247 (6)
F4_10	0.0821 (14)	0.7581 (5)	0.2315 (5)	0.052 (3)	0.247 (6)
F5_10	0.043 (2)	0.8086 (14)	0.1726 (7)	0.071 (6)	0.247 (6)
F6_10	−0.0950 (14)	0.7950 (8)	0.2324 (7)	0.055 (4)	0.247 (6)
C4_10	0.0523 (18)	0.8786 (10)	0.3236 (7)	0.0508 (11)	0.247 (6)
F7_10	0.044 (4)	0.8207 (13)	0.3333 (17)	0.0654 (14)	0.247 (6)
F8_10	−0.0620 (15)	0.8962 (11)	0.3290 (7)	0.056 (5)	0.247 (6)
F9_10	0.1341 (15)	0.9214 (9)	0.3631 (6)	0.054 (4)	0.247 (6)
O1_11	0.3069 (4)	0.2741 (2)	0.24877 (17)	0.0419 (8)	0.925 (2)
C1_11	0.3061 (4)	0.2383 (2)	0.28453 (18)	0.0431 (9)	0.925 (2)
C2_11	0.2029 (5)	0.1743 (2)	0.2607 (2)	0.0526 (11)	0.925 (2)
F1_11	0.0848 (3)	0.18587 (18)	0.27116 (16)	0.0742 (9)	0.925 (2)
F2_11	0.2232 (4)	0.12799 (15)	0.28491 (15)	0.0711 (9)	0.925 (2)
F3_11	0.2067 (3)	0.14881 (13)	0.20762 (12)	0.0539 (7)	0.925 (2)
C3_11	0.4400 (5)	0.2193 (2)	0.28939 (19)	0.0541 (11)	0.925 (2)
F4_11	0.5345 (3)	0.27126 (14)	0.29432 (13)	0.0598 (8)	0.925 (2)
F5_11	0.4602 (4)	0.1742 (2)	0.2439 (2)	0.0584 (12)	0.925 (2)
F6_11	0.4587 (3)	0.19788 (17)	0.33226 (13)	0.0684 (9)	0.925 (2)
C4_11	0.2673 (5)	0.2762 (2)	0.34200 (18)	0.0540 (11)	0.925 (2)
F7_11	0.3735 (4)	0.32049 (16)	0.36976 (12)	0.0743 (10)	0.925 (2)
F8_11	0.2312 (4)	0.2379 (2)	0.37334 (15)	0.0617 (9)	0.925 (2)
F9_11	0.1762 (4)	0.30868 (18)	0.33791 (13)	0.0754 (11)	0.925 (2)
O1_12	0.319 (5)	0.268 (2)	0.2449 (18)	0.0419 (8)	0.075 (2)
C1_12	0.289 (2)	0.2325 (11)	0.2797 (10)	0.0431 (9)	0.075 (2)
C2_12	0.141 (2)	0.2099 (12)	0.2777 (10)	0.0526 (11)	0.075 (2)
F1_12	0.096 (4)	0.2527 (16)	0.3180 (13)	0.081 (9)	0.075 (2)
F2_12	0.114 (3)	0.1489 (11)	0.2820 (12)	0.045 (6)	0.075 (2)
F3_12	0.087 (3)	0.2072 (14)	0.2284 (10)	0.0539 (7)	0.075 (2)
C3_12	0.343 (3)	0.1675 (13)	0.2633 (10)	0.0541 (11)	0.075 (2)
F4_12	0.459 (4)	0.177 (3)	0.245 (2)	0.065 (13)	0.075 (2)
F5_12	0.264 (5)	0.1237 (18)	0.2238 (16)	0.120 (15)	0.075 (2)
F6_12	0.355 (4)	0.1480 (17)	0.3086 (13)	0.0684 (9)	0.075 (2)
C4_12	0.346 (3)	0.2693 (12)	0.3397 (10)	0.0540 (11)	0.075 (2)
F7_12	0.468 (2)	0.2580 (18)	0.3418 (13)	0.065 (7)	0.075 (2)
F8_12	0.285 (4)	0.243 (2)	0.3763 (16)	0.074 (11)	0.075 (2)
F9_12	0.331 (5)	0.3315 (13)	0.3550 (15)	0.0743 (10)	0.075 (2)
O1_13	0.4552 (4)	0.91819 (16)	0.29258 (13)	0.0663 (10)	
C1_13	0.5454 (4)	0.97273 (17)	0.30319 (15)	0.0421 (9)	
C2_13	0.5012 (5)	1.0257 (2)	0.3510 (2)	0.0625 (12)	
F1_13	0.4087 (4)	1.0500 (2)	0.3324 (2)	0.1161 (15)	
F2_13	0.5991 (4)	1.07567 (14)	0.37410 (13)	0.0802 (10)	
F3_13	0.4593 (3)	1.00136 (19)	0.39095 (13)	0.0839 (10)	
C3_13	0.6751 (5)	0.9605 (3)	0.3224 (2)	0.0851 (18)	
F4_13	0.6932 (5)	0.9019 (2)	0.2904 (2)	0.135 (2)	
F5_13	0.6776 (4)	0.95838 (19)	0.37370 (15)	0.1031 (14)	
F6_13	0.7717 (4)	1.0067 (3)	0.3194 (2)	0.137 (2)	
C4_13	0.5574 (6)	0.9970 (2)	0.2519 (2)	0.0780 (16)	
F7_13	0.6276 (4)	0.9631 (2)	0.21572 (14)	0.1135 (16)	
F8_13	0.6083 (6)	1.06036 (17)	0.26467 (15)	0.1253 (19)	
F9_13	0.4429 (5)	0.98865 (19)	0.22557 (15)	0.1091 (15)	
O1_20	0.0911 (3)	0.18535 (13)	0.48740 (11)	0.0420 (6)	
C1_20	0.1602 (4)	0.13777 (18)	0.48589 (15)	0.0402 (8)	
C2_20	0.3056 (4)	0.1666 (2)	0.48800 (18)	0.0534 (10)	
F1_20	0.3367 (3)	0.17476 (19)	0.43980 (13)	0.0771 (9)	
F2_20	0.3807 (3)	0.12744 (17)	0.49988 (13)	0.0703 (8)	
F3_20	0.3353 (3)	0.22361 (15)	0.52596 (14)	0.0724 (8)	
C3_20	0.1346 (4)	0.1119 (2)	0.53654 (18)	0.0513 (10)	
F4_20	0.0089 (3)	0.09857 (16)	0.54171 (12)	0.0649 (7)	
F5_20	0.1878 (3)	0.15631 (16)	0.58288 (11)	0.0667 (8)	
F6_20	0.1794 (3)	0.05664 (16)	0.53240 (14)	0.0741 (9)	
C4_20	0.1190 (5)	0.0809 (2)	0.43202 (18)	0.0571 (11)	
F7_20	0.0022 (3)	0.04792 (14)	0.43341 (13)	0.0765 (9)	
F8_20	0.2000 (4)	0.03895 (16)	0.42263 (14)	0.0868 (11)	
F9_20	0.1152 (3)	0.10347 (15)	0.38865 (11)	0.0703 (8)	
O1_14	0.4146 (4)	0.78006 (14)	0.26815 (12)	0.0574 (8)	
C1_14	0.4204 (4)	0.74872 (18)	0.30648 (15)	0.0432 (9)	
C2_14	0.2980 (5)	0.6961 (2)	0.30012 (19)	0.0560 (11)	
F1_14	0.2017 (3)	0.72441 (17)	0.32346 (14)	0.0775 (9)	
F2_14	0.3140 (4)	0.65073 (15)	0.32373 (15)	0.0873 (11)	
F3_14	0.2586 (3)	0.66657 (15)	0.24728 (13)	0.0769 (9)	
C3_14	0.5374 (5)	0.7139 (3)	0.29604 (19)	0.0658 (13)	
F4_14	0.6396 (3)	0.7514 (2)	0.28722 (16)	0.1036 (13)	
F5_14	0.5119 (3)	0.66080 (16)	0.25173 (12)	0.0740 (9)	
F6_14	0.5709 (4)	0.6934 (2)	0.33915 (14)	0.0997 (13)	
C4_14	0.4361 (5)	0.7967 (2)	0.36586 (17)	0.0537 (11)	
F7_14	0.5550 (3)	0.83125 (18)	0.37756 (12)	0.0845 (11)	
F8_14	0.4122 (3)	0.76666 (15)	0.40469 (10)	0.0647 (7)	
F9_14	0.3562 (3)	0.83872 (13)	0.37044 (11)	0.0630 (7)	
O1_15	0.4613 (3)	0.39752 (15)	0.27912 (13)	0.0392 (7)	0.889 (3)
C1_15	0.5258 (4)	0.4588 (2)	0.30495 (18)	0.0393 (8)	0.889 (3)
C2_15	0.4367 (5)	0.4996 (2)	0.3429 (2)	0.0521 (11)	0.889 (3)
F1_15	0.3613 (3)	0.52320 (14)	0.31417 (13)	0.0561 (8)	0.889 (3)
F2_15	0.5037 (3)	0.55049 (16)	0.38273 (14)	0.0717 (10)	0.889 (3)
F3_15	0.3606 (4)	0.46267 (18)	0.36747 (13)	0.0633 (8)	0.889 (3)
C3_15	0.6396 (5)	0.4516 (2)	0.3418 (2)	0.0507 (11)	0.889 (3)
F4_15	0.6985 (3)	0.40410 (16)	0.31485 (14)	0.0572 (8)	0.889 (3)
F5_15	0.5962 (4)	0.4367 (2)	0.38689 (14)	0.0654 (10)	0.889 (3)
F6_15	0.7270 (3)	0.50649 (15)	0.35952 (14)	0.0635 (9)	0.889 (3)
C4_15	0.5774 (5)	0.4934 (2)	0.2625 (2)	0.0471 (11)	0.889 (3)
F7_15	0.6764 (3)	0.4694 (2)	0.23909 (15)	0.0610 (8)	0.889 (3)
F8_15	0.6152 (4)	0.55797 (16)	0.28560 (16)	0.0656 (10)	0.889 (3)
F9_15	0.4859 (5)	0.4846 (3)	0.2222 (2)	0.0539 (12)	0.889 (3)
O1_16	0.4142 (19)	0.4141 (11)	0.2826 (10)	0.0392 (7)	0.111 (3)
C1_16	0.5307 (17)	0.4526 (8)	0.3012 (7)	0.0393 (8)	0.111 (3)
C2_16	0.589 (2)	0.4785 (11)	0.2546 (8)	0.0471 (11)	0.111 (3)
F1_16	0.640 (2)	0.4336 (13)	0.2186 (10)	0.061 (6)	0.111 (3)
F2_16	0.682 (3)	0.5304 (11)	0.2749 (10)	0.067 (7)	0.111 (3)
F3_16	0.500 (4)	0.497 (2)	0.2275 (17)	0.060 (10)	0.111 (3)
C3_16	0.5135 (19)	0.5115 (9)	0.3508 (7)	0.0521 (11)	0.111 (3)
F4_16	0.441 (2)	0.4903 (11)	0.3863 (8)	0.056 (5)	0.111 (3)
F5_16	0.457 (2)	0.5532 (9)	0.3334 (10)	0.066 (6)	0.111 (3)
F6_16	0.624 (2)	0.5453 (13)	0.3789 (9)	0.084 (9)	0.111 (3)
C4_16	0.6214 (18)	0.4125 (9)	0.3203 (8)	0.0507 (11)	0.111 (3)
F7_16	0.585 (2)	0.4015 (15)	0.3670 (9)	0.059 (6)	0.111 (3)
F8_16	0.7443 (17)	0.4418 (13)	0.3276 (10)	0.061 (6)	0.111 (3)
F9_16	0.615 (2)	0.3546 (9)	0.2830 (9)	0.058 (6)	0.111 (3)
O1_17	0.4074 (4)	0.83710 (15)	0.18350 (12)	0.0578 (9)	
C1_17	0.4200 (4)	0.79599 (18)	0.13319 (15)	0.0455 (9)	
C2_17	0.2999 (4)	0.7415 (2)	0.11231 (17)	0.0500 (10)	
F1_17	0.2589 (3)	0.71744 (15)	0.15286 (11)	0.0614 (7)	
F2_17	0.3213 (3)	0.69159 (13)	0.07043 (10)	0.0595 (7)	
F3_17	0.2031 (3)	0.76530 (19)	0.09499 (13)	0.0762 (9)	
C3_17	0.4399 (6)	0.8372 (2)	0.09210 (17)	0.0627 (13)	
F4_17	0.5598 (4)	0.87170 (15)	0.09996 (12)	0.0864 (11)	
F5_17	0.3609 (4)	0.87867 (16)	0.09903 (12)	0.0902 (12)	
F6_17	0.4226 (3)	0.80001 (13)	0.03915 (10)	0.0638 (8)	
C4_17	0.5388 (4)	0.7637 (2)	0.13631 (17)	0.0535 (10)	
F7_17	0.6367 (3)	0.80534 (17)	0.16750 (13)	0.0770 (9)	
F8_17	0.5803 (3)	0.73920 (15)	0.08622 (11)	0.0646 (7)	
F9_17	0.5127 (3)	0.71396 (13)	0.15780 (11)	0.0566 (6)	
O1_18	−0.1181 (3)	0.53605 (18)	0.04762 (16)	0.0387 (7)	0.934 (4)
C1_18	−0.2005 (4)	0.5669 (2)	0.08087 (17)	0.0408 (8)	0.934 (4)
C2_18	−0.3344 (4)	0.5533 (2)	0.04792 (19)	0.0485 (10)	0.934 (4)
F1_18	−0.3199 (4)	0.5608 (2)	−0.00215 (13)	0.0608 (10)	0.934 (4)
F2_18	−0.4127 (3)	0.59257 (17)	0.07259 (13)	0.0601 (8)	0.934 (4)
F3_18	−0.3924 (3)	0.49176 (15)	0.04025 (16)	0.0684 (9)	0.934 (4)
C3_18	−0.2163 (5)	0.5408 (3)	0.1315 (2)	0.0588 (12)	0.934 (4)
F4_18	−0.1129 (4)	0.5641 (2)	0.16735 (14)	0.0828 (12)	0.934 (4)
F5_18	−0.2319 (4)	0.47516 (19)	0.11606 (18)	0.0694 (10)	0.934 (4)
F6_18	−0.3188 (5)	0.5575 (3)	0.15883 (18)	0.0810 (14)	0.934 (4)
C4_18	−0.1479 (5)	0.6420 (2)	0.0998 (2)	0.0558 (12)	0.934 (4)
F7_18	−0.0237 (4)	0.6526 (2)	0.1162 (2)	0.0775 (14)	0.934 (4)
F8_18	−0.2088 (3)	0.67357 (17)	0.14187 (16)	0.0738 (11)	0.934 (4)
F9_18	−0.1614 (4)	0.66726 (16)	0.05871 (18)	0.0742 (11)	0.934 (4)
O1_19	−0.102 (4)	0.544 (2)	0.0490 (18)	0.0387 (7)	0.066 (4)
C1_19	−0.189 (2)	0.5684 (12)	0.0824 (10)	0.0408 (8)	0.066 (4)
C2_19	−0.279 (3)	0.5978 (15)	0.0508 (12)	0.0485 (10)	0.066 (4)
F1_19	−0.211 (4)	0.647 (2)	0.037 (2)	0.076 (10)	0.066 (4)
F2_19	−0.368 (3)	0.622 (2)	0.0821 (17)	0.0601 (8)	0.066 (4)
F3_19	−0.336 (6)	0.554 (2)	0.0049 (17)	0.0608 (10)	0.066 (4)
C3_19	−0.268 (3)	0.5132 (14)	0.1018 (13)	0.0588 (12)	0.066 (4)
F4_19	−0.187 (5)	0.485 (3)	0.123 (3)	0.0694 (10)	0.066 (4)
F5_19	−0.343 (5)	0.470 (2)	0.0603 (17)	0.090 (12)	0.066 (4)
F6_19	−0.344 (5)	0.537 (2)	0.1407 (18)	0.065 (11)	0.066 (4)
C4_19	−0.115 (3)	0.6222 (14)	0.1329 (12)	0.0558 (12)	0.066 (4)
F7_19	−0.037 (5)	0.596 (2)	0.1593 (15)	0.083 (11)	0.066 (4)
F8_19	−0.193 (4)	0.649 (2)	0.1687 (17)	0.086 (12)	0.066 (4)
F9_19	−0.043 (5)	0.669 (2)	0.116 (2)	0.079 (13)	0.066 (4)

### Tetrakis(acetonitrile)bis(nonafluoro-tert-butanolato)titanium(III) tetrakis(nonafluoro-tert-butanolato)aluminate Atomic displacement parameters (Å^2^)

**Table:**

	*U*^11^	*U*^22^	*U*^33^	*U*^12^	*U*^13^	*U*^23^
Ti1	0.0620 (7)	0.0407 (5)	0.0452 (6)	−0.0110 (5)	−0.0106 (5)	0.0176 (5)
Al1	0.0343 (6)	0.0354 (5)	0.0290 (5)	−0.0030 (4)	−0.0002 (4)	0.0058 (4)
N1	0.0434 (19)	0.0413 (17)	0.0391 (17)	0.0065 (15)	−0.0009 (14)	0.0154 (14)
C1	0.042 (2)	0.046 (2)	0.0349 (19)	0.0114 (18)	−0.0006 (16)	0.0109 (16)
Ti2	0.0340 (5)	0.0334 (4)	0.0318 (4)	−0.0020 (4)	−0.0025 (4)	0.0094 (4)
Al2	0.0331 (6)	0.0351 (5)	0.0297 (5)	0.0019 (4)	−0.0006 (4)	0.0112 (4)
N2	0.0414 (18)	0.0439 (17)	0.0407 (18)	0.0055 (14)	0.0006 (15)	0.0161 (14)
C2	0.047 (2)	0.045 (2)	0.053 (2)	0.0002 (18)	0.001 (2)	0.0147 (19)
C7	0.041 (2)	0.047 (2)	0.044 (2)	0.0004 (17)	0.0057 (18)	0.0121 (18)
N4	0.0457 (19)	0.0411 (17)	0.0411 (18)	0.0043 (14)	0.0004 (15)	0.0118 (14)
C4	0.054 (3)	0.083 (3)	0.043 (2)	−0.017 (3)	−0.009 (2)	0.011 (2)
Ti3	0.0394 (4)	0.0353 (3)	0.0372 (3)	0.0068 (3)	0.0007 (3)	0.0116 (3)
N3	0.0374 (18)	0.0450 (18)	0.0447 (18)	0.0063 (15)	0.0021 (14)	0.0142 (15)
C3	0.037 (2)	0.050 (2)	0.046 (2)	−0.0050 (17)	−0.0010 (18)	0.0177 (19)
N5	0.069 (3)	0.0428 (19)	0.053 (2)	−0.0083 (18)	−0.010 (2)	0.0167 (17)
C5	0.039 (2)	0.044 (2)	0.0367 (19)	0.0075 (18)	−0.0003 (16)	0.0105 (16)
N6	0.070 (3)	0.0431 (19)	0.050 (2)	−0.0095 (18)	−0.0057 (19)	0.0195 (16)
C6	0.052 (3)	0.043 (2)	0.047 (2)	−0.0021 (18)	−0.0023 (19)	0.0109 (18)
N7	0.0416 (18)	0.0404 (17)	0.0371 (16)	−0.0008 (14)	0.0005 (14)	0.0132 (13)
N8	0.0417 (18)	0.0375 (16)	0.0353 (16)	−0.0013 (13)	−0.0029 (14)	0.0089 (13)
C8	0.056 (3)	0.075 (3)	0.043 (2)	−0.009 (2)	−0.004 (2)	0.016 (2)
C9	0.068 (3)	0.035 (2)	0.050 (2)	−0.0034 (19)	−0.009 (2)	0.0104 (18)
C10	0.074 (3)	0.053 (3)	0.060 (3)	0.011 (2)	−0.016 (3)	0.006 (2)
C11	0.059 (3)	0.048 (2)	0.052 (2)	−0.008 (2)	−0.006 (2)	0.022 (2)
C12	0.088 (4)	0.068 (3)	0.090 (4)	−0.016 (3)	−0.008 (3)	0.053 (3)
C13	0.050 (2)	0.043 (2)	0.039 (2)	−0.0040 (18)	−0.0010 (18)	0.0118 (17)
C14	0.096 (4)	0.061 (3)	0.063 (3)	−0.020 (3)	0.001 (3)	0.033 (3)
C15	0.042 (2)	0.0370 (18)	0.0357 (19)	0.0018 (16)	−0.0011 (17)	0.0094 (15)
C16	0.044 (2)	0.051 (2)	0.045 (2)	−0.0025 (19)	−0.0123 (19)	0.0102 (18)
O1_1	0.064 (3)	0.046 (3)	0.052 (2)	−0.0059 (17)	−0.006 (2)	0.0177 (18)
C1_1	0.060 (3)	0.056 (3)	0.059 (3)	−0.005 (2)	−0.002 (2)	0.023 (2)
C2_1	0.060 (6)	0.094 (7)	0.084 (7)	0.018 (4)	−0.030 (5)	−0.011 (5)
F1_1	0.136 (9)	0.222 (14)	0.092 (6)	0.092 (9)	−0.073 (6)	−0.038 (6)
F2_1	0.062 (4)	0.064 (4)	0.147 (7)	0.000 (3)	−0.025 (4)	−0.030 (4)
F3_1	0.067 (5)	0.056 (4)	0.204 (10)	−0.020 (3)	−0.009 (5)	0.012 (5)
C3_1	0.065 (5)	0.072 (5)	0.052 (5)	−0.003 (4)	−0.013 (4)	0.009 (4)
F4_1	0.048 (4)	0.113 (6)	0.103 (7)	−0.008 (4)	−0.024 (4)	−0.035 (5)
F5_1	0.144 (8)	0.140 (7)	0.126 (7)	0.022 (6)	−0.004 (5)	0.094 (6)
F6_1	0.097 (5)	0.076 (4)	0.077 (4)	0.036 (4)	−0.011 (3)	0.010 (3)
C4_1	0.114 (9)	0.108 (8)	0.124 (10)	0.042 (6)	0.054 (6)	0.073 (7)
F7_1	0.206 (12)	0.105 (6)	0.180 (10)	−0.042 (7)	0.061 (9)	0.061 (6)
F8_1	0.109 (7)	0.134 (8)	0.121 (7)	0.052 (6)	0.054 (5)	0.079 (6)
F9_1	0.207 (13)	0.222 (14)	0.189 (13)	0.140 (11)	0.103 (10)	0.178 (12)
O1_2	0.064 (3)	0.046 (3)	0.052 (2)	−0.0059 (17)	−0.006 (2)	0.0177 (18)
C1_2	0.060 (3)	0.056 (3)	0.059 (3)	−0.005 (2)	−0.002 (2)	0.023 (2)
C2_2	0.143 (11)	0.077 (7)	0.042 (6)	0.002 (8)	0.007 (6)	0.017 (5)
F1_2	0.141 (11)	0.074 (5)	0.104 (8)	−0.014 (6)	0.073 (7)	0.041 (5)
F2_2	0.276 (18)	0.127 (10)	0.084 (8)	0.003 (13)	0.088 (11)	−0.008 (7)
F3_2	0.212 (12)	0.200 (16)	0.118 (9)	−0.020 (11)	−0.095 (9)	0.069 (10)
C3_2	0.094 (9)	0.053 (6)	0.179 (13)	0.021 (7)	0.062 (9)	0.036 (7)
F4_2	0.204 (18)	0.135 (12)	0.33 (2)	0.056 (12)	0.098 (15)	0.179 (15)
F5_2	0.103 (8)	0.050 (5)	0.168 (14)	−0.038 (5)	0.036 (8)	−0.020 (6)
F6_2	0.166 (15)	0.147 (16)	0.28 (2)	0.037 (13)	0.114 (16)	−0.046 (14)
C4_2	0.060 (7)	0.217 (13)	0.084 (9)	−0.009 (9)	−0.001 (6)	0.057 (10)
F7_2	0.153 (15)	0.219 (17)	0.084 (8)	0.125 (13)	0.003 (8)	0.069 (11)
F8_2	0.046 (7)	0.43 (3)	0.182 (15)	−0.013 (12)	0.022 (9)	−0.055 (18)
F9_2	0.168 (12)	0.242 (12)	0.126 (11)	−0.151 (10)	−0.063 (9)	0.041 (10)
O1_3	0.064 (11)	0.033 (7)	0.038 (10)	0.017 (7)	−0.004 (8)	0.005 (5)
C1_3	0.051 (2)	0.050 (2)	0.044 (2)	0.0201 (19)	0.0067 (18)	0.0222 (17)
C2_3	0.051 (7)	0.046 (6)	0.051 (5)	0.017 (5)	−0.001 (5)	0.021 (4)
F1_3	0.057 (4)	0.054 (4)	0.151 (10)	0.002 (3)	−0.025 (5)	0.035 (5)
F2_3	0.079 (12)	0.063 (9)	0.066 (10)	0.021 (8)	−0.016 (8)	0.032 (7)
F3_3	0.145 (12)	0.070 (5)	0.063 (5)	0.039 (6)	0.045 (6)	0.031 (4)
C3_3	0.050 (6)	0.106 (10)	0.151 (9)	0.019 (6)	0.007 (7)	0.094 (8)
F4_3	0.051 (6)	0.166 (16)	0.245 (18)	0.012 (10)	0.020 (14)	0.161 (16)
F5_3	0.080 (6)	0.089 (6)	0.165 (8)	−0.030 (5)	−0.065 (6)	0.063 (6)
F6_3	0.065 (11)	0.150 (12)	0.202 (18)	0.057 (11)	0.061 (12)	0.130 (11)
C4_3	0.169 (11)	0.076 (8)	0.046 (6)	0.081 (8)	0.024 (6)	0.024 (5)
F7_3	0.277 (18)	0.153 (12)	0.091 (9)	0.127 (14)	0.106 (12)	0.073 (9)
F8_3	0.150 (12)	0.058 (5)	0.054 (8)	0.046 (6)	−0.012 (8)	−0.001 (4)
F9_3	0.222 (12)	0.071 (6)	0.071 (7)	0.057 (7)	−0.063 (8)	−0.002 (5)
O1_4	0.035 (5)	0.046 (5)	0.039 (7)	0.005 (4)	0.002 (5)	0.020 (5)
C1_4	0.051 (2)	0.050 (2)	0.044 (2)	0.0201 (19)	0.0067 (18)	0.0222 (17)
C2_4	0.087 (6)	0.059 (5)	0.052 (5)	0.031 (4)	0.011 (5)	0.019 (4)
F1_4	0.129 (7)	0.062 (4)	0.039 (3)	0.034 (4)	0.005 (4)	0.012 (2)
F2_4	0.201 (12)	0.097 (8)	0.076 (8)	0.110 (9)	0.043 (8)	0.035 (7)
F3_4	0.094 (4)	0.051 (3)	0.102 (5)	−0.009 (3)	−0.021 (4)	0.013 (3)
C3_4	0.056 (5)	0.062 (5)	0.054 (5)	0.021 (4)	0.005 (4)	0.030 (4)
F4_4	0.132 (7)	0.083 (4)	0.039 (2)	0.055 (4)	−0.009 (3)	0.015 (2)
F5_4	0.062 (4)	0.152 (9)	0.105 (6)	0.052 (5)	0.043 (4)	0.103 (7)
F6_4	0.052 (6)	0.056 (6)	0.062 (6)	0.011 (5)	0.007 (5)	0.033 (4)
C4_4	0.046 (5)	0.086 (6)	0.105 (7)	0.022 (4)	0.002 (5)	0.055 (5)
F7_4	0.063 (4)	0.091 (4)	0.149 (7)	−0.012 (3)	−0.049 (5)	0.050 (5)
F8_4	0.050 (6)	0.145 (8)	0.172 (11)	0.041 (6)	0.027 (6)	0.120 (8)
F9_4	0.064 (5)	0.151 (9)	0.151 (7)	0.039 (6)	0.043 (6)	0.114 (7)
O1_5	0.049 (2)	0.0414 (14)	0.0321 (17)	0.0098 (13)	0.0037 (16)	0.0123 (13)
C1_5	0.050 (2)	0.0404 (19)	0.0317 (17)	0.0015 (17)	0.0024 (17)	0.0090 (15)
C2_5	0.054 (3)	0.060 (3)	0.051 (3)	0.006 (3)	0.011 (3)	0.011 (3)
F1_5	0.053 (2)	0.065 (2)	0.083 (2)	−0.0066 (16)	0.0128 (18)	0.0160 (18)
F2_5	0.062 (3)	0.066 (2)	0.064 (2)	0.015 (2)	0.0213 (19)	0.0139 (19)
F3_5	0.051 (2)	0.086 (3)	0.054 (2)	0.0205 (19)	0.0057 (18)	0.0260 (19)
C3_5	0.063 (3)	0.046 (3)	0.045 (3)	0.003 (2)	0.007 (2)	0.009 (2)
F4_5	0.054 (2)	0.065 (2)	0.082 (3)	−0.0099 (18)	0.000 (2)	0.011 (2)
F5_5	0.088 (3)	0.0439 (16)	0.0589 (19)	0.0072 (17)	0.0158 (18)	0.0200 (14)
F6_5	0.082 (3)	0.0547 (17)	0.0483 (16)	0.001 (2)	0.006 (2)	−0.0029 (13)
C4_5	0.087 (4)	0.046 (3)	0.035 (3)	0.010 (3)	0.004 (2)	0.012 (2)
F7_5	0.093 (3)	0.076 (2)	0.0494 (18)	0.013 (2)	−0.0144 (18)	0.0213 (17)
F8_5	0.106 (4)	0.065 (2)	0.0432 (16)	0.018 (3)	0.029 (2)	0.0192 (15)
F9_5	0.089 (3)	0.0453 (16)	0.0451 (16)	0.0027 (17)	0.0140 (18)	0.0196 (13)
O1_6	0.049 (2)	0.0414 (14)	0.0321 (17)	0.0098 (13)	0.0037 (16)	0.0123 (13)
C1_6	0.050 (2)	0.0404 (19)	0.0317 (17)	0.0015 (17)	0.0024 (17)	0.0090 (15)
C2_6	0.082 (9)	0.048 (10)	0.043 (7)	0.004 (6)	0.020 (8)	0.017 (6)
F1_6	0.089 (3)	0.0453 (16)	0.0451 (16)	0.0027 (17)	0.0140 (18)	0.0196 (13)
F2_6	0.106 (4)	0.065 (2)	0.0432 (16)	0.018 (3)	0.029 (2)	0.0192 (15)
F3_6	0.080 (8)	0.042 (8)	0.088 (11)	−0.009 (7)	0.032 (8)	0.025 (8)
C3_6	0.048 (9)	0.043 (9)	0.059 (10)	0.006 (6)	0.019 (7)	0.022 (7)
F4_6	0.088 (3)	0.0439 (16)	0.0589 (19)	0.0072 (17)	0.0158 (18)	0.0200 (14)
F5_6	0.043 (10)	0.088 (13)	0.076 (14)	0.000 (8)	0.008 (10)	0.024 (12)
F6_6	0.101 (19)	0.068 (15)	0.066 (11)	0.043 (13)	0.042 (11)	0.031 (9)
C4_6	0.048 (9)	0.061 (10)	0.045 (9)	−0.006 (6)	0.004 (7)	−0.003 (6)
F7_6	0.081 (15)	0.108 (16)	0.064 (11)	−0.055 (11)	0.003 (10)	0.012 (10)
F8_6	0.082 (3)	0.0547 (17)	0.0483 (16)	0.001 (2)	0.006 (2)	−0.0029 (13)
F9_6	0.081 (14)	0.140 (19)	0.096 (16)	0.028 (13)	−0.031 (12)	0.015 (13)
O1_7	0.0300 (16)	0.0382 (17)	0.0420 (16)	−0.0018 (13)	−0.0027 (13)	0.0102 (15)
C1_7	0.0284 (19)	0.0427 (19)	0.055 (2)	0.0030 (15)	−0.0041 (18)	0.0121 (18)
C2_7	0.050 (3)	0.084 (4)	0.072 (4)	0.018 (3)	−0.007 (3)	0.038 (3)
F1_7	0.063 (2)	0.106 (3)	0.088 (3)	0.014 (2)	0.008 (2)	0.064 (3)
F2_7	0.062 (2)	0.097 (3)	0.088 (3)	0.024 (2)	−0.0157 (19)	0.042 (2)
F3_7	0.067 (2)	0.104 (3)	0.0538 (19)	0.028 (2)	−0.0138 (17)	0.0061 (19)
C3_7	0.033 (3)	0.057 (3)	0.082 (3)	−0.002 (2)	−0.005 (2)	0.022 (2)
F4_7	0.049 (3)	0.107 (4)	0.107 (3)	0.002 (3)	0.021 (2)	0.060 (3)
F5_7	0.053 (2)	0.0420 (16)	0.115 (3)	−0.0047 (14)	−0.021 (2)	0.0106 (17)
F6_7	0.0347 (16)	0.061 (2)	0.113 (3)	−0.0067 (14)	−0.0219 (17)	0.020 (2)
C4_7	0.038 (3)	0.059 (3)	0.080 (4)	0.011 (2)	−0.012 (3)	0.003 (3)
F7_7	0.058 (2)	0.085 (3)	0.067 (2)	0.020 (2)	−0.0007 (18)	−0.0105 (19)
F8_7	0.0398 (17)	0.077 (2)	0.106 (3)	0.0204 (16)	−0.0105 (19)	−0.004 (2)
F9_7	0.053 (2)	0.0419 (16)	0.139 (4)	0.0026 (14)	−0.024 (2)	0.0169 (19)
O1_8	0.0300 (16)	0.0382 (17)	0.0420 (16)	−0.0018 (13)	−0.0027 (13)	0.0102 (15)
C1_8	0.0284 (19)	0.0427 (19)	0.055 (2)	0.0030 (15)	−0.0041 (18)	0.0121 (18)
C2_8	0.058 (11)	0.065 (10)	0.069 (8)	0.011 (7)	0.010 (9)	−0.008 (6)
F1_8	0.084 (12)	0.075 (13)	0.099 (17)	0.034 (11)	0.050 (13)	0.035 (12)
F2_8	0.090 (13)	0.078 (12)	0.065 (13)	−0.022 (11)	0.002 (11)	−0.026 (8)
F3_8	0.058 (2)	0.085 (3)	0.067 (2)	0.020 (2)	−0.0007 (18)	−0.0105 (19)
C3_8	0.050 (3)	0.084 (4)	0.072 (4)	0.018 (3)	−0.007 (3)	0.038 (3)
F4_8	0.099 (18)	0.079 (11)	0.056 (8)	0.001 (10)	−0.017 (10)	0.053 (7)
F5_8	0.063 (2)	0.106 (3)	0.088 (3)	0.014 (2)	0.008 (2)	0.064 (3)
F6_8	0.088 (13)	0.093 (13)	0.131 (18)	0.043 (12)	0.040 (14)	0.088 (12)
C4_8	0.033 (3)	0.057 (3)	0.082 (3)	−0.002 (2)	−0.005 (2)	0.022 (2)
F7_8	0.098 (17)	0.056 (9)	0.081 (10)	−0.003 (9)	−0.042 (11)	−0.001 (8)
F8_8	0.0347 (16)	0.061 (2)	0.113 (3)	−0.0067 (14)	−0.0219 (17)	0.020 (2)
F9_8	0.057 (17)	0.063 (14)	0.116 (13)	0.025 (13)	0.035 (13)	0.045 (12)
O1_9	0.0330 (18)	0.050 (2)	0.051 (2)	0.0015 (18)	−0.0008 (15)	0.005 (2)
C1_9	0.034 (2)	0.047 (3)	0.044 (2)	0.0038 (19)	−0.0021 (17)	0.004 (2)
C2_9	0.043 (3)	0.055 (2)	0.046 (2)	0.007 (2)	−0.0041 (19)	0.0041 (18)
F1_9	0.055 (3)	0.086 (3)	0.056 (3)	0.017 (3)	−0.015 (3)	−0.018 (2)
F2_9	0.055 (3)	0.074 (4)	0.061 (3)	0.025 (3)	0.003 (3)	−0.003 (2)
F3_9	0.081 (4)	0.0658 (17)	0.052 (2)	0.0180 (18)	0.010 (2)	0.0182 (16)
C3_9	0.036 (4)	0.061 (3)	0.050 (2)	0.002 (3)	−0.007 (3)	0.009 (2)
F4_9	0.070 (5)	0.075 (3)	0.041 (2)	0.009 (3)	−0.012 (2)	0.008 (2)
F5_9	0.055 (3)	0.051 (2)	0.059 (2)	−0.0042 (19)	−0.004 (2)	0.0047 (16)
F6_9	0.037 (2)	0.086 (4)	0.078 (3)	0.008 (2)	−0.005 (2)	0.001 (3)
C4_9	0.063 (4)	0.062 (3)	0.068 (3)	0.007 (3)	0.008 (3)	0.023 (2)
F7_9	0.091 (5)	0.082 (4)	0.088 (3)	0.022 (3)	0.028 (3)	0.047 (3)
F8_9	0.085 (4)	0.077 (4)	0.089 (4)	0.030 (4)	0.004 (4)	0.033 (3)
F9_9	0.074 (3)	0.051 (2)	0.118 (4)	0.000 (2)	0.008 (3)	0.016 (2)
O1_10	0.0330 (18)	0.050 (2)	0.051 (2)	0.0015 (18)	−0.0008 (15)	0.005 (2)
C1_10	0.034 (2)	0.047 (3)	0.044 (2)	0.0038 (19)	−0.0021 (17)	0.004 (2)
C2_10	0.063 (4)	0.062 (3)	0.068 (3)	0.007 (3)	0.008 (3)	0.023 (2)
F1_10	0.051 (7)	0.045 (5)	0.094 (7)	0.008 (5)	−0.008 (6)	0.022 (5)
F2_10	0.058 (7)	0.079 (11)	0.064 (8)	0.014 (7)	0.002 (7)	0.035 (7)
F3_10	0.072 (10)	0.133 (16)	0.105 (9)	0.053 (10)	0.045 (8)	0.080 (10)
C3_10	0.036 (4)	0.061 (3)	0.050 (2)	0.002 (3)	−0.007 (3)	0.009 (2)
F4_10	0.053 (7)	0.041 (5)	0.051 (6)	0.004 (5)	−0.001 (6)	0.000 (4)
F5_10	0.075 (15)	0.089 (12)	0.033 (5)	0.013 (11)	−0.013 (7)	−0.003 (5)
F6_10	0.038 (6)	0.060 (9)	0.053 (7)	−0.010 (5)	−0.013 (6)	0.006 (7)
C4_10	0.043 (3)	0.055 (2)	0.046 (2)	0.007 (2)	−0.0041 (19)	0.0041 (18)
F7_10	0.081 (4)	0.0658 (17)	0.052 (2)	0.0180 (18)	0.010 (2)	0.0182 (16)
F8_10	0.055 (8)	0.090 (13)	0.042 (8)	0.028 (8)	0.020 (6)	0.041 (9)
F9_10	0.056 (9)	0.069 (9)	0.023 (4)	−0.015 (7)	0.010 (5)	0.002 (5)
O1_11	0.0509 (19)	0.0398 (16)	0.0370 (15)	0.0054 (13)	0.0071 (13)	0.0157 (13)
C1_11	0.053 (2)	0.0388 (19)	0.042 (2)	0.0064 (17)	0.0078 (17)	0.0209 (16)
C2_11	0.054 (3)	0.042 (2)	0.063 (3)	0.0005 (19)	0.014 (2)	0.021 (2)
F1_11	0.0514 (18)	0.076 (2)	0.098 (3)	0.0009 (16)	0.0137 (17)	0.035 (2)
F2_11	0.083 (2)	0.0553 (17)	0.083 (2)	0.0034 (16)	0.0129 (18)	0.0373 (16)
F3_11	0.0503 (16)	0.0486 (14)	0.0553 (15)	−0.0037 (12)	−0.0006 (13)	0.0113 (12)
C3_11	0.065 (3)	0.050 (2)	0.048 (2)	0.016 (2)	−0.007 (2)	0.0144 (19)
F4_11	0.0519 (17)	0.0589 (16)	0.0664 (18)	−0.0008 (13)	−0.0088 (14)	0.0226 (14)
F5_11	0.0590 (19)	0.056 (3)	0.059 (3)	0.0146 (16)	0.0051 (18)	0.013 (2)
F6_11	0.079 (2)	0.077 (2)	0.0623 (18)	0.0216 (17)	−0.0015 (16)	0.0374 (16)
C4_11	0.079 (3)	0.049 (2)	0.040 (2)	0.015 (2)	0.015 (2)	0.0195 (18)
F7_11	0.114 (3)	0.0633 (18)	0.0385 (16)	0.0043 (18)	0.0015 (16)	0.0113 (13)
F8_11	0.080 (3)	0.0689 (19)	0.0478 (16)	0.0172 (19)	0.0191 (17)	0.0315 (15)
F9_11	0.112 (3)	0.085 (2)	0.0526 (17)	0.058 (2)	0.0326 (18)	0.0307 (16)
O1_12	0.0509 (19)	0.0398 (16)	0.0370 (15)	0.0054 (13)	0.0071 (13)	0.0157 (13)
C1_12	0.053 (2)	0.0388 (19)	0.042 (2)	0.0064 (17)	0.0078 (17)	0.0209 (16)
C2_12	0.054 (3)	0.042 (2)	0.063 (3)	0.0005 (19)	0.014 (2)	0.021 (2)
F1_12	0.09 (2)	0.092 (17)	0.072 (11)	0.04 (2)	0.024 (16)	0.026 (15)
F2_12	0.050 (16)	0.040 (8)	0.047 (15)	0.005 (9)	0.017 (13)	0.018 (10)
F3_12	0.0503 (16)	0.0486 (14)	0.0553 (15)	−0.0037 (12)	−0.0006 (13)	0.0113 (12)
C3_12	0.065 (3)	0.050 (2)	0.048 (2)	0.016 (2)	−0.007 (2)	0.0144 (19)
F4_12	0.078 (13)	0.08 (3)	0.07 (3)	0.046 (14)	0.013 (16)	0.05 (3)
F5_12	0.13 (3)	0.10 (2)	0.081 (18)	0.00 (2)	−0.02 (2)	−0.032 (17)
F6_12	0.079 (2)	0.077 (2)	0.0623 (18)	0.0216 (17)	−0.0015 (16)	0.0374 (16)
C4_12	0.079 (3)	0.049 (2)	0.040 (2)	0.015 (2)	0.015 (2)	0.0195 (18)
F7_12	0.065 (9)	0.071 (19)	0.054 (18)	−0.008 (11)	−0.011 (9)	0.023 (16)
F8_12	0.10 (2)	0.11 (2)	0.059 (15)	0.05 (2)	0.032 (18)	0.066 (18)
F9_12	0.114 (3)	0.0633 (18)	0.0385 (16)	0.0043 (18)	0.0015 (16)	0.0113 (13)
O1_13	0.085 (2)	0.0540 (18)	0.0460 (17)	−0.0303 (17)	−0.0244 (17)	0.0204 (14)
C1_13	0.045 (2)	0.0338 (18)	0.0398 (19)	−0.0091 (15)	−0.0101 (16)	0.0098 (15)
C2_13	0.065 (3)	0.059 (3)	0.062 (3)	0.014 (2)	−0.004 (2)	0.016 (2)
F1_13	0.112 (3)	0.123 (3)	0.126 (3)	0.073 (3)	−0.005 (3)	0.030 (3)
F2_13	0.118 (3)	0.0450 (15)	0.0594 (17)	−0.0050 (16)	−0.0014 (17)	−0.0018 (13)
F3_13	0.078 (2)	0.104 (3)	0.0575 (17)	−0.0042 (19)	0.0148 (16)	0.0153 (17)
C3_13	0.056 (3)	0.093 (4)	0.081 (4)	0.023 (3)	−0.020 (3)	−0.017 (3)
F4_13	0.106 (3)	0.127 (3)	0.144 (4)	0.076 (3)	−0.019 (3)	−0.030 (3)
F5_13	0.121 (3)	0.095 (3)	0.082 (2)	0.042 (2)	−0.056 (2)	0.0024 (19)
F6_13	0.043 (2)	0.175 (4)	0.137 (4)	−0.023 (2)	0.003 (2)	−0.014 (3)
C4_13	0.113 (4)	0.055 (3)	0.052 (3)	−0.027 (3)	−0.002 (3)	0.020 (2)
F7_13	0.135 (3)	0.104 (3)	0.0564 (19)	−0.054 (2)	0.032 (2)	−0.0073 (18)
F8_13	0.220 (5)	0.0588 (19)	0.070 (2)	−0.053 (3)	0.008 (3)	0.0211 (17)
F9_13	0.157 (4)	0.094 (3)	0.075 (2)	−0.011 (2)	−0.048 (2)	0.048 (2)
O1_20	0.0453 (16)	0.0386 (13)	0.0430 (14)	0.0081 (12)	0.0011 (12)	0.0136 (11)
C1_20	0.045 (2)	0.0396 (19)	0.0414 (19)	0.0154 (16)	0.0035 (16)	0.0166 (15)
C2_20	0.046 (2)	0.074 (3)	0.054 (2)	0.019 (2)	0.0020 (19)	0.035 (2)
F1_20	0.0498 (16)	0.130 (3)	0.0725 (19)	0.0145 (17)	0.0116 (14)	0.064 (2)
F2_20	0.0563 (17)	0.099 (2)	0.0747 (19)	0.0346 (16)	0.0092 (14)	0.0428 (17)
F3_20	0.0518 (17)	0.0694 (18)	0.090 (2)	−0.0049 (14)	−0.0146 (15)	0.0249 (16)
C3_20	0.055 (3)	0.061 (3)	0.051 (2)	0.021 (2)	0.009 (2)	0.031 (2)
F4_20	0.0575 (17)	0.088 (2)	0.0644 (17)	0.0136 (15)	0.0143 (13)	0.0458 (15)
F5_20	0.0676 (18)	0.096 (2)	0.0404 (13)	0.0179 (16)	0.0016 (12)	0.0242 (14)
F6_20	0.091 (2)	0.0799 (19)	0.081 (2)	0.0415 (18)	0.0274 (17)	0.0542 (17)
C4_20	0.079 (3)	0.046 (2)	0.049 (2)	0.021 (2)	0.011 (2)	0.0123 (19)
F7_20	0.090 (2)	0.0552 (16)	0.0706 (19)	−0.0103 (15)	−0.0051 (17)	0.0114 (14)
F8_20	0.124 (3)	0.0650 (19)	0.076 (2)	0.053 (2)	0.013 (2)	0.0066 (16)
F9_20	0.100 (2)	0.0707 (18)	0.0382 (13)	0.0169 (17)	0.0041 (14)	0.0128 (12)
O1_14	0.096 (3)	0.0411 (15)	0.0361 (14)	0.0088 (16)	0.0057 (15)	0.0145 (12)
C1_14	0.049 (2)	0.043 (2)	0.0354 (18)	−0.0007 (16)	−0.0030 (17)	0.0134 (15)
C2_14	0.058 (3)	0.048 (2)	0.060 (3)	−0.003 (2)	0.006 (2)	0.020 (2)
F1_14	0.0489 (17)	0.086 (2)	0.087 (2)	−0.0046 (15)	0.0120 (15)	0.0172 (17)
F2_14	0.129 (3)	0.0501 (16)	0.091 (2)	0.0062 (18)	0.027 (2)	0.0374 (16)
F3_14	0.070 (2)	0.0667 (18)	0.0696 (18)	−0.0162 (15)	−0.0094 (15)	−0.0004 (15)
C3_14	0.060 (3)	0.085 (3)	0.047 (2)	0.019 (3)	−0.004 (2)	0.010 (2)
F4_14	0.0509 (19)	0.148 (4)	0.087 (2)	−0.004 (2)	0.0149 (17)	0.008 (2)
F5_14	0.092 (2)	0.080 (2)	0.0546 (16)	0.0362 (18)	0.0065 (15)	0.0165 (14)
F6_14	0.101 (3)	0.152 (4)	0.0598 (19)	0.071 (3)	−0.0041 (18)	0.028 (2)
C4_14	0.060 (3)	0.059 (3)	0.039 (2)	−0.003 (2)	−0.0012 (19)	0.0169 (18)
F7_14	0.0680 (19)	0.104 (2)	0.0512 (16)	−0.0286 (18)	−0.0089 (14)	0.0016 (16)
F8_14	0.079 (2)	0.0804 (19)	0.0415 (13)	0.0170 (16)	0.0055 (13)	0.0279 (13)
F9_14	0.091 (2)	0.0540 (15)	0.0424 (13)	0.0167 (14)	0.0075 (13)	0.0093 (11)
O1_15	0.0332 (17)	0.0445 (17)	0.0379 (15)	0.0014 (12)	−0.0042 (14)	0.0131 (13)
C1_15	0.0355 (19)	0.0404 (19)	0.0357 (18)	−0.0007 (15)	−0.0100 (15)	0.0068 (15)
C2_15	0.047 (3)	0.054 (3)	0.047 (2)	0.008 (2)	−0.006 (2)	0.0060 (19)
F1_15	0.0470 (17)	0.0456 (15)	0.0713 (19)	0.0095 (13)	−0.0070 (14)	0.0111 (13)
F2_15	0.067 (2)	0.064 (2)	0.062 (2)	0.0087 (16)	−0.0129 (16)	−0.0117 (16)
F3_15	0.061 (2)	0.080 (2)	0.0495 (17)	0.0162 (17)	0.0135 (15)	0.0166 (16)
C3_15	0.043 (2)	0.059 (3)	0.047 (3)	0.003 (2)	−0.011 (2)	0.016 (2)
F4_15	0.0434 (18)	0.0595 (18)	0.069 (2)	0.0115 (14)	−0.0116 (15)	0.0193 (16)
F5_15	0.065 (2)	0.084 (3)	0.0481 (18)	0.007 (2)	−0.0135 (16)	0.0269 (17)
F6_15	0.0480 (17)	0.0630 (18)	0.0667 (19)	−0.0059 (14)	−0.0251 (15)	0.0111 (15)
C4_15	0.039 (2)	0.051 (3)	0.050 (2)	−0.0038 (19)	−0.0083 (18)	0.022 (2)
F7_15	0.0430 (18)	0.080 (2)	0.066 (2)	0.0053 (16)	0.0064 (15)	0.0334 (18)
F8_15	0.060 (2)	0.0461 (16)	0.083 (2)	−0.0131 (14)	−0.0184 (18)	0.0228 (16)
F9_15	0.052 (2)	0.061 (2)	0.051 (2)	−0.0021 (18)	−0.0123 (17)	0.0294 (17)
O1_16	0.0332 (17)	0.0445 (17)	0.0379 (15)	0.0014 (12)	−0.0042 (14)	0.0131 (13)
C1_16	0.0355 (19)	0.0404 (19)	0.0357 (18)	−0.0007 (15)	−0.0100 (15)	0.0068 (15)
C2_16	0.039 (2)	0.051 (3)	0.050 (2)	−0.0038 (19)	−0.0083 (18)	0.022 (2)
F1_16	0.037 (13)	0.077 (12)	0.062 (12)	−0.005 (9)	0.009 (10)	0.017 (10)
F2_16	0.078 (15)	0.056 (11)	0.063 (14)	−0.027 (10)	−0.029 (12)	0.038 (9)
F3_16	0.051 (14)	0.07 (2)	0.07 (2)	−0.005 (12)	−0.012 (12)	0.046 (17)
C3_16	0.047 (3)	0.054 (3)	0.047 (2)	0.008 (2)	−0.006 (2)	0.0060 (19)
F4_16	0.058 (11)	0.073 (14)	0.030 (9)	0.015 (10)	−0.007 (7)	0.006 (8)
F5_16	0.067 (14)	0.032 (9)	0.092 (17)	−0.003 (9)	−0.005 (12)	0.017 (9)
F6_16	0.066 (12)	0.104 (19)	0.047 (13)	−0.013 (11)	−0.020 (10)	−0.014 (11)
C4_16	0.043 (2)	0.059 (3)	0.047 (3)	0.003 (2)	−0.011 (2)	0.016 (2)
F7_16	0.041 (12)	0.10 (2)	0.059 (10)	0.021 (14)	−0.005 (10)	0.052 (11)
F8_16	0.038 (7)	0.099 (16)	0.056 (14)	0.004 (9)	−0.013 (9)	0.045 (12)
F9_16	0.053 (13)	0.058 (8)	0.072 (10)	0.018 (8)	−0.003 (10)	0.026 (7)
O1_17	0.096 (3)	0.0445 (16)	0.0335 (14)	0.0129 (16)	0.0148 (15)	0.0114 (12)
C1_17	0.060 (3)	0.0390 (19)	0.0306 (18)	−0.0003 (17)	0.0061 (17)	0.0052 (15)
C2_17	0.047 (2)	0.058 (2)	0.042 (2)	0.0005 (19)	0.0025 (18)	0.0166 (18)
F1_17	0.0537 (16)	0.0752 (18)	0.0533 (15)	−0.0085 (13)	0.0025 (12)	0.0278 (13)
F2_17	0.0673 (17)	0.0520 (14)	0.0436 (13)	−0.0112 (12)	−0.0068 (12)	0.0033 (11)
F3_17	0.0565 (18)	0.119 (3)	0.0634 (18)	0.0229 (17)	0.0037 (14)	0.0385 (18)
C3_17	0.108 (4)	0.040 (2)	0.036 (2)	−0.001 (2)	0.010 (2)	0.0115 (17)
F4_17	0.129 (3)	0.0611 (17)	0.0530 (16)	−0.0325 (18)	0.0112 (18)	0.0187 (14)
F5_17	0.170 (4)	0.0696 (19)	0.0497 (16)	0.051 (2)	0.023 (2)	0.0293 (15)
F6_17	0.095 (2)	0.0584 (15)	0.0316 (12)	0.0011 (14)	0.0068 (13)	0.0108 (11)
C4_17	0.050 (2)	0.061 (3)	0.041 (2)	−0.0047 (19)	0.0076 (18)	0.0088 (19)
F7_17	0.0599 (18)	0.088 (2)	0.0658 (18)	−0.0223 (16)	−0.0116 (14)	0.0173 (16)
F8_17	0.0584 (17)	0.0795 (19)	0.0524 (15)	0.0075 (14)	0.0186 (13)	0.0154 (14)
F9_17	0.0633 (17)	0.0554 (14)	0.0514 (14)	0.0118 (12)	0.0066 (12)	0.0155 (12)
O1_18	0.0372 (16)	0.0398 (16)	0.0359 (13)	0.0003 (13)	−0.0003 (12)	0.0101 (11)
C1_18	0.038 (2)	0.0448 (19)	0.0366 (18)	0.0013 (15)	0.0016 (15)	0.0105 (15)
C2_18	0.040 (2)	0.052 (2)	0.049 (2)	0.0014 (18)	−0.0016 (18)	0.0134 (19)
F1_18	0.062 (2)	0.080 (2)	0.0447 (15)	0.0215 (16)	−0.0041 (14)	0.0209 (14)
F2_18	0.0444 (17)	0.071 (2)	0.0610 (17)	0.0136 (14)	0.0004 (13)	0.0129 (15)
F3_18	0.0446 (17)	0.0581 (17)	0.091 (2)	−0.0116 (13)	−0.0132 (16)	0.0184 (16)
C3_18	0.058 (3)	0.077 (3)	0.047 (3)	0.017 (2)	0.009 (2)	0.025 (2)
F4_18	0.079 (2)	0.124 (3)	0.0439 (17)	0.027 (2)	−0.0113 (17)	0.0182 (19)
F5_18	0.073 (3)	0.078 (2)	0.078 (2)	0.0210 (19)	0.022 (2)	0.0501 (18)
F6_18	0.080 (3)	0.119 (4)	0.066 (2)	0.040 (2)	0.033 (2)	0.046 (2)
C4_18	0.043 (2)	0.047 (2)	0.064 (3)	0.002 (2)	0.004 (2)	−0.001 (2)
F7_18	0.0419 (18)	0.061 (3)	0.099 (3)	−0.0095 (18)	−0.0021 (17)	−0.014 (2)
F8_18	0.0544 (19)	0.067 (2)	0.076 (2)	0.0076 (15)	0.0008 (16)	−0.0149 (17)
F9_18	0.080 (3)	0.0440 (17)	0.101 (3)	0.0042 (16)	0.014 (2)	0.0278 (18)
O1_19	0.0372 (16)	0.0398 (16)	0.0359 (13)	0.0003 (13)	−0.0003 (12)	0.0101 (11)
C1_19	0.038 (2)	0.0448 (19)	0.0366 (18)	0.0013 (15)	0.0016 (15)	0.0105 (15)
C2_19	0.040 (2)	0.052 (2)	0.049 (2)	0.0014 (18)	−0.0016 (18)	0.0134 (19)
F1_19	0.08 (2)	0.077 (16)	0.09 (3)	0.013 (13)	0.017 (17)	0.055 (16)
F2_19	0.0444 (17)	0.071 (2)	0.0610 (17)	0.0136 (14)	0.0004 (13)	0.0129 (15)
F3_19	0.062 (2)	0.080 (2)	0.0447 (15)	0.0215 (16)	−0.0041 (14)	0.0209 (14)
C3_19	0.058 (3)	0.077 (3)	0.047 (3)	0.017 (2)	0.009 (2)	0.025 (2)
F4_19	0.073 (3)	0.078 (2)	0.078 (2)	0.0210 (19)	0.022 (2)	0.0501 (18)
F5_19	0.09 (2)	0.08 (2)	0.090 (18)	−0.029 (16)	−0.011 (15)	0.027 (14)
F6_19	0.08 (2)	0.08 (2)	0.074 (18)	0.038 (19)	0.035 (17)	0.068 (15)
C4_19	0.043 (2)	0.047 (2)	0.064 (3)	0.002 (2)	0.004 (2)	−0.001 (2)
F7_19	0.09 (2)	0.12 (3)	0.023 (16)	0.03 (2)	−0.023 (15)	−0.019 (15)
F8_19	0.06 (2)	0.10 (3)	0.071 (19)	0.010 (17)	0.012 (15)	−0.022 (15)
F9_19	0.06 (2)	0.024 (14)	0.14 (3)	0.004 (12)	0.037 (19)	−0.011 (14)

### Tetrakis(acetonitrile)bis(nonafluoro-tert-butanolato)titanium(III) tetrakis(nonafluoro-tert-butanolato)aluminate Geometric parameters (Å, º)

**Table:**

Ti1—O1_2^i^	1.818 (10)	C1_8—C3_8	1.546 (12)
Ti1—O1_2	1.818 (10)	C1_8—C2_8	1.551 (12)
Ti1—O1_1	1.897 (8)	C1_8—C4_8	1.554 (12)
Ti1—O1_1^i^	1.897 (8)	C2_8—F1_8	1.323 (13)
Ti1—N5^i^	2.163 (4)	C2_8—F3_8	1.330 (13)
Ti1—N5	2.163 (4)	C2_8—F2_8	1.335 (13)
Ti1—N6^i^	2.170 (4)	C3_8—F6_8	1.320 (13)
Ti1—N6	2.170 (4)	C3_8—F4_8	1.324 (13)
Al1—O1_13	1.696 (3)	C3_8—F5_8	1.327 (13)
Al1—O1_9	1.701 (4)	C4_8—F7_8	1.320 (13)
Al1—O1_17	1.716 (3)	C4_8—F9_8	1.329 (13)
Al1—O1_14	1.737 (3)	C4_8—F8_8	1.335 (13)
Al1—O1_10	1.758 (11)	O1_9—C1_9	1.354 (7)
N1—C1	1.130 (5)	C1_9—C4_9	1.536 (8)
N1—Ti3	2.184 (4)	C1_9—C2_9	1.551 (7)
C1—C2	1.458 (6)	C1_9—C3_9	1.552 (7)
Ti2—O1_19	1.828 (14)	C2_9—F1_9	1.321 (7)
Ti2—O1_19^ii^	1.828 (14)	C2_9—F2_9	1.327 (7)
Ti2—O1_18^ii^	1.867 (3)	C2_9—F3_9	1.340 (8)
Ti2—O1_18	1.867 (3)	C3_9—F5_9	1.327 (7)
Ti2—N7^ii^	2.170 (3)	C3_9—F4_9	1.334 (7)
Ti2—N7	2.170 (3)	C3_9—F6_9	1.336 (7)
Ti2—N8^ii^	2.173 (3)	C4_9—F7_9	1.333 (8)
Ti2—N8	2.173 (3)	C4_9—F8_9	1.334 (8)
Al2—O1_6	1.63 (3)	C4_9—F9_9	1.339 (8)
Al2—O1_16	1.66 (2)	O1_10—C1_10	1.355 (12)
Al2—O1_11	1.714 (4)	C1_10—C4_10	1.550 (12)
Al2—O1_7	1.717 (3)	C1_10—C3_10	1.551 (12)
Al2—O1_15	1.726 (3)	C1_10—C2_10	1.551 (12)
Al2—O1_5	1.740 (5)	C2_10—F2_10	1.329 (13)
Al2—O1_8	1.795 (16)	C2_10—F3_10	1.331 (13)
Al2—O1_12	1.81 (4)	C2_10—F1_10	1.331 (13)
N2—C3	1.137 (5)	C3_10—F4_10	1.331 (13)
N2—Ti3	2.177 (3)	C3_10—F5_10	1.334 (13)
C2—H2A	0.9800	C3_10—F6_10	1.339 (12)
C2—H2B	0.9800	C4_10—F8_10	1.324 (13)
C2—H2C	0.9800	C4_10—F9_10	1.325 (13)
C7—N4	1.136 (5)	C4_10—F7_10	1.336 (13)
C7—C8	1.456 (6)	O1_11—C1_11	1.349 (5)
N4—Ti3	2.182 (3)	C1_11—C3_11	1.549 (6)
C4—C3	1.448 (6)	C1_11—C4_11	1.555 (6)
C4—H4A	0.9800	C1_11—C2_11	1.567 (6)
C4—H4B	0.9800	C2_11—F3_11	1.301 (5)
C4—H4C	0.9800	C2_11—F1_11	1.331 (6)
Ti3—O1_20	1.850 (3)	C2_11—F2_11	1.357 (5)
Ti3—O1_4	1.854 (8)	C3_11—F6_11	1.319 (5)
Ti3—O1_3	1.863 (10)	C3_11—F5_11	1.327 (6)
Ti3—N3	2.177 (4)	C3_11—F4_11	1.346 (6)
N3—C5	1.142 (5)	C4_11—F9_11	1.300 (6)
N5—C9	1.143 (6)	C4_11—F8_11	1.325 (5)
C5—C6	1.448 (6)	C4_11—F7_11	1.371 (6)
N6—C11	1.128 (6)	O1_12—C1_12	1.336 (14)
C6—H6A	0.9800	C1_12—C4_12	1.548 (13)
C6—H6B	0.9800	C1_12—C2_12	1.555 (13)
C6—H6C	0.9800	C1_12—C3_12	1.561 (13)
N7—C13	1.137 (5)	C2_12—F1_12	1.324 (14)
N8—C15	1.137 (5)	C2_12—F3_12	1.334 (14)
C8—H8A	0.9800	C2_12—F2_12	1.340 (14)
C8—H8B	0.9800	C3_12—F4_12	1.322 (14)
C8—H8C	0.9800	C3_12—F5_12	1.324 (14)
C9—C10	1.445 (7)	C3_12—F6_12	1.338 (14)
C10—H10A	0.9800	C4_12—F9_12	1.328 (14)
C10—H10B	0.9800	C4_12—F8_12	1.340 (14)
C10—H10C	0.9800	C4_12—F7_12	1.347 (14)
C11—C12	1.449 (6)	O1_13—C1_13	1.342 (4)
C12—H12A	0.9800	C1_13—C3_13	1.530 (6)
C12—H12B	0.9800	C1_13—C4_13	1.535 (6)
C12—H12C	0.9800	C1_13—C2_13	1.551 (6)
C13—C14	1.460 (6)	C2_13—F1_13	1.319 (6)
C14—H14A	0.9800	C2_13—F3_13	1.324 (6)
C14—H14B	0.9800	C2_13—F2_13	1.345 (6)
C14—H14C	0.9800	C3_13—F5_13	1.312 (7)
C15—C16	1.459 (5)	C3_13—F6_13	1.324 (7)
C16—H16A	0.9800	C3_13—F4_13	1.342 (6)
C16—H16B	0.9800	C4_13—F9_13	1.322 (7)
C16—H16C	0.9800	C4_13—F7_13	1.326 (7)
O1_1—C1_1	1.378 (10)	C4_13—F8_13	1.329 (5)
C1_1—C4_1	1.533 (10)	O1_20—C1_20	1.351 (4)
C1_1—C3_1	1.543 (10)	C1_20—C4_20	1.545 (5)
C1_1—C2_1	1.559 (10)	C1_20—C2_20	1.552 (6)
C2_1—F1_1	1.307 (10)	C1_20—C3_20	1.552 (5)
C2_1—F3_1	1.321 (11)	C2_20—F3_20	1.310 (5)
C2_1—F2_1	1.326 (11)	C2_20—F1_20	1.324 (5)
C3_1—F5_1	1.317 (10)	C2_20—F2_20	1.340 (5)
C3_1—F6_1	1.320 (10)	C3_20—F5_20	1.322 (5)
C3_1—F4_1	1.326 (9)	C3_20—F4_20	1.329 (5)
C4_1—F9_1	1.301 (11)	C3_20—F6_20	1.337 (5)
C4_1—F7_1	1.314 (12)	C4_20—F7_20	1.325 (6)
C4_1—F8_1	1.333 (11)	C4_20—F8_20	1.326 (5)
O1_2—C1_2	1.326 (11)	C4_20—F9_20	1.326 (5)
C1_2—C4_2	1.539 (11)	O1_14—C1_14	1.339 (5)
C1_2—C2_2	1.550 (11)	C1_14—C3_14	1.543 (6)
C1_2—C3_2	1.558 (11)	C1_14—C2_14	1.545 (6)
C2_2—F1_2	1.307 (11)	C1_14—C4_14	1.548 (5)
C2_2—F2_2	1.313 (12)	C2_14—F2_14	1.318 (5)
C2_2—F3_2	1.321 (12)	C2_14—F3_14	1.326 (5)
C3_2—F4_2	1.316 (12)	C2_14—F1_14	1.339 (6)
C3_2—F6_2	1.317 (12)	C3_14—F4_14	1.304 (6)
C3_2—F5_2	1.329 (12)	C3_14—F5_14	1.338 (5)
C4_2—F7_2	1.325 (12)	C3_14—F6_14	1.350 (6)
C4_2—F8_2	1.326 (12)	C4_14—F9_14	1.325 (5)
C4_2—F9_2	1.340 (13)	C4_14—F7_14	1.328 (5)
O1_3—C1_3	1.353 (11)	C4_14—F8_14	1.334 (5)
C1_3—C3_3	1.538 (11)	O1_15—C1_15	1.351 (5)
C1_3—C2_3	1.547 (11)	C1_15—C4_15	1.540 (6)
C1_3—C4_3	1.551 (11)	C1_15—C3_15	1.557 (6)
C2_3—F3_3	1.318 (11)	C1_15—C2_15	1.558 (6)
C2_3—F1_3	1.321 (11)	C2_15—F1_15	1.319 (5)
C2_3—F2_3	1.332 (12)	C2_15—F3_15	1.331 (6)
C3_3—F5_3	1.323 (12)	C2_15—F2_15	1.339 (5)
C3_3—F4_3	1.324 (12)	C3_15—F4_15	1.321 (6)
C3_3—F6_3	1.343 (12)	C3_15—F6_15	1.330 (5)
C4_3—F7_3	1.317 (12)	C3_15—F5_15	1.344 (6)
C4_3—F8_3	1.322 (12)	C4_15—F7_15	1.324 (6)
C4_3—F9_3	1.338 (12)	C4_15—F9_15	1.335 (5)
O1_4—C1_4	1.354 (9)	C4_15—F8_15	1.337 (5)
C1_4—C4_4	1.530 (10)	O1_16—C1_16	1.353 (13)
C1_4—C3_4	1.548 (9)	C1_16—C2_16	1.548 (13)
C1_4—C2_4	1.568 (10)	C1_16—C4_16	1.548 (13)
C2_4—F3_4	1.317 (10)	C1_16—C3_16	1.550 (13)
C2_4—F1_4	1.326 (10)	C2_16—F1_16	1.327 (14)
C2_4—F2_4	1.338 (11)	C2_16—F2_16	1.327 (14)
C3_4—F5_4	1.317 (9)	C2_16—F3_16	1.330 (14)
C3_4—F6_4	1.331 (10)	C3_16—F6_16	1.328 (13)
C3_4—F4_4	1.338 (9)	C3_16—F5_16	1.328 (13)
C4_4—F9_4	1.323 (11)	C3_16—F4_16	1.330 (13)
C4_4—F7_4	1.329 (11)	C4_16—F9_16	1.326 (13)
C4_4—F8_4	1.349 (10)	C4_16—F7_16	1.328 (13)
O1_5—C1_5	1.358 (6)	C4_16—F8_16	1.329 (13)
C1_5—C3_5	1.541 (6)	O1_17—C1_17	1.352 (4)
C1_5—C4_5	1.542 (6)	C1_17—C2_17	1.544 (5)
C1_5—C2_5	1.552 (7)	C1_17—C4_17	1.545 (6)
C2_5—F3_5	1.333 (7)	C1_17—C3_17	1.550 (5)
C2_5—F1_5	1.334 (7)	C2_17—F1_17	1.330 (5)
C2_5—F2_5	1.338 (7)	C2_17—F2_17	1.334 (5)
C3_5—F4_5	1.325 (6)	C2_17—F3_17	1.335 (5)
C3_5—F5_5	1.341 (6)	C3_17—F5_17	1.305 (6)
C3_5—F6_5	1.346 (6)	C3_17—F4_17	1.337 (6)
C4_5—F9_5	1.328 (6)	C3_17—F6_17	1.339 (5)
C4_5—F8_5	1.340 (7)	C4_17—F7_17	1.318 (5)
C4_5—F7_5	1.341 (7)	C4_17—F9_17	1.333 (5)
O1_6—C1_6	1.347 (13)	C4_17—F8_17	1.343 (5)
C1_6—C3_6	1.547 (12)	O1_18—C1_18	1.351 (5)
C1_6—C4_6	1.548 (13)	C1_18—C3_18	1.544 (6)
C1_6—C2_6	1.553 (13)	C1_18—C4_18	1.555 (6)
C2_6—F1_6	1.329 (13)	C1_18—C2_18	1.560 (6)
C2_6—F2_6	1.331 (14)	C2_18—F2_18	1.324 (5)
C2_6—F3_6	1.331 (13)	C2_18—F3_18	1.328 (5)
C3_6—F4_6	1.325 (13)	C2_18—F1_18	1.333 (6)
C3_6—F5_6	1.329 (13)	C3_18—F4_18	1.329 (6)
C3_6—F6_6	1.329 (13)	C3_18—F5_18	1.342 (6)
C4_6—F8_6	1.323 (13)	C3_18—F6_18	1.345 (6)
C4_6—F7_6	1.326 (13)	C4_18—F9_18	1.321 (6)
C4_6—F9_6	1.327 (13)	C4_18—F7_18	1.327 (6)
O1_7—C1_7	1.358 (5)	C4_18—F8_18	1.333 (6)
C1_7—C4_7	1.541 (7)	O1_19—C1_19	1.340 (14)
C1_7—C3_7	1.545 (6)	C1_19—C2_19	1.543 (13)
C1_7—C2_7	1.562 (8)	C1_19—C3_19	1.546 (13)
C2_7—F1_7	1.319 (7)	C1_19—C4_19	1.548 (13)
C2_7—F3_7	1.333 (7)	C2_19—F3_19	1.326 (14)
C2_7—F2_7	1.344 (7)	C2_19—F2_19	1.328 (14)
C3_7—F5_7	1.311 (6)	C2_19—F1_19	1.329 (14)
C3_7—F4_7	1.335 (7)	C3_19—F5_19	1.323 (14)
C3_7—F6_7	1.342 (6)	C3_19—F6_19	1.327 (14)
C4_7—F7_7	1.324 (7)	C3_19—F4_19	1.331 (14)
C4_7—F8_7	1.332 (6)	C4_19—F8_19	1.325 (14)
C4_7—F9_7	1.336 (7)	C4_19—F7_19	1.327 (14)
O1_8—C1_8	1.351 (13)	C4_19—F9_19	1.327 (14)
			
O1_2^i^—Ti1—O1_2	180.0 (15)	C3_8—C1_8—C2_8	108.8 (12)
O1_1—Ti1—O1_1^i^	180.0	O1_8—C1_8—C4_8	109.7 (12)
O1_1—Ti1—N5^i^	92.4 (8)	C3_8—C1_8—C4_8	109.7 (12)
O1_1^i^—Ti1—N5^i^	87.6 (8)	C2_8—C1_8—C4_8	108.4 (11)
O1_2^i^—Ti1—N5	89.2 (11)	F1_8—C2_8—F3_8	105.9 (15)
O1_2—Ti1—N5	90.8 (11)	F1_8—C2_8—F2_8	109.4 (17)
O1_1—Ti1—N5	87.6 (8)	F3_8—C2_8—F2_8	107.1 (16)
O1_1^i^—Ti1—N5	92.4 (8)	F1_8—C2_8—C1_8	112.0 (14)
N5^i^—Ti1—N5	180.0	F3_8—C2_8—C1_8	111.9 (13)
O1_1—Ti1—N6^i^	90.9 (8)	F2_8—C2_8—C1_8	110.3 (13)
O1_1^i^—Ti1—N6^i^	89.1 (8)	F6_8—C3_8—F4_8	108.9 (17)
N5^i^—Ti1—N6^i^	87.75 (14)	F6_8—C3_8—F5_8	107.0 (16)
N5—Ti1—N6^i^	92.25 (14)	F4_8—C3_8—F5_8	104.3 (17)
O1_2^i^—Ti1—N6	88.1 (11)	F6_8—C3_8—C1_8	114.2 (14)
O1_2—Ti1—N6	91.9 (11)	F4_8—C3_8—C1_8	110.8 (13)
O1_1—Ti1—N6	89.1 (8)	F5_8—C3_8—C1_8	111.1 (13)
O1_1^i^—Ti1—N6	90.9 (8)	F7_8—C4_8—F9_8	110.7 (17)
N5^i^—Ti1—N6	92.25 (14)	F7_8—C4_8—F8_8	108.0 (16)
N5—Ti1—N6	87.75 (14)	F9_8—C4_8—F8_8	108.1 (17)
N6^i^—Ti1—N6	180.0	F7_8—C4_8—C1_8	111.0 (14)
O1_13—Al1—O1_9	114.1 (2)	F9_8—C4_8—C1_8	109.2 (15)
O1_13—Al1—O1_17	109.84 (17)	F8_8—C4_8—C1_8	109.9 (13)
O1_9—Al1—O1_17	110.8 (2)	C1_9—O1_9—Al1	151.0 (4)
O1_13—Al1—O1_14	111.07 (17)	O1_9—C1_9—C4_9	112.6 (5)
O1_9—Al1—O1_14	104.1 (2)	O1_9—C1_9—C2_9	108.6 (5)
O1_17—Al1—O1_14	106.62 (16)	C4_9—C1_9—C2_9	109.5 (5)
O1_13—Al1—O1_10	87.6 (4)	O1_9—C1_9—C3_9	106.9 (4)
O1_17—Al1—O1_10	115.5 (4)	C4_9—C1_9—C3_9	110.1 (5)
O1_14—Al1—O1_10	124.2 (5)	C2_9—C1_9—C3_9	109.1 (5)
C1—N1—Ti3	170.9 (3)	F1_9—C2_9—F2_9	107.8 (6)
N1—C1—C2	178.2 (4)	F1_9—C2_9—F3_9	107.5 (8)
O1_19—Ti2—O1_19^ii^	180.0	F2_9—C2_9—F3_9	108.2 (7)
O1_18^ii^—Ti2—O1_18	180.0	F1_9—C2_9—C1_9	109.8 (6)
O1_18^ii^—Ti2—N7^ii^	89.26 (16)	F2_9—C2_9—C1_9	112.0 (6)
O1_18—Ti2—N7^ii^	90.74 (16)	F3_9—C2_9—C1_9	111.4 (7)
O1_19—Ti2—N7	94.2 (17)	F5_9—C3_9—F4_9	106.3 (6)
O1_19^ii^—Ti2—N7	85.8 (17)	F5_9—C3_9—F6_9	107.8 (6)
O1_18^ii^—Ti2—N7	90.74 (16)	F4_9—C3_9—F6_9	107.9 (6)
O1_18—Ti2—N7	89.26 (16)	F5_9—C3_9—C1_9	111.2 (5)
N7^ii^—Ti2—N7	180.00 (6)	F4_9—C3_9—C1_9	111.1 (6)
O1_18^ii^—Ti2—N8^ii^	90.82 (16)	F6_9—C3_9—C1_9	112.3 (5)
O1_18—Ti2—N8^ii^	89.18 (16)	F7_9—C4_9—F8_9	108.1 (6)
N7^ii^—Ti2—N8^ii^	90.05 (12)	F7_9—C4_9—F9_9	107.3 (6)
N7—Ti2—N8^ii^	89.95 (12)	F8_9—C4_9—F9_9	109.1 (6)
O1_19—Ti2—N8	87.0 (17)	F7_9—C4_9—C1_9	109.6 (6)
O1_19^ii^—Ti2—N8	93.0 (17)	F8_9—C4_9—C1_9	112.4 (6)
O1_18^ii^—Ti2—N8	89.18 (16)	F9_9—C4_9—C1_9	110.1 (5)
O1_18—Ti2—N8	90.82 (16)	C1_10—O1_10—Al1	153.2 (11)
N7^ii^—Ti2—N8	89.95 (12)	O1_10—C1_10—C4_10	108.4 (12)
N7—Ti2—N8	90.05 (12)	O1_10—C1_10—C3_10	111.6 (11)
N8^ii^—Ti2—N8	180.0	C4_10—C1_10—C3_10	108.3 (11)
O1_6—Al2—O1_16	117.7 (17)	O1_10—C1_10—C2_10	110.6 (11)
O1_11—Al2—O1_7	115.3 (2)	C4_10—C1_10—C2_10	110.3 (11)
O1_11—Al2—O1_15	107.4 (2)	C3_10—C1_10—C2_10	107.6 (10)
O1_7—Al2—O1_15	106.50 (16)	F2_10—C2_10—F3_10	107.2 (15)
O1_11—Al2—O1_5	107.8 (3)	F2_10—C2_10—F1_10	107.4 (14)
O1_7—Al2—O1_5	107.3 (3)	F3_10—C2_10—F1_10	109.0 (15)
O1_15—Al2—O1_5	112.7 (3)	F2_10—C2_10—C1_10	112.5 (14)
O1_6—Al2—O1_8	109.7 (16)	F3_10—C2_10—C1_10	110.5 (13)
O1_16—Al2—O1_8	103.5 (10)	F1_10—C2_10—C1_10	110.0 (12)
O1_6—Al2—O1_12	102 (2)	F4_10—C3_10—F5_10	108.1 (16)
O1_16—Al2—O1_12	120.9 (18)	F4_10—C3_10—F6_10	107.4 (13)
O1_8—Al2—O1_12	101.9 (17)	F5_10—C3_10—F6_10	108.9 (14)
C3—N2—Ti3	173.7 (3)	F4_10—C3_10—C1_10	110.3 (11)
C1—C2—H2A	109.5	F5_10—C3_10—C1_10	109.9 (14)
C1—C2—H2B	109.5	F6_10—C3_10—C1_10	112.0 (13)
H2A—C2—H2B	109.5	F8_10—C4_10—F9_10	107.8 (14)
C1—C2—H2C	109.5	F8_10—C4_10—F7_10	108.3 (19)
H2A—C2—H2C	109.5	F9_10—C4_10—F7_10	106.5 (19)
H2B—C2—H2C	109.5	F8_10—C4_10—C1_10	113.0 (14)
N4—C7—C8	177.5 (5)	F9_10—C4_10—C1_10	111.6 (13)
C7—N4—Ti3	166.4 (4)	F7_10—C4_10—C1_10	109.5 (18)
C3—C4—H4A	109.5	C1_11—O1_11—Al2	150.2 (4)
C3—C4—H4B	109.5	O1_11—C1_11—C3_11	109.5 (4)
H4A—C4—H4B	109.5	O1_11—C1_11—C4_11	111.1 (4)
C3—C4—H4C	109.5	C3_11—C1_11—C4_11	111.3 (4)
H4A—C4—H4C	109.5	O1_11—C1_11—C2_11	107.9 (4)
H4B—C4—H4C	109.5	C3_11—C1_11—C2_11	108.9 (4)
O1_20—Ti3—O1_4	178.0 (6)	C4_11—C1_11—C2_11	108.0 (4)
O1_20—Ti3—O1_3	178.0 (9)	F3_11—C2_11—F1_11	109.2 (4)
O1_20—Ti3—N3	88.96 (13)	F3_11—C2_11—F2_11	107.0 (4)
O1_4—Ti3—N3	91.1 (5)	F1_11—C2_11—F2_11	106.8 (4)
O1_3—Ti3—N3	93.0 (8)	F3_11—C2_11—C1_11	112.1 (4)
O1_20—Ti3—N2	91.27 (13)	F1_11—C2_11—C1_11	110.9 (4)
O1_4—Ti3—N2	90.7 (6)	F2_11—C2_11—C1_11	110.6 (4)
O1_3—Ti3—N2	88.4 (9)	F6_11—C3_11—F5_11	108.4 (4)
N3—Ti3—N2	91.98 (13)	F6_11—C3_11—F4_11	106.9 (4)
O1_20—Ti3—N4	92.24 (13)	F5_11—C3_11—F4_11	105.2 (4)
O1_4—Ti3—N4	85.8 (6)	F6_11—C3_11—C1_11	113.9 (4)
O1_3—Ti3—N4	88.1 (9)	F5_11—C3_11—C1_11	111.4 (4)
N3—Ti3—N4	87.70 (13)	F4_11—C3_11—C1_11	110.6 (4)
N2—Ti3—N4	176.48 (13)	F9_11—C4_11—F8_11	108.7 (4)
O1_20—Ti3—N1	90.67 (13)	F9_11—C4_11—F7_11	107.9 (4)
O1_4—Ti3—N1	89.4 (5)	F8_11—C4_11—F7_11	106.6 (4)
O1_3—Ti3—N1	87.4 (8)	F9_11—C4_11—C1_11	112.4 (4)
N3—Ti3—N1	178.32 (12)	F8_11—C4_11—C1_11	113.1 (4)
N2—Ti3—N1	86.39 (13)	F7_11—C4_11—C1_11	107.9 (4)
N4—Ti3—N1	93.95 (13)	C1_12—O1_12—Al2	144 (3)
C5—N3—Ti3	168.2 (3)	O1_12—C1_12—C4_12	112 (2)
N2—C3—C4	178.7 (5)	O1_12—C1_12—C2_12	111.3 (19)
C9—N5—Ti1	169.2 (4)	C4_12—C1_12—C2_12	111.0 (14)
N3—C5—C6	178.1 (4)	O1_12—C1_12—C3_12	112 (2)
C11—N6—Ti1	175.2 (4)	C4_12—C1_12—C3_12	105.5 (13)
C5—C6—H6A	109.5	C2_12—C1_12—C3_12	104.6 (14)
C5—C6—H6B	109.5	F1_12—C2_12—F3_12	110.9 (19)
H6A—C6—H6B	109.5	F1_12—C2_12—F2_12	112 (2)
C5—C6—H6C	109.5	F3_12—C2_12—F2_12	106.5 (18)
H6A—C6—H6C	109.5	F1_12—C2_12—C1_12	108.1 (17)
H6B—C6—H6C	109.5	F3_12—C2_12—C1_12	109.1 (16)
C13—N7—Ti2	174.3 (4)	F2_12—C2_12—C1_12	110.0 (16)
C15—N8—Ti2	174.3 (3)	F4_12—C3_12—F5_12	110 (2)
C7—C8—H8A	109.5	F4_12—C3_12—F6_12	108 (2)
C7—C8—H8B	109.5	F5_12—C3_12—F6_12	112 (2)
H8A—C8—H8B	109.5	F4_12—C3_12—C1_12	110.5 (19)
C7—C8—H8C	109.5	F5_12—C3_12—C1_12	108.2 (18)
H8A—C8—H8C	109.5	F6_12—C3_12—C1_12	108.3 (16)
H8B—C8—H8C	109.5	F9_12—C4_12—F8_12	104.5 (19)
N5—C9—C10	177.8 (5)	F9_12—C4_12—F7_12	117 (2)
C9—C10—H10A	109.5	F8_12—C4_12—F7_12	106.3 (19)
C9—C10—H10B	109.5	F9_12—C4_12—C1_12	112.2 (17)
H10A—C10—H10B	109.5	F8_12—C4_12—C1_12	111.4 (19)
C9—C10—H10C	109.5	F7_12—C4_12—C1_12	105.4 (15)
H10A—C10—H10C	109.5	C1_13—O1_13—Al1	153.2 (3)
H10B—C10—H10C	109.5	O1_13—C1_13—C3_13	110.7 (4)
N6—C11—C12	179.1 (7)	O1_13—C1_13—C4_13	110.4 (3)
C11—C12—H12A	109.5	C3_13—C1_13—C4_13	110.8 (4)
C11—C12—H12B	109.5	O1_13—C1_13—C2_13	107.3 (4)
H12A—C12—H12B	109.5	C3_13—C1_13—C2_13	108.4 (3)
C11—C12—H12C	109.5	C4_13—C1_13—C2_13	109.3 (4)
H12A—C12—H12C	109.5	F1_13—C2_13—F3_13	109.0 (5)
H12B—C12—H12C	109.5	F1_13—C2_13—F2_13	107.7 (4)
N7—C13—C14	179.3 (5)	F3_13—C2_13—F2_13	106.8 (4)
C13—C14—H14A	109.5	F1_13—C2_13—C1_13	110.6 (4)
C13—C14—H14B	109.5	F3_13—C2_13—C1_13	111.3 (4)
H14A—C14—H14B	109.5	F2_13—C2_13—C1_13	111.3 (4)
C13—C14—H14C	109.5	F5_13—C3_13—F6_13	107.5 (5)
H14A—C14—H14C	109.5	F5_13—C3_13—F4_13	107.3 (6)
H14B—C14—H14C	109.5	F6_13—C3_13—F4_13	109.8 (6)
N8—C15—C16	179.5 (5)	F5_13—C3_13—C1_13	111.7 (5)
C15—C16—H16A	109.5	F6_13—C3_13—C1_13	111.9 (5)
C15—C16—H16B	109.5	F4_13—C3_13—C1_13	108.5 (4)
H16A—C16—H16B	109.5	F9_13—C4_13—F7_13	106.1 (5)
C15—C16—H16C	109.5	F9_13—C4_13—F8_13	107.2 (5)
H16A—C16—H16C	109.5	F7_13—C4_13—F8_13	108.9 (5)
H16B—C16—H16C	109.5	F9_13—C4_13—C1_13	110.7 (4)
C1_1—O1_1—Ti1	179.4 (18)	F7_13—C4_13—C1_13	111.4 (5)
O1_1—C1_1—C4_1	109.1 (12)	F8_13—C4_13—C1_13	112.3 (4)
O1_1—C1_1—C3_1	110.6 (11)	C1_20—O1_20—Ti3	174.3 (3)
C4_1—C1_1—C3_1	107.9 (7)	O1_20—C1_20—C4_20	109.3 (3)
O1_1—C1_1—C2_1	110.0 (11)	O1_20—C1_20—C2_20	109.1 (3)
C4_1—C1_1—C2_1	111.0 (9)	C4_20—C1_20—C2_20	110.1 (4)
C3_1—C1_1—C2_1	108.2 (7)	O1_20—C1_20—C3_20	109.5 (3)
F1_1—C2_1—F3_1	110.1 (11)	C4_20—C1_20—C3_20	109.4 (3)
F1_1—C2_1—F2_1	108.9 (10)	C2_20—C1_20—C3_20	109.4 (3)
F3_1—C2_1—F2_1	106.4 (11)	F3_20—C2_20—F1_20	108.2 (4)
F1_1—C2_1—C1_1	108.6 (9)	F3_20—C2_20—F2_20	107.4 (4)
F3_1—C2_1—C1_1	109.1 (8)	F1_20—C2_20—F2_20	107.1 (4)
F2_1—C2_1—C1_1	113.6 (8)	F3_20—C2_20—C1_20	110.8 (4)
F5_1—C3_1—F6_1	106.8 (9)	F1_20—C2_20—C1_20	111.1 (3)
F5_1—C3_1—F4_1	109.0 (10)	F2_20—C2_20—C1_20	112.1 (4)
F6_1—C3_1—F4_1	107.1 (8)	F5_20—C3_20—F4_20	107.5 (4)
F5_1—C3_1—C1_1	111.2 (7)	F5_20—C3_20—F6_20	108.2 (4)
F6_1—C3_1—C1_1	112.3 (8)	F4_20—C3_20—F6_20	106.6 (4)
F4_1—C3_1—C1_1	110.2 (8)	F5_20—C3_20—C1_20	110.9 (4)
F9_1—C4_1—F7_1	112.1 (13)	F4_20—C3_20—C1_20	110.7 (3)
F9_1—C4_1—F8_1	107.0 (10)	F6_20—C3_20—C1_20	112.8 (4)
F7_1—C4_1—F8_1	98.0 (10)	F7_20—C4_20—F8_20	108.2 (4)
F9_1—C4_1—C1_1	111.6 (9)	F7_20—C4_20—F9_20	106.9 (4)
F7_1—C4_1—C1_1	110.0 (9)	F8_20—C4_20—F9_20	107.2 (4)
F8_1—C4_1—C1_1	117.4 (9)	F7_20—C4_20—C1_20	111.4 (4)
C1_2—O1_2—Ti1	177 (3)	F8_20—C4_20—C1_20	112.1 (4)
O1_2—C1_2—C4_2	110.1 (15)	F9_20—C4_20—C1_20	110.7 (3)
O1_2—C1_2—C2_2	108.4 (15)	C1_14—O1_14—Al1	147.6 (3)
C4_2—C1_2—C2_2	110.7 (9)	O1_14—C1_14—C3_14	107.0 (4)
O1_2—C1_2—C3_2	109.0 (15)	O1_14—C1_14—C2_14	110.6 (3)
C4_2—C1_2—C3_2	111.8 (10)	C3_14—C1_14—C2_14	108.3 (4)
C2_2—C1_2—C3_2	106.6 (9)	O1_14—C1_14—C4_14	111.7 (3)
F1_2—C2_2—F2_2	116.3 (13)	C3_14—C1_14—C4_14	110.0 (3)
F1_2—C2_2—F3_2	109.9 (13)	C2_14—C1_14—C4_14	109.3 (3)
F2_2—C2_2—F3_2	102.3 (13)	F2_14—C2_14—F3_14	108.2 (4)
F1_2—C2_2—C1_2	111.5 (10)	F2_14—C2_14—F1_14	107.5 (4)
F2_2—C2_2—C1_2	107.6 (11)	F3_14—C2_14—F1_14	106.8 (4)
F3_2—C2_2—C1_2	108.6 (10)	F2_14—C2_14—C1_14	113.1 (4)
F4_2—C3_2—F6_2	114.5 (16)	F3_14—C2_14—C1_14	111.4 (4)
F4_2—C3_2—F5_2	111.6 (14)	F1_14—C2_14—C1_14	109.7 (4)
F6_2—C3_2—F5_2	107.5 (15)	F4_14—C3_14—F5_14	107.1 (4)
F4_2—C3_2—C1_2	106.4 (11)	F4_14—C3_14—F6_14	106.7 (4)
F6_2—C3_2—C1_2	108.2 (13)	F5_14—C3_14—F6_14	106.8 (4)
F5_2—C3_2—C1_2	108.4 (10)	F4_14—C3_14—C1_14	112.7 (5)
F7_2—C4_2—F8_2	107.9 (15)	F5_14—C3_14—C1_14	111.0 (4)
F7_2—C4_2—F9_2	109.6 (14)	F6_14—C3_14—C1_14	112.2 (4)
F8_2—C4_2—F9_2	113.7 (15)	F9_14—C4_14—F7_14	107.5 (4)
F7_2—C4_2—C1_2	109.5 (11)	F9_14—C4_14—F8_14	107.5 (4)
F8_2—C4_2—C1_2	109.6 (12)	F7_14—C4_14—F8_14	107.5 (4)
F9_2—C4_2—C1_2	106.6 (10)	F9_14—C4_14—C1_14	110.2 (3)
C1_3—O1_3—Ti3	169 (2)	F7_14—C4_14—C1_14	110.7 (4)
O1_3—C1_3—C3_3	110.1 (13)	F8_14—C4_14—C1_14	113.2 (4)
O1_3—C1_3—C2_3	111.5 (14)	C1_15—O1_15—Al2	147.7 (3)
C3_3—C1_3—C2_3	108.9 (9)	O1_15—C1_15—C4_15	110.8 (3)
O1_3—C1_3—C4_3	107.4 (13)	O1_15—C1_15—C3_15	106.8 (4)
C3_3—C1_3—C4_3	111.4 (10)	C4_15—C1_15—C3_15	110.2 (4)
C2_3—C1_3—C4_3	107.5 (9)	O1_15—C1_15—C2_15	110.4 (4)
F3_3—C2_3—F1_3	105.8 (11)	C4_15—C1_15—C2_15	110.0 (4)
F3_3—C2_3—F2_3	107.1 (14)	C3_15—C1_15—C2_15	108.6 (4)
F1_3—C2_3—F2_3	108.0 (14)	F1_15—C2_15—F3_15	107.2 (4)
F3_3—C2_3—C1_3	111.9 (10)	F1_15—C2_15—F2_15	107.3 (4)
F1_3—C2_3—C1_3	110.7 (9)	F3_15—C2_15—F2_15	107.5 (4)
F2_3—C2_3—C1_3	112.9 (13)	F1_15—C2_15—C1_15	111.3 (4)
F5_3—C3_3—F4_3	110.2 (14)	F3_15—C2_15—C1_15	110.9 (4)
F5_3—C3_3—F6_3	106.4 (15)	F2_15—C2_15—C1_15	112.4 (4)
F4_3—C3_3—F6_3	108.9 (16)	F4_15—C3_15—F6_15	108.1 (4)
F5_3—C3_3—C1_3	110.8 (10)	F4_15—C3_15—F5_15	107.4 (4)
F4_3—C3_3—C1_3	109.6 (11)	F6_15—C3_15—F5_15	107.2 (4)
F6_3—C3_3—C1_3	110.8 (15)	F4_15—C3_15—C1_15	111.2 (4)
F7_3—C4_3—F8_3	110.1 (15)	F6_15—C3_15—C1_15	112.4 (4)
F7_3—C4_3—F9_3	105.9 (13)	F5_15—C3_15—C1_15	110.3 (4)
F8_3—C4_3—F9_3	106.9 (13)	F7_15—C4_15—F9_15	107.4 (5)
F7_3—C4_3—C1_3	108.7 (11)	F7_15—C4_15—F8_15	108.1 (4)
F8_3—C4_3—C1_3	114.1 (12)	F9_15—C4_15—F8_15	107.5 (4)
F9_3—C4_3—C1_3	110.7 (10)	F7_15—C4_15—C1_15	111.4 (4)
C1_4—O1_4—Ti3	170.7 (12)	F9_15—C4_15—C1_15	110.2 (4)
O1_4—C1_4—C4_4	108.5 (9)	F8_15—C4_15—C1_15	112.0 (4)
O1_4—C1_4—C3_4	109.6 (10)	C1_16—O1_16—Al2	147.2 (19)
C4_4—C1_4—C3_4	110.8 (7)	O1_16—C1_16—C2_16	109.7 (14)
O1_4—C1_4—C2_4	109.7 (10)	O1_16—C1_16—C4_16	109.5 (14)
C4_4—C1_4—C2_4	110.1 (7)	C2_16—C1_16—C4_16	109.8 (12)
C3_4—C1_4—C2_4	108.2 (7)	O1_16—C1_16—C3_16	109.0 (14)
F3_4—C2_4—F1_4	108.9 (9)	C2_16—C1_16—C3_16	109.2 (12)
F3_4—C2_4—F2_4	112.1 (11)	C4_16—C1_16—C3_16	109.5 (12)
F1_4—C2_4—F2_4	107.1 (11)	F1_16—C2_16—F2_16	107.1 (18)
F3_4—C2_4—C1_4	110.8 (8)	F1_16—C2_16—F3_16	109 (2)
F1_4—C2_4—C1_4	109.0 (8)	F2_16—C2_16—F3_16	106.9 (19)
F2_4—C2_4—C1_4	108.9 (10)	F1_16—C2_16—C1_16	111.9 (15)
F5_4—C3_4—F6_4	107.6 (10)	F2_16—C2_16—C1_16	111.1 (15)
F5_4—C3_4—F4_4	107.3 (9)	F3_16—C2_16—C1_16	110.8 (19)
F6_4—C3_4—F4_4	108.2 (10)	F6_16—C3_16—F5_16	107.1 (17)
F5_4—C3_4—C1_4	110.5 (8)	F6_16—C3_16—F4_16	107.1 (17)
F6_4—C3_4—C1_4	113.2 (10)	F5_16—C3_16—F4_16	108.6 (16)
F4_4—C3_4—C1_4	110.0 (7)	F6_16—C3_16—C1_16	113.6 (15)
F9_4—C4_4—F7_4	109.6 (11)	F5_16—C3_16—C1_16	110.4 (14)
F9_4—C4_4—F8_4	106.3 (11)	F4_16—C3_16—C1_16	109.8 (14)
F7_4—C4_4—F8_4	106.8 (11)	F9_16—C4_16—F7_16	107.3 (17)
F9_4—C4_4—C1_4	110.7 (8)	F9_16—C4_16—F8_16	106.5 (17)
F7_4—C4_4—C1_4	110.9 (9)	F7_16—C4_16—F8_16	109.3 (17)
F8_4—C4_4—C1_4	112.3 (11)	F9_16—C4_16—C1_16	110.8 (14)
C1_5—O1_5—Al2	147.3 (6)	F7_16—C4_16—C1_16	109.6 (14)
O1_5—C1_5—C3_5	111.1 (5)	F8_16—C4_16—C1_16	113.0 (14)
O1_5—C1_5—C4_5	107.8 (5)	C1_17—O1_17—Al1	147.8 (3)
C3_5—C1_5—C4_5	109.8 (4)	O1_17—C1_17—C2_17	112.0 (4)
O1_5—C1_5—C2_5	111.1 (5)	O1_17—C1_17—C4_17	109.6 (3)
C3_5—C1_5—C2_5	108.3 (4)	C2_17—C1_17—C4_17	108.7 (3)
C4_5—C1_5—C2_5	108.8 (4)	O1_17—C1_17—C3_17	107.8 (3)
F3_5—C2_5—F1_5	106.9 (5)	C2_17—C1_17—C3_17	109.3 (3)
F3_5—C2_5—F2_5	107.5 (5)	C4_17—C1_17—C3_17	109.4 (4)
F1_5—C2_5—F2_5	107.6 (5)	F1_17—C2_17—F2_17	107.7 (4)
F3_5—C2_5—C1_5	110.5 (5)	F1_17—C2_17—F3_17	107.0 (4)
F1_5—C2_5—C1_5	111.1 (5)	F2_17—C2_17—F3_17	107.5 (3)
F2_5—C2_5—C1_5	113.0 (5)	F1_17—C2_17—C1_17	110.7 (3)
F4_5—C3_5—F5_5	107.7 (5)	F2_17—C2_17—C1_17	113.1 (4)
F4_5—C3_5—F6_5	106.6 (4)	F3_17—C2_17—C1_17	110.6 (4)
F5_5—C3_5—F6_5	106.6 (4)	F5_17—C3_17—F4_17	107.5 (4)
F4_5—C3_5—C1_5	111.0 (4)	F5_17—C3_17—F6_17	107.8 (4)
F5_5—C3_5—C1_5	111.6 (4)	F4_17—C3_17—F6_17	107.0 (4)
F6_5—C3_5—C1_5	113.0 (4)	F5_17—C3_17—C1_17	111.3 (4)
F9_5—C4_5—F8_5	107.7 (5)	F4_17—C3_17—C1_17	110.4 (4)
F9_5—C4_5—F7_5	106.5 (5)	F6_17—C3_17—C1_17	112.6 (3)
F8_5—C4_5—F7_5	107.1 (5)	F7_17—C4_17—F9_17	106.8 (4)
F9_5—C4_5—C1_5	111.9 (4)	F7_17—C4_17—F8_17	107.2 (4)
F8_5—C4_5—C1_5	112.8 (5)	F9_17—C4_17—F8_17	106.6 (4)
F7_5—C4_5—C1_5	110.4 (5)	F7_17—C4_17—C1_17	112.1 (4)
C1_6—O1_6—Al2	154 (3)	F9_17—C4_17—C1_17	111.6 (4)
O1_6—C1_6—C3_6	111.2 (18)	F8_17—C4_17—C1_17	112.2 (4)
O1_6—C1_6—C4_6	110.1 (18)	C1_18—O1_18—Ti2	175.1 (3)
C3_6—C1_6—C4_6	108.8 (12)	O1_18—C1_18—C3_18	109.8 (4)
O1_6—C1_6—C2_6	109.0 (18)	O1_18—C1_18—C4_18	109.3 (4)
C3_6—C1_6—C2_6	107.7 (11)	C3_18—C1_18—C4_18	110.5 (4)
C4_6—C1_6—C2_6	110.0 (12)	O1_18—C1_18—C2_18	109.2 (3)
F1_6—C2_6—F2_6	103.7 (16)	C3_18—C1_18—C2_18	109.0 (4)
F1_6—C2_6—F3_6	109.0 (16)	C4_18—C1_18—C2_18	109.0 (4)
F2_6—C2_6—F3_6	111.7 (17)	F2_18—C2_18—F3_18	108.3 (4)
F1_6—C2_6—C1_6	109.0 (13)	F2_18—C2_18—F1_18	107.5 (4)
F2_6—C2_6—C1_6	112.6 (15)	F3_18—C2_18—F1_18	106.8 (4)
F3_6—C2_6—C1_6	110.6 (13)	F2_18—C2_18—C1_18	113.4 (4)
F4_6—C3_6—F5_6	105.4 (15)	F3_18—C2_18—C1_18	110.3 (4)
F4_6—C3_6—F6_6	107.9 (15)	F1_18—C2_18—C1_18	110.3 (4)
F5_6—C3_6—F6_6	111.1 (17)	F4_18—C3_18—F5_18	107.4 (4)
F4_6—C3_6—C1_6	111.4 (13)	F4_18—C3_18—F6_18	107.3 (4)
F5_6—C3_6—C1_6	109.9 (15)	F5_18—C3_18—F6_18	107.3 (5)
F6_6—C3_6—C1_6	110.9 (14)	F4_18—C3_18—C1_18	111.1 (4)
F8_6—C4_6—F7_6	109.1 (16)	F5_18—C3_18—C1_18	111.0 (4)
F8_6—C4_6—F9_6	104.8 (16)	F6_18—C3_18—C1_18	112.5 (4)
F7_6—C4_6—F9_6	110.8 (18)	F9_18—C4_18—F7_18	108.8 (5)
F8_6—C4_6—C1_6	111.6 (14)	F9_18—C4_18—F8_18	108.0 (4)
F7_6—C4_6—C1_6	110.5 (13)	F7_18—C4_18—F8_18	108.3 (4)
F9_6—C4_6—C1_6	109.9 (14)	F9_18—C4_18—C1_18	110.7 (4)
C1_7—O1_7—Al2	146.7 (3)	F7_18—C4_18—C1_18	109.0 (4)
O1_7—C1_7—C4_7	108.8 (4)	F8_18—C4_18—C1_18	112.0 (4)
O1_7—C1_7—C3_7	111.2 (4)	C1_19—O1_19—Ti2	172 (4)
C4_7—C1_7—C3_7	110.2 (4)	O1_19—C1_19—C2_19	109.4 (19)
O1_7—C1_7—C2_7	111.0 (4)	O1_19—C1_19—C3_19	109.1 (19)
C4_7—C1_7—C2_7	108.6 (5)	C2_19—C1_19—C3_19	110.5 (15)
C3_7—C1_7—C2_7	107.0 (4)	O1_19—C1_19—C4_19	107.5 (18)
F1_7—C2_7—F3_7	106.8 (6)	C2_19—C1_19—C4_19	110.5 (15)
F1_7—C2_7—F2_7	107.0 (5)	C3_19—C1_19—C4_19	109.8 (15)
F3_7—C2_7—F2_7	107.6 (5)	F3_19—C2_19—F2_19	109 (2)
F1_7—C2_7—C1_7	110.8 (5)	F3_19—C2_19—F1_19	108 (2)
F3_7—C2_7—C1_7	111.8 (5)	F2_19—C2_19—F1_19	107 (2)
F2_7—C2_7—C1_7	112.6 (5)	F3_19—C2_19—C1_19	111.5 (19)
F5_7—C3_7—F4_7	106.5 (5)	F2_19—C2_19—C1_19	111.5 (17)
F5_7—C3_7—F6_7	108.5 (4)	F1_19—C2_19—C1_19	110.1 (18)
F4_7—C3_7—F6_7	107.0 (5)	F5_19—C3_19—F6_19	107 (2)
F5_7—C3_7—C1_7	111.3 (4)	F5_19—C3_19—F4_19	110 (2)
F4_7—C3_7—C1_7	110.4 (5)	F6_19—C3_19—F4_19	108 (2)
F6_7—C3_7—C1_7	112.8 (4)	F5_19—C3_19—C1_19	111.2 (18)
F7_7—C4_7—F8_7	107.1 (5)	F6_19—C3_19—C1_19	111.7 (18)
F7_7—C4_7—F9_7	107.5 (4)	F4_19—C3_19—C1_19	108.6 (18)
F8_7—C4_7—F9_7	108.0 (5)	F8_19—C4_19—F7_19	108 (2)
F7_7—C4_7—C1_7	110.0 (4)	F8_19—C4_19—F9_19	109 (2)
F8_7—C4_7—C1_7	113.4 (4)	F7_19—C4_19—F9_19	109 (2)
F9_7—C4_7—C1_7	110.6 (5)	F8_19—C4_19—C1_19	111.7 (18)
C1_8—O1_8—Al2	151.2 (15)	F7_19—C4_19—C1_19	109.8 (17)
O1_8—C1_8—C3_8	110.7 (13)	F9_19—C4_19—C1_19	110.2 (19)
O1_8—C1_8—C2_8	109.4 (13)		
			
O1_1—C1_1—C2_1—F1_1	−69.9 (15)	O1_15—Al2—O1_11—C1_11	37.9 (8)
C4_1—C1_1—C2_1—F1_1	51.0 (12)	O1_5—Al2—O1_11—C1_11	159.6 (7)
C3_1—C1_1—C2_1—F1_1	169.1 (10)	Al2—O1_11—C1_11—C3_11	−91.1 (8)
O1_1—C1_1—C2_1—F3_1	50.2 (15)	Al2—O1_11—C1_11—C4_11	32.2 (9)
C4_1—C1_1—C2_1—F3_1	171.1 (9)	Al2—O1_11—C1_11—C2_11	150.4 (6)
C3_1—C1_1—C2_1—F3_1	−70.8 (11)	O1_11—C1_11—C2_11—F3_11	42.3 (5)
O1_1—C1_1—C2_1—F2_1	168.7 (14)	C3_11—C1_11—C2_11—F3_11	−76.5 (5)
C4_1—C1_1—C2_1—F2_1	−70.4 (12)	C4_11—C1_11—C2_11—F3_11	162.4 (4)
C3_1—C1_1—C2_1—F2_1	47.7 (12)	O1_11—C1_11—C2_11—F1_11	−80.0 (5)
O1_1—C1_1—C3_1—F5_1	−72.5 (14)	C3_11—C1_11—C2_11—F1_11	161.2 (4)
C4_1—C1_1—C3_1—F5_1	168.3 (9)	C4_11—C1_11—C2_11—F1_11	40.1 (5)
C2_1—C1_1—C3_1—F5_1	48.2 (11)	O1_11—C1_11—C2_11—F2_11	161.7 (4)
O1_1—C1_1—C3_1—F6_1	167.9 (13)	C3_11—C1_11—C2_11—F2_11	42.9 (5)
C4_1—C1_1—C3_1—F6_1	48.6 (10)	C4_11—C1_11—C2_11—F2_11	−78.1 (5)
C2_1—C1_1—C3_1—F6_1	−71.5 (10)	O1_11—C1_11—C3_11—F6_11	165.0 (4)
O1_1—C1_1—C3_1—F4_1	48.6 (15)	C4_11—C1_11—C3_11—F6_11	41.9 (5)
C4_1—C1_1—C3_1—F4_1	−70.7 (11)	C2_11—C1_11—C3_11—F6_11	−77.1 (5)
C2_1—C1_1—C3_1—F4_1	169.2 (9)	O1_11—C1_11—C3_11—F5_11	−72.0 (5)
O1_1—C1_1—C4_1—F9_1	45.4 (17)	C4_11—C1_11—C3_11—F5_11	164.9 (4)
C3_1—C1_1—C4_1—F9_1	165.6 (12)	C2_11—C1_11—C3_11—F5_11	45.9 (5)
C2_1—C1_1—C4_1—F9_1	−76.0 (14)	O1_11—C1_11—C3_11—F4_11	44.7 (5)
O1_1—C1_1—C4_1—F7_1	−79.7 (15)	C4_11—C1_11—C3_11—F4_11	−78.5 (5)
C3_1—C1_1—C4_1—F7_1	40.5 (12)	C2_11—C1_11—C3_11—F4_11	162.5 (4)
C2_1—C1_1—C4_1—F7_1	158.9 (10)	O1_11—C1_11—C4_11—F9_11	39.2 (6)
O1_1—C1_1—C4_1—F8_1	169.4 (14)	C3_11—C1_11—C4_11—F9_11	161.5 (4)
C3_1—C1_1—C4_1—F8_1	−70.4 (12)	C2_11—C1_11—C4_11—F9_11	−79.0 (5)
C2_1—C1_1—C4_1—F8_1	48.0 (13)	O1_11—C1_11—C4_11—F8_11	162.7 (4)
O1_2—C1_2—C2_2—F1_2	−64 (2)	C3_11—C1_11—C4_11—F8_11	−75.0 (5)
C4_2—C1_2—C2_2—F1_2	57.2 (16)	C2_11—C1_11—C4_11—F8_11	44.5 (5)
C3_2—C1_2—C2_2—F1_2	179.0 (13)	O1_11—C1_11—C4_11—F7_11	−79.6 (5)
O1_2—C1_2—C2_2—F2_2	167.6 (18)	C3_11—C1_11—C4_11—F7_11	42.7 (5)
C4_2—C1_2—C2_2—F2_2	−71.5 (14)	C2_11—C1_11—C4_11—F7_11	162.2 (4)
C3_2—C1_2—C2_2—F2_2	50.4 (14)	O1_6—Al2—O1_12—C1_12	−174 (5)
O1_2—C1_2—C2_2—F3_2	57.6 (19)	O1_16—Al2—O1_12—C1_12	53 (6)
C4_2—C1_2—C2_2—F3_2	178.5 (13)	O1_8—Al2—O1_12—C1_12	−61 (6)
C3_2—C1_2—C2_2—F3_2	−59.7 (14)	Al2—O1_12—C1_12—C4_12	−44 (6)
O1_2—C1_2—C3_2—F4_2	65.3 (19)	Al2—O1_12—C1_12—C2_12	81 (6)
C4_2—C1_2—C3_2—F4_2	−56.7 (15)	Al2—O1_12—C1_12—C3_12	−163 (5)
C2_2—C1_2—C3_2—F4_2	−177.8 (13)	O1_12—C1_12—C2_12—F1_12	−95 (3)
O1_2—C1_2—C3_2—F6_2	−171 (2)	C4_12—C1_12—C2_12—F1_12	31 (2)
C4_2—C1_2—C3_2—F6_2	66.8 (17)	C3_12—C1_12—C2_12—F1_12	144 (2)
C2_2—C1_2—C3_2—F6_2	−54.3 (17)	O1_12—C1_12—C2_12—F3_12	25 (3)
O1_2—C1_2—C3_2—F5_2	−55 (2)	C4_12—C1_12—C2_12—F3_12	151 (2)
C4_2—C1_2—C3_2—F5_2	−176.9 (12)	C3_12—C1_12—C2_12—F3_12	−95 (2)
C2_2—C1_2—C3_2—F5_2	62.0 (14)	O1_12—C1_12—C2_12—F2_12	142 (3)
O1_2—C1_2—C4_2—F7_2	−56 (2)	C4_12—C1_12—C2_12—F2_12	−92 (2)
C2_2—C1_2—C4_2—F7_2	−175.4 (13)	C3_12—C1_12—C2_12—F2_12	21 (2)
C3_2—C1_2—C4_2—F7_2	65.9 (15)	O1_12—C1_12—C3_12—F4_12	38 (3)
O1_2—C1_2—C4_2—F8_2	−174 (2)	C4_12—C1_12—C3_12—F4_12	−84 (3)
C2_2—C1_2—C4_2—F8_2	66.4 (17)	C2_12—C1_12—C3_12—F4_12	159 (3)
C3_2—C1_2—C4_2—F8_2	−52.3 (17)	O1_12—C1_12—C3_12—F5_12	−82 (3)
O1_2—C1_2—C4_2—F9_2	63 (2)	C4_12—C1_12—C3_12—F5_12	156 (3)
C2_2—C1_2—C4_2—F9_2	−57.0 (14)	C2_12—C1_12—C3_12—F5_12	39 (3)
C3_2—C1_2—C4_2—F9_2	−175.7 (12)	O1_12—C1_12—C3_12—F6_12	156 (3)
N3—Ti3—O1_3—C1_3	−144 (10)	C4_12—C1_12—C3_12—F6_12	34 (3)
N2—Ti3—O1_3—C1_3	124 (10)	C2_12—C1_12—C3_12—F6_12	−83 (2)
N4—Ti3—O1_3—C1_3	−56 (10)	O1_12—C1_12—C4_12—F9_12	44 (3)
N1—Ti3—O1_3—C1_3	38 (10)	C2_12—C1_12—C4_12—F9_12	−81 (3)
Ti3—O1_3—C1_3—C3_3	119 (10)	C3_12—C1_12—C4_12—F9_12	166 (3)
Ti3—O1_3—C1_3—C2_3	−2 (11)	O1_12—C1_12—C4_12—F8_12	161 (3)
Ti3—O1_3—C1_3—C4_3	−119 (10)	C2_12—C1_12—C4_12—F8_12	36 (2)
O1_3—C1_3—C2_3—F3_3	46.0 (17)	C3_12—C1_12—C4_12—F8_12	−77 (2)
C3_3—C1_3—C2_3—F3_3	−75.7 (14)	O1_12—C1_12—C4_12—F7_12	−84 (3)
C4_3—C1_3—C2_3—F3_3	163.5 (12)	C2_12—C1_12—C4_12—F7_12	151 (2)
O1_3—C1_3—C2_3—F1_3	−71.8 (16)	C3_12—C1_12—C4_12—F7_12	38 (2)
C3_3—C1_3—C2_3—F1_3	166.5 (11)	O1_9—Al1—O1_13—C1_13	−134.5 (7)
C4_3—C1_3—C2_3—F1_3	45.6 (13)	O1_17—Al1—O1_13—C1_13	−9.4 (8)
O1_3—C1_3—C2_3—F2_3	166.9 (18)	O1_14—Al1—O1_13—C1_13	108.3 (7)
C3_3—C1_3—C2_3—F2_3	45.2 (17)	O1_10—Al1—O1_13—C1_13	−125.7 (9)
C4_3—C1_3—C2_3—F2_3	−75.6 (17)	Al1—O1_13—C1_13—C3_13	−89.2 (8)
O1_3—C1_3—C3_3—F5_3	−76.1 (17)	Al1—O1_13—C1_13—C4_13	33.9 (9)
C2_3—C1_3—C3_3—F5_3	46.4 (14)	Al1—O1_13—C1_13—C2_13	152.8 (7)
C4_3—C1_3—C3_3—F5_3	164.8 (11)	O1_13—C1_13—C2_13—F1_13	−76.5 (5)
O1_3—C1_3—C3_3—F4_3	45.8 (19)	C3_13—C1_13—C2_13—F1_13	164.0 (5)
C2_3—C1_3—C3_3—F4_3	168.3 (14)	C4_13—C1_13—C2_13—F1_13	43.2 (6)
C4_3—C1_3—C3_3—F4_3	−73.3 (16)	O1_13—C1_13—C2_13—F3_13	44.9 (5)
O1_3—C1_3—C3_3—F6_3	166.0 (19)	C3_13—C1_13—C2_13—F3_13	−74.6 (5)
C2_3—C1_3—C3_3—F6_3	−71.5 (17)	C4_13—C1_13—C2_13—F3_13	164.6 (4)
C4_3—C1_3—C3_3—F6_3	47.0 (18)	O1_13—C1_13—C2_13—F2_13	163.8 (4)
O1_3—C1_3—C4_3—F7_3	−72.7 (17)	C3_13—C1_13—C2_13—F2_13	44.3 (5)
C3_3—C1_3—C4_3—F7_3	47.9 (15)	C4_13—C1_13—C2_13—F2_13	−76.5 (5)
C2_3—C1_3—C4_3—F7_3	167.2 (12)	O1_13—C1_13—C3_13—F5_13	−75.6 (5)
O1_3—C1_3—C4_3—F8_3	163.9 (18)	C4_13—C1_13—C3_13—F5_13	161.6 (4)
C3_3—C1_3—C4_3—F8_3	−75.5 (17)	C2_13—C1_13—C3_13—F5_13	41.8 (6)
C2_3—C1_3—C4_3—F8_3	43.9 (17)	O1_13—C1_13—C3_13—F6_13	163.8 (4)
O1_3—C1_3—C4_3—F9_3	43.3 (17)	C4_13—C1_13—C3_13—F6_13	41.0 (6)
C3_3—C1_3—C4_3—F9_3	163.9 (12)	C2_13—C1_13—C3_13—F6_13	−78.9 (5)
C2_3—C1_3—C4_3—F9_3	−76.8 (13)	O1_13—C1_13—C3_13—F4_13	42.5 (7)
O1_4—C1_4—C2_4—F3_4	70.3 (12)	C4_13—C1_13—C3_13—F4_13	−80.3 (6)
C4_4—C1_4—C2_4—F3_4	−170.4 (9)	C2_13—C1_13—C3_13—F4_13	159.8 (5)
C3_4—C1_4—C2_4—F3_4	−49.2 (10)	O1_13—C1_13—C4_13—F9_13	38.0 (6)
O1_4—C1_4—C2_4—F1_4	−49.5 (12)	C3_13—C1_13—C4_13—F9_13	160.9 (4)
C4_4—C1_4—C2_4—F1_4	69.8 (10)	C2_13—C1_13—C4_13—F9_13	−79.7 (5)
C3_4—C1_4—C2_4—F1_4	−169.0 (9)	O1_13—C1_13—C4_13—F7_13	−79.8 (5)
O1_4—C1_4—C2_4—F2_4	−166.0 (12)	C3_13—C1_13—C4_13—F7_13	43.2 (5)
C4_4—C1_4—C2_4—F2_4	−46.7 (13)	C2_13—C1_13—C4_13—F7_13	162.5 (4)
C3_4—C1_4—C2_4—F2_4	74.5 (12)	O1_13—C1_13—C4_13—F8_13	157.8 (5)
O1_4—C1_4—C3_4—F5_4	−44.1 (13)	C3_13—C1_13—C4_13—F8_13	−79.3 (6)
C4_4—C1_4—C3_4—F5_4	−163.8 (9)	C2_13—C1_13—C4_13—F8_13	40.0 (7)
C2_4—C1_4—C3_4—F5_4	75.5 (10)	O1_20—C1_20—C2_20—F3_20	45.2 (4)
O1_4—C1_4—C3_4—F6_4	−164.8 (12)	C4_20—C1_20—C2_20—F3_20	165.1 (3)
C4_4—C1_4—C3_4—F6_4	75.5 (12)	C3_20—C1_20—C2_20—F3_20	−74.6 (4)
C2_4—C1_4—C3_4—F6_4	−45.2 (12)	O1_20—C1_20—C2_20—F1_20	−75.1 (4)
O1_4—C1_4—C3_4—F4_4	74.1 (12)	C4_20—C1_20—C2_20—F1_20	44.8 (5)
C4_4—C1_4—C3_4—F4_4	−45.6 (10)	C3_20—C1_20—C2_20—F1_20	165.1 (4)
C2_4—C1_4—C3_4—F4_4	−166.3 (8)	O1_20—C1_20—C2_20—F2_20	165.1 (3)
O1_4—C1_4—C4_4—F9_4	77.9 (12)	C4_20—C1_20—C2_20—F2_20	−74.9 (4)
C3_4—C1_4—C4_4—F9_4	−161.8 (9)	C3_20—C1_20—C2_20—F2_20	45.4 (5)
C2_4—C1_4—C4_4—F9_4	−42.1 (11)	O1_20—C1_20—C3_20—F5_20	−70.6 (4)
O1_4—C1_4—C4_4—F7_4	−44.0 (13)	C4_20—C1_20—C3_20—F5_20	169.7 (4)
C3_4—C1_4—C4_4—F7_4	76.3 (11)	C2_20—C1_20—C3_20—F5_20	48.9 (4)
C2_4—C1_4—C4_4—F7_4	−164.1 (9)	O1_20—C1_20—C3_20—F4_20	48.6 (5)
O1_4—C1_4—C4_4—F8_4	−163.4 (14)	C4_20—C1_20—C3_20—F4_20	−71.2 (4)
C3_4—C1_4—C4_4—F8_4	−43.1 (14)	C2_20—C1_20—C3_20—F4_20	168.1 (4)
C2_4—C1_4—C4_4—F8_4	76.5 (13)	O1_20—C1_20—C3_20—F6_20	167.9 (4)
O1_11—Al2—O1_5—C1_5	−40.6 (12)	C4_20—C1_20—C3_20—F6_20	48.1 (5)
O1_7—Al2—O1_5—C1_5	−165.3 (10)	C2_20—C1_20—C3_20—F6_20	−72.6 (5)
O1_15—Al2—O1_5—C1_5	77.8 (11)	O1_20—C1_20—C4_20—F7_20	−71.2 (4)
Al2—O1_5—C1_5—C3_5	75.0 (12)	C2_20—C1_20—C4_20—F7_20	169.0 (3)
Al2—O1_5—C1_5—C4_5	−164.7 (10)	C3_20—C1_20—C4_20—F7_20	48.6 (5)
Al2—O1_5—C1_5—C2_5	−45.6 (12)	O1_20—C1_20—C4_20—F8_20	167.4 (4)
O1_5—C1_5—C2_5—F3_5	43.2 (7)	C2_20—C1_20—C4_20—F8_20	47.5 (5)
C3_5—C1_5—C2_5—F3_5	−79.0 (6)	C3_20—C1_20—C4_20—F8_20	−72.8 (5)
C4_5—C1_5—C2_5—F3_5	161.7 (5)	O1_20—C1_20—C4_20—F9_20	47.6 (5)
O1_5—C1_5—C2_5—F1_5	−75.3 (6)	C2_20—C1_20—C4_20—F9_20	−72.2 (5)
C3_5—C1_5—C2_5—F1_5	162.5 (4)	C3_20—C1_20—C4_20—F9_20	167.5 (4)
C4_5—C1_5—C2_5—F1_5	43.2 (6)	O1_13—Al1—O1_14—C1_14	61.6 (6)
O1_5—C1_5—C2_5—F2_5	163.7 (6)	O1_9—Al1—O1_14—C1_14	−61.6 (6)
C3_5—C1_5—C2_5—F2_5	41.5 (6)	O1_17—Al1—O1_14—C1_14	−178.8 (6)
C4_5—C1_5—C2_5—F2_5	−77.8 (6)	O1_10—Al1—O1_14—C1_14	−40.4 (8)
O1_5—C1_5—C3_5—F4_5	41.1 (6)	Al1—O1_14—C1_14—C3_14	−150.1 (5)
C4_5—C1_5—C3_5—F4_5	−78.0 (6)	Al1—O1_14—C1_14—C2_14	92.2 (6)
C2_5—C1_5—C3_5—F4_5	163.4 (5)	Al1—O1_14—C1_14—C4_14	−29.7 (7)
O1_5—C1_5—C3_5—F5_5	−79.0 (6)	O1_14—C1_14—C2_14—F2_14	159.8 (4)
C4_5—C1_5—C3_5—F5_5	161.9 (5)	C3_14—C1_14—C2_14—F2_14	42.9 (5)
C2_5—C1_5—C3_5—F5_5	43.3 (6)	C4_14—C1_14—C2_14—F2_14	−76.9 (5)
O1_5—C1_5—C3_5—F6_5	160.8 (5)	O1_14—C1_14—C2_14—F3_14	37.7 (5)
C4_5—C1_5—C3_5—F6_5	41.7 (6)	C3_14—C1_14—C2_14—F3_14	−79.2 (4)
C2_5—C1_5—C3_5—F6_5	−76.9 (6)	C4_14—C1_14—C2_14—F3_14	161.1 (4)
O1_5—C1_5—C4_5—F9_5	42.6 (7)	O1_14—C1_14—C2_14—F1_14	−80.2 (5)
C3_5—C1_5—C4_5—F9_5	163.7 (5)	C3_14—C1_14—C2_14—F1_14	162.9 (4)
C2_5—C1_5—C4_5—F9_5	−78.0 (6)	C4_14—C1_14—C2_14—F1_14	43.1 (5)
O1_5—C1_5—C4_5—F8_5	164.2 (5)	O1_14—C1_14—C3_14—F4_14	45.2 (5)
C3_5—C1_5—C4_5—F8_5	−74.7 (6)	C2_14—C1_14—C3_14—F4_14	164.4 (4)
C2_5—C1_5—C4_5—F8_5	43.6 (6)	C4_14—C1_14—C3_14—F4_14	−76.3 (5)
O1_5—C1_5—C4_5—F7_5	−75.9 (6)	O1_14—C1_14—C3_14—F5_14	−74.9 (5)
C3_5—C1_5—C4_5—F7_5	45.2 (6)	C2_14—C1_14—C3_14—F5_14	44.3 (5)
C2_5—C1_5—C4_5—F7_5	163.5 (5)	C4_14—C1_14—C3_14—F5_14	163.7 (4)
O1_16—Al2—O1_6—C1_6	134 (7)	O1_14—C1_14—C3_14—F6_14	165.7 (4)
O1_8—Al2—O1_6—C1_6	−108 (7)	C2_14—C1_14—C3_14—F6_14	−75.1 (5)
O1_12—Al2—O1_6—C1_6	−1 (8)	C4_14—C1_14—C3_14—F6_14	44.3 (5)
Al2—O1_6—C1_6—C3_6	−52 (8)	O1_14—C1_14—C4_14—F9_14	44.9 (5)
Al2—O1_6—C1_6—C4_6	68 (7)	C3_14—C1_14—C4_14—F9_14	163.5 (4)
Al2—O1_6—C1_6—C2_6	−171 (7)	C2_14—C1_14—C4_14—F9_14	−77.8 (4)
O1_6—C1_6—C2_6—F1_6	−45 (2)	O1_14—C1_14—C4_14—F7_14	−73.9 (5)
C3_6—C1_6—C2_6—F1_6	−165.7 (15)	C3_14—C1_14—C4_14—F7_14	44.7 (5)
C4_6—C1_6—C2_6—F1_6	75.9 (17)	C2_14—C1_14—C4_14—F7_14	163.5 (4)
O1_6—C1_6—C2_6—F2_6	−159 (2)	O1_14—C1_14—C4_14—F8_14	165.3 (4)
C3_6—C1_6—C2_6—F2_6	79.8 (18)	C3_14—C1_14—C4_14—F8_14	−76.1 (5)
C4_6—C1_6—C2_6—F2_6	−38.6 (19)	C2_14—C1_14—C4_14—F8_14	42.6 (5)
O1_6—C1_6—C2_6—F3_6	75 (2)	O1_11—Al2—O1_15—C1_15	−158.2 (6)
C3_6—C1_6—C2_6—F3_6	−45.9 (17)	O1_7—Al2—O1_15—C1_15	−34.2 (6)
C4_6—C1_6—C2_6—F3_6	−164.3 (15)	O1_5—Al2—O1_15—C1_15	83.2 (7)
O1_6—C1_6—C3_6—F4_6	76 (2)	Al2—O1_15—C1_15—C4_15	−64.5 (7)
C4_6—C1_6—C3_6—F4_6	−45.2 (18)	Al2—O1_15—C1_15—C3_15	175.5 (5)
C2_6—C1_6—C3_6—F4_6	−164.4 (15)	Al2—O1_15—C1_15—C2_15	57.6 (7)
O1_6—C1_6—C3_6—F5_6	−40 (2)	O1_15—C1_15—C2_15—F1_15	−81.3 (5)
C4_6—C1_6—C3_6—F5_6	−161.7 (16)	C4_15—C1_15—C2_15—F1_15	41.3 (5)
C2_6—C1_6—C3_6—F5_6	79.1 (17)	C3_15—C1_15—C2_15—F1_15	162.0 (4)
O1_6—C1_6—C3_6—F6_6	−164 (2)	O1_15—C1_15—C2_15—F3_15	38.0 (5)
C4_6—C1_6—C3_6—F6_6	75.1 (18)	C4_15—C1_15—C2_15—F3_15	160.6 (4)
C2_6—C1_6—C3_6—F6_6	−44.1 (18)	C3_15—C1_15—C2_15—F3_15	−78.8 (5)
O1_6—C1_6—C4_6—F8_6	−166 (2)	O1_15—C1_15—C2_15—F2_15	158.3 (4)
C3_6—C1_6—C4_6—F8_6	−43.9 (19)	C4_15—C1_15—C2_15—F2_15	−79.1 (5)
C2_6—C1_6—C4_6—F8_6	73.9 (18)	C3_15—C1_15—C2_15—F2_15	41.6 (6)
O1_6—C1_6—C4_6—F7_6	−44 (3)	O1_15—C1_15—C3_15—F4_15	44.3 (5)
C3_6—C1_6—C4_6—F7_6	77.7 (19)	C4_15—C1_15—C3_15—F4_15	−76.0 (5)
C2_6—C1_6—C4_6—F7_6	−164.5 (18)	C2_15—C1_15—C3_15—F4_15	163.4 (4)
O1_6—C1_6—C4_6—F9_6	78 (2)	O1_15—C1_15—C3_15—F6_15	165.6 (4)
C3_6—C1_6—C4_6—F9_6	−159.7 (17)	C4_15—C1_15—C3_15—F6_15	45.3 (5)
C2_6—C1_6—C4_6—F9_6	−41.9 (19)	C2_15—C1_15—C3_15—F6_15	−75.3 (5)
O1_11—Al2—O1_7—C1_7	−79.2 (6)	O1_15—C1_15—C3_15—F5_15	−74.8 (5)
O1_15—Al2—O1_7—C1_7	161.8 (6)	C4_15—C1_15—C3_15—F5_15	164.9 (4)
O1_5—Al2—O1_7—C1_7	40.9 (7)	C2_15—C1_15—C3_15—F5_15	44.3 (5)
Al2—O1_7—C1_7—C4_7	−169.0 (5)	O1_15—C1_15—C4_15—F7_15	−73.1 (5)
Al2—O1_7—C1_7—C3_7	69.5 (7)	C3_15—C1_15—C4_15—F7_15	44.7 (5)
Al2—O1_7—C1_7—C2_7	−49.5 (8)	C2_15—C1_15—C4_15—F7_15	164.5 (4)
O1_7—C1_7—C2_7—F1_7	−41.1 (6)	O1_15—C1_15—C4_15—F9_15	46.0 (6)
C4_7—C1_7—C2_7—F1_7	78.5 (6)	C3_15—C1_15—C4_15—F9_15	163.9 (5)
C3_7—C1_7—C2_7—F1_7	−162.6 (5)	C2_15—C1_15—C4_15—F9_15	−76.4 (5)
O1_7—C1_7—C2_7—F3_7	77.9 (6)	O1_15—C1_15—C4_15—F8_15	165.7 (4)
C4_7—C1_7—C2_7—F3_7	−162.6 (5)	C3_15—C1_15—C4_15—F8_15	−76.4 (5)
C3_7—C1_7—C2_7—F3_7	−43.6 (6)	C2_15—C1_15—C4_15—F8_15	43.3 (5)
O1_7—C1_7—C2_7—F2_7	−160.9 (5)	O1_6—Al2—O1_16—C1_16	−41 (4)
C4_7—C1_7—C2_7—F2_7	−41.4 (6)	O1_8—Al2—O1_16—C1_16	−163 (3)
C3_7—C1_7—C2_7—F2_7	77.6 (6)	O1_12—Al2—O1_16—C1_16	84 (4)
O1_7—C1_7—C3_7—F5_7	−43.8 (6)	Al2—O1_16—C1_16—C2_16	57 (4)
C4_7—C1_7—C3_7—F5_7	−164.4 (5)	Al2—O1_16—C1_16—C4_16	−63 (4)
C2_7—C1_7—C3_7—F5_7	77.6 (6)	Al2—O1_16—C1_16—C3_16	177 (3)
O1_7—C1_7—C3_7—F4_7	74.3 (6)	O1_16—C1_16—C2_16—F1_16	−79 (2)
C4_7—C1_7—C3_7—F4_7	−46.4 (6)	C4_16—C1_16—C2_16—F1_16	42 (2)
C2_7—C1_7—C3_7—F4_7	−164.3 (5)	C3_16—C1_16—C2_16—F1_16	161.7 (18)
O1_7—C1_7—C3_7—F6_7	−166.0 (5)	O1_16—C1_16—C2_16—F2_16	161 (2)
C4_7—C1_7—C3_7—F6_7	73.3 (6)	C4_16—C1_16—C2_16—F2_16	−78 (2)
C2_7—C1_7—C3_7—F6_7	−44.6 (6)	C3_16—C1_16—C2_16—F2_16	42 (2)
O1_7—C1_7—C4_7—F7_7	−44.7 (6)	O1_16—C1_16—C2_16—F3_16	43 (3)
C3_7—C1_7—C4_7—F7_7	77.4 (5)	C4_16—C1_16—C2_16—F3_16	163 (2)
C2_7—C1_7—C4_7—F7_7	−165.6 (5)	C3_16—C1_16—C2_16—F3_16	−77 (2)
O1_7—C1_7—C4_7—F8_7	−164.6 (5)	O1_16—C1_16—C3_16—F6_16	168 (2)
C3_7—C1_7—C4_7—F8_7	−42.5 (7)	C2_16—C1_16—C3_16—F6_16	−72 (2)
C2_7—C1_7—C4_7—F8_7	74.4 (6)	C4_16—C1_16—C3_16—F6_16	48 (2)
O1_7—C1_7—C4_7—F9_7	73.9 (5)	O1_16—C1_16—C3_16—F5_16	−72 (2)
C3_7—C1_7—C4_7—F9_7	−164.1 (4)	C2_16—C1_16—C3_16—F5_16	48.0 (19)
C2_7—C1_7—C4_7—F9_7	−47.1 (5)	C4_16—C1_16—C3_16—F5_16	168.3 (17)
O1_6—Al2—O1_8—C1_8	−71 (3)	O1_16—C1_16—C3_16—F4_16	48 (2)
O1_16—Al2—O1_8—C1_8	55 (3)	C2_16—C1_16—C3_16—F4_16	167.7 (16)
O1_12—Al2—O1_8—C1_8	−179 (3)	C4_16—C1_16—C3_16—F4_16	−72.0 (18)
Al2—O1_8—C1_8—C3_8	36 (3)	O1_16—C1_16—C4_16—F9_16	47 (2)
Al2—O1_8—C1_8—C2_8	−84 (3)	C2_16—C1_16—C4_16—F9_16	−74.0 (19)
Al2—O1_8—C1_8—C4_8	158 (3)	C3_16—C1_16—C4_16—F9_16	166.0 (17)
O1_8—C1_8—C2_8—F1_8	−162.4 (17)	O1_16—C1_16—C4_16—F7_16	−72 (2)
C3_8—C1_8—C2_8—F1_8	76.5 (17)	C2_16—C1_16—C4_16—F7_16	167.7 (18)
C4_8—C1_8—C2_8—F1_8	−42.7 (18)	C3_16—C1_16—C4_16—F7_16	48 (2)
O1_8—C1_8—C2_8—F3_8	−43.6 (19)	O1_16—C1_16—C4_16—F8_16	166.0 (19)
C3_8—C1_8—C2_8—F3_8	−164.7 (15)	C2_16—C1_16—C4_16—F8_16	45 (2)
C4_8—C1_8—C2_8—F3_8	76.1 (17)	C3_16—C1_16—C4_16—F8_16	−74 (2)
O1_8—C1_8—C2_8—F2_8	75.6 (19)	O1_13—Al1—O1_17—C1_17	150.0 (6)
C3_8—C1_8—C2_8—F2_8	−45.5 (19)	O1_9—Al1—O1_17—C1_17	−83.0 (6)
C4_8—C1_8—C2_8—F2_8	−164.8 (17)	O1_14—Al1—O1_17—C1_17	29.6 (7)
O1_8—C1_8—C3_8—F6_8	−165.2 (18)	O1_10—Al1—O1_17—C1_17	−112.9 (8)
C2_8—C1_8—C3_8—F6_8	−45 (2)	Al1—O1_17—C1_17—C2_17	51.2 (8)
C4_8—C1_8—C3_8—F6_8	73.5 (19)	Al1—O1_17—C1_17—C4_17	−69.5 (7)
O1_8—C1_8—C3_8—F4_8	71 (2)	Al1—O1_17—C1_17—C3_17	171.5 (5)
C2_8—C1_8—C3_8—F4_8	−168.4 (18)	O1_17—C1_17—C2_17—F1_17	−43.1 (5)
C4_8—C1_8—C3_8—F4_8	−50.0 (19)	C4_17—C1_17—C2_17—F1_17	78.1 (4)
O1_8—C1_8—C3_8—F5_8	−44 (2)	C3_17—C1_17—C2_17—F1_17	−162.5 (4)
C2_8—C1_8—C3_8—F5_8	76.1 (19)	O1_17—C1_17—C2_17—F2_17	−164.1 (3)
C4_8—C1_8—C3_8—F5_8	−165.4 (17)	C4_17—C1_17—C2_17—F2_17	−42.9 (4)
O1_8—C1_8—C4_8—F7_8	−46.2 (19)	C3_17—C1_17—C2_17—F2_17	76.5 (5)
C3_8—C1_8—C4_8—F7_8	75.7 (18)	O1_17—C1_17—C2_17—F3_17	75.3 (4)
C2_8—C1_8—C4_8—F7_8	−165.6 (16)	C4_17—C1_17—C2_17—F3_17	−163.5 (3)
O1_8—C1_8—C4_8—F9_8	76.1 (19)	C3_17—C1_17—C2_17—F3_17	−44.1 (5)
C3_8—C1_8—C4_8—F9_8	−162.1 (17)	O1_17—C1_17—C3_17—F5_17	−43.8 (5)
C2_8—C1_8—C4_8—F9_8	−43.4 (19)	C2_17—C1_17—C3_17—F5_17	78.2 (5)
O1_8—C1_8—C4_8—F8_8	−165.5 (17)	C4_17—C1_17—C3_17—F5_17	−162.8 (4)
C3_8—C1_8—C4_8—F8_8	−43.6 (18)	O1_17—C1_17—C3_17—F4_17	75.5 (5)
C2_8—C1_8—C4_8—F8_8	75.0 (18)	C2_17—C1_17—C3_17—F4_17	−162.5 (4)
O1_13—Al1—O1_9—C1_9	37.9 (9)	C4_17—C1_17—C3_17—F4_17	−43.5 (5)
O1_17—Al1—O1_9—C1_9	−86.6 (8)	O1_17—C1_17—C3_17—F6_17	−165.0 (4)
O1_14—Al1—O1_9—C1_9	159.1 (8)	C2_17—C1_17—C3_17—F6_17	−43.0 (6)
Al1—O1_9—C1_9—C4_9	24.1 (11)	C4_17—C1_17—C3_17—F6_17	76.0 (5)
Al1—O1_9—C1_9—C2_9	−97.3 (9)	O1_17—C1_17—C4_17—F7_17	−39.8 (5)
Al1—O1_9—C1_9—C3_9	145.1 (7)	C2_17—C1_17—C4_17—F7_17	−162.5 (4)
O1_9—C1_9—C2_9—F1_9	46.4 (7)	C3_17—C1_17—C4_17—F7_17	78.2 (4)
C4_9—C1_9—C2_9—F1_9	−76.9 (7)	O1_17—C1_17—C4_17—F9_17	79.9 (4)
C3_9—C1_9—C2_9—F1_9	162.6 (6)	C2_17—C1_17—C4_17—F9_17	−42.8 (4)
O1_9—C1_9—C2_9—F2_9	166.1 (6)	C3_17—C1_17—C4_17—F9_17	−162.1 (3)
C4_9—C1_9—C2_9—F2_9	42.8 (8)	O1_17—C1_17—C4_17—F8_17	−160.5 (3)
C3_9—C1_9—C2_9—F2_9	−77.7 (7)	C2_17—C1_17—C4_17—F8_17	76.8 (4)
O1_9—C1_9—C2_9—F3_9	−72.6 (8)	C3_17—C1_17—C4_17—F8_17	−42.5 (5)
C4_9—C1_9—C2_9—F3_9	164.2 (8)	O1_18—C1_18—C2_18—F2_18	−166.0 (4)
C3_9—C1_9—C2_9—F3_9	43.6 (9)	C3_18—C1_18—C2_18—F2_18	74.0 (5)
O1_9—C1_9—C3_9—F5_9	41.1 (7)	C4_18—C1_18—C2_18—F2_18	−46.7 (5)
C4_9—C1_9—C3_9—F5_9	163.7 (6)	O1_18—C1_18—C2_18—F3_18	72.4 (5)
C2_9—C1_9—C3_9—F5_9	−76.2 (7)	C3_18—C1_18—C2_18—F3_18	−47.6 (5)
O1_9—C1_9—C3_9—F4_9	−77.0 (7)	C4_18—C1_18—C2_18—F3_18	−168.3 (4)
C4_9—C1_9—C3_9—F4_9	45.6 (8)	O1_18—C1_18—C2_18—F1_18	−45.4 (5)
C2_9—C1_9—C3_9—F4_9	165.7 (6)	C3_18—C1_18—C2_18—F1_18	−165.3 (4)
O1_9—C1_9—C3_9—F6_9	162.0 (6)	C4_18—C1_18—C2_18—F1_18	74.0 (5)
C4_9—C1_9—C3_9—F6_9	−75.4 (7)	O1_18—C1_18—C3_18—F4_18	74.7 (5)
C2_9—C1_9—C3_9—F6_9	44.8 (7)	C4_18—C1_18—C3_18—F4_18	−46.0 (5)
O1_9—C1_9—C4_9—F7_9	39.6 (7)	C2_18—C1_18—C3_18—F4_18	−165.8 (4)
C2_9—C1_9—C4_9—F7_9	160.5 (6)	O1_18—C1_18—C3_18—F5_18	−44.8 (5)
C3_9—C1_9—C4_9—F7_9	−79.6 (7)	C4_18—C1_18—C3_18—F5_18	−165.4 (4)
O1_9—C1_9—C4_9—F8_9	159.9 (6)	C2_18—C1_18—C3_18—F5_18	74.8 (5)
C2_9—C1_9—C4_9—F8_9	−79.2 (7)	O1_18—C1_18—C3_18—F6_18	−165.0 (4)
C3_9—C1_9—C4_9—F8_9	40.7 (8)	C4_18—C1_18—C3_18—F6_18	74.4 (5)
O1_9—C1_9—C4_9—F9_9	−78.2 (7)	C2_18—C1_18—C3_18—F6_18	−45.4 (6)
C2_9—C1_9—C4_9—F9_9	42.7 (7)	O1_18—C1_18—C4_18—F9_18	73.2 (5)
C3_9—C1_9—C4_9—F9_9	162.6 (6)	C3_18—C1_18—C4_18—F9_18	−165.9 (4)
O1_13—Al1—O1_10—C1_10	176 (2)	C2_18—C1_18—C4_18—F9_18	−46.1 (5)
O1_17—Al1—O1_10—C1_10	65 (3)	O1_18—C1_18—C4_18—F7_18	−46.4 (5)
O1_14—Al1—O1_10—C1_10	−70 (3)	C3_18—C1_18—C4_18—F7_18	74.5 (5)
Al1—O1_10—C1_10—C4_10	121 (2)	C2_18—C1_18—C4_18—F7_18	−165.7 (4)
Al1—O1_10—C1_10—C3_10	2 (3)	O1_18—C1_18—C4_18—F8_18	−166.1 (4)
Al1—O1_10—C1_10—C2_10	−118 (2)	C3_18—C1_18—C4_18—F8_18	−45.2 (5)
O1_10—C1_10—C2_10—F2_10	167.3 (15)	C2_18—C1_18—C4_18—F8_18	74.6 (5)
C4_10—C1_10—C2_10—F2_10	−72.9 (17)	O1_19—C1_19—C2_19—F3_19	57 (3)
C3_10—C1_10—C2_10—F2_10	45.1 (17)	C3_19—C1_19—C2_19—F3_19	−63 (3)
O1_10—C1_10—C2_10—F3_10	47.5 (18)	C4_19—C1_19—C2_19—F3_19	175 (3)
C4_10—C1_10—C2_10—F3_10	167.3 (15)	O1_19—C1_19—C2_19—F2_19	179 (3)
C3_10—C1_10—C2_10—F3_10	−74.7 (17)	C3_19—C1_19—C2_19—F2_19	59 (3)
O1_10—C1_10—C2_10—F1_10	−72.9 (15)	C4_19—C1_19—C2_19—F2_19	−63 (3)
C4_10—C1_10—C2_10—F1_10	46.9 (16)	O1_19—C1_19—C2_19—F1_19	−63 (3)
C3_10—C1_10—C2_10—F1_10	164.9 (13)	C3_19—C1_19—C2_19—F1_19	177 (3)
O1_10—C1_10—C3_10—F4_10	42.1 (17)	C4_19—C1_19—C2_19—F1_19	55 (3)
C4_10—C1_10—C3_10—F4_10	−77.1 (16)	O1_19—C1_19—C3_19—F5_19	−69 (3)
C2_10—C1_10—C3_10—F4_10	163.7 (14)	C2_19—C1_19—C3_19—F5_19	52 (3)
O1_10—C1_10—C3_10—F5_10	−77.0 (17)	C4_19—C1_19—C3_19—F5_19	174 (3)
C4_10—C1_10—C3_10—F5_10	163.8 (16)	O1_19—C1_19—C3_19—F6_19	172 (3)
C2_10—C1_10—C3_10—F5_10	44.5 (17)	C2_19—C1_19—C3_19—F6_19	−68 (3)
O1_10—C1_10—C3_10—F6_10	161.8 (14)	C4_19—C1_19—C3_19—F6_19	54 (3)
C4_10—C1_10—C3_10—F6_10	42.5 (16)	O1_19—C1_19—C3_19—F4_19	53 (3)
C2_10—C1_10—C3_10—F6_10	−76.7 (15)	C2_19—C1_19—C3_19—F4_19	173 (3)
O1_10—C1_10—C4_10—F8_10	160.1 (15)	C4_19—C1_19—C3_19—F4_19	−64 (3)
C3_10—C1_10—C4_10—F8_10	−78.6 (17)	O1_19—C1_19—C4_19—F8_19	−179 (3)
C2_10—C1_10—C4_10—F8_10	39.0 (18)	C2_19—C1_19—C4_19—F8_19	61 (3)
O1_10—C1_10—C4_10—F9_10	38.6 (18)	C3_19—C1_19—C4_19—F8_19	−61 (3)
C3_10—C1_10—C4_10—F9_10	159.8 (15)	O1_19—C1_19—C4_19—F7_19	−60 (3)
C2_10—C1_10—C4_10—F9_10	−82.6 (17)	C2_19—C1_19—C4_19—F7_19	−179 (3)
O1_10—C1_10—C4_10—F7_10	−79 (2)	C3_19—C1_19—C4_19—F7_19	58 (3)
C3_10—C1_10—C4_10—F7_10	42 (2)	O1_19—C1_19—C4_19—F9_19	59 (3)
C2_10—C1_10—C4_10—F7_10	160 (2)	C2_19—C1_19—C4_19—F9_19	−60 (3)
O1_7—Al2—O1_11—C1_11	−80.7 (8)	C3_19—C1_19—C4_19—F9_19	178 (3)

Symmetry codes: (i) −*x*, −*y*, −*z*; (ii) −*x*, −*y*+1, −*z*.

### ?

**Table:**
