# Supplementary material for: Crystal structure and computational analysis of tetra­kis­(aceto­nitrile)­bis­(nona­fluoro-tert-butanolato)titanium(III) complex as a salt of the weakly coordinating [Al{OC(CF3)3}4]− anion
Source: Acta Crystallogr E Crystallogr Commun. 2026 Jan 1;82(Pt 1):86–90. doi: 10.1107/S2056989025011314 (PMC12810303; doi:10.1107/S2056989025011314)
Supplement: Supplementary file 4 [file e-82-00086-sup4.docx]

Supplementary Information

**Details of DFT computations.**

Calculations were performed using Orca 6 package (Neese, 2025), ωB97X-V10 (Mardirossian & Head-Gordon, 2014) functional with def2-TZVP basis set (Weigend & Alrichs, 2005), def2/j auxiliary basis set (Weigend, 2006) and D4 dispersion correction (Caldeweyher *et al.*, 2020). No imaginary modes are present in calculated IR spectra meaning that the structures are in energy minimum.

**Energies and coordinates**

|  | | |
| --- | --- | --- |
|  | | |
| **OC(CF_3_)_3­_^−^** | | **OC(CH_3_)_3­_^−^** |
| E_scf_ | -1126.9328799 | -233.25792093 |
| H | -1126.86663392 | -233.1295651 |
| G | -1126.91768475 | -233.16565360 |
| F -5.18273039350649 3.07226600946650 13.78812605863099  F -6.08490122732957 4.19553343706024 12.17114823848830  C -5.07613022795370 3.33315026611421 12.46732609764439  F -5.35969769580510 2.19038592515355 11.82859486748468  F -2.85200588426258 4.43417155512463 14.31847003569602  F -4.25161394928976 5.83646178309543 13.45897909760782  C -3.24745486510258 4.97085812998200 13.15660672694651  C -3.61537538044283 3.83978327620434 12.10395035949834  F -2.22080648639233 5.72878509493537 12.71429430289895  F -4.33301361204956 5.76011904815048 10.68083616625683  C -3.71559455027949 4.54852451682518 10.68613607343454  F -4.39417676308881 3.77945392167493 9.80739051503661  F -2.49905664412425 4.74465664852808 10.16122868451948  O -2.75286232037293 2.87557038768504 12.10525277585647 | | H -4.45825677982305 3.18363417008594 14.07142590395381  H -5.26852013379547 2.42551394223144 12.69177475599338  H -5.67618744254072 4.10574102616041 13.14437582249434  C -4.86024296020063 3.36962896492387 13.06921877481673  H -2.72955104532030 4.99366663020914 13.68591850940976  C -3.68717556619644 3.80771630059045 12.13760921969716  O -2.72657895914318 2.87974808793432 12.07688427331890  C -3.14981111107288 5.16543202602137 12.68873657058202  H -3.91123963171918 5.95853457986981 12.75512463281559  H -4.71324864723967 3.16330549549058 10.33352763010500  C -4.30426424346039 4.09776860259698 10.73363021632800  H -5.09881919408527 4.86053530836543 10.73904075948293  H -2.33777316538638 5.50788167306951 12.03763945657215  H -3.50229112001641 4.42646319245073 10.06332347443016 |
|  | | |
|  | | |
| **[Ti{OC(CF)_3_)_3_}(ACN)_4_]^+^** | | **[Ti{OC(CH)_3_)_3_}(ACN)_4_]^+^** |
| E_scf_ | -3634.68327531 | -1847.45965366 |
| H | -3634.33149075 | -1846.98230434 |
| G | -3634.45803946 | -1847.08061702 |
| O -0.05860048421868 0.41631484321197 11.78533130469638  C -4.41987441624983 -2.06356463637027 12.25425426760826  H -5.36752844536040 -1.84702390391618 11.75935575062539  H -4.60331050284321 -2.30361504976701 13.30231921012850  H -3.93463720795681 -2.91024128135266 11.76637422653378  F -0.37878688284809 -1.45287223343059 13.64913449058791  F 1.46416179459481 -0.43410960166411 14.10288594523692  F 0.09155229131389 -1.03887698701613 9.58403593396513  F 3.24894801055660 -0.83093806214107 11.75432264919834  F 1.51174373260651 -2.41308530218450 13.22249448223564  F 2.45520810178635 1.14046686653131 12.16066290168487  F -0.60794294214908 -2.35674777788976 11.13902983360377  F 2.33612572667731 0.32798176711556 10.16753951366620  N 0.00970724128363 3.31299678551569 11.79817226744971  N -2.86190266733412 0.00940589624846 12.09596120110298  F 1.44093166011041 -2.49279322040343 10.44748676826618  N -1.14999619209129 1.62511422097197 14.12351947096020  N -1.70419402497668 1.68396135809976 9.77101311472966  C 0.69446822535043 4.22606531698037 11.73860656700293  C -3.55045092698551 -0.89994006796622 12.16765086236418  C 0.84906777288701 -0.58056275415823 11.79680099603999  C -1.91093179805836 1.75803183654354 8.64943703920565  C -0.93867368152759 1.52875559593723 15.24256561890595  C 0.87136977756752 -1.24639777171971 13.21357160385823  C 2.25949585291913 0.01386361163663 11.46588189171344  C -2.17900506279460 1.86498158853645 7.22333348826365  H -1.38394323519253 2.43981561325669 6.74664480064910  H -3.13433838721997 2.37114586270331 7.07643812357207  H -2.21997458369412 0.86755778822828 6.78370638187322  Ti -1.42453446260147 1.66075395902826 11.94683869348112  C -0.66205458800337 1.39513206994472 16.66475636355557  H -0.38771030843965 2.36751505567883 17.07583742546336  H 0.16141158829740 0.69274170942824 16.80274849508990  H -1.55098807712947 1.02292242447105 17.17560926848515  C 0.45153788380256 -1.64469404755416 10.71888644657827  C 1.55602707711858 5.39632304174820 11.66514832586910  H 2.49893148090465 5.18679379897070 12.17196460640685  H 1.05729542342363 6.23799592992509 12.14813716501234  H 1.75101068710315 5.63964144541177 10.61992067318723  O -2.79084337273711 2.90505985369287 12.10864277881702  C -3.68515805958711 3.91423308194184 12.10510023198781  C -3.25022608555904 4.98861007602563 13.15806224447179  F -2.86689643628054 4.39493237317209 14.29146964936882  F -4.22468207840748 5.84968276641956 13.44242438006251  F -2.19519153533754 5.68586366313890 12.70207488531757  C -3.73179888641330 4.55903711287071 10.67929102399073  F -4.34995416105698 3.73891789163430 9.81548569140600  F -2.48919426377685 4.74809045120527 10.21489719617651  F -4.36195559577028 5.73150174468958 10.66656964381261  C -5.09456179739533 3.34219819705550 12.47766768212360  F -5.32044054657967 2.20831072950136 11.80457428319955  F -6.08024984650610 4.19519388798353 12.20001567826729  F -5.14427366673323 3.04810021737150 13.78206564012514 | | O -0.08926117106233 0.41445093586155 11.82439901223601  C -4.63533389929848 -1.95921576212355 11.91179725054896  H -5.53153573985738 -1.65813392282247 11.36790134323676  H -4.90704115330188 -2.22859155418494 12.93319037715134  H -4.18350733637316 -2.82095131326221 11.41868725256195  N 0.10046804899552 3.29926138373486 11.95854652212057  N -2.93287784451999 0.00704306219747 11.94686130599255  N -1.28573701713373 1.52991786724158 14.18357599841877  N -1.55314651826671 1.78183667832072 9.72307298093389  C 0.84976251319882 4.16305965618762 11.96950656338673  C -3.68255121609058 -0.85638623515406 11.93313671945009  C 0.92297451088612 -0.55334630682641 11.75806156826963  C -1.66952175897802 1.89062275816555 8.59068446113576  C -1.19588266367860 1.43877322575944 15.31992188136471  C 1.47573636991885 -0.77578786580702 13.16746743188525  C 2.02363663194924 -0.04110355476695 10.82740225781182  C -1.82389654178157 2.03460578552504 7.14858829220214  H -0.95981561230014 2.55844037195695 6.73799012968376  H -2.72851202566527 2.60596994516327 6.93592798543174  H -1.90027153207352 1.04905339262315 6.68764000851508  Ti -1.41643669178395 1.65318046758894 11.95312392750551  C -1.07736508332086 1.31585107018586 16.76737488849415  H -0.77212045038032 2.27264571107846 17.19282805750762  H -0.33050011390860 0.55791127587100 17.00751678334240  H -2.03954425687472 1.02422613515836 17.19024860057589  C 0.32117619494301 -1.84939294439391 11.21131472343046  C 1.80225516095464 5.26620435541903 11.98685199099808  H 2.69518897802872 4.97011359276131 12.53880633849399  H 1.34738215492726 6.13223338937616 12.46953192528668  H 2.08014116451496 5.52646489018958 10.96475981920547  O -2.74374388255609 2.89310175850079 12.08125623235490  C -3.74986316902784 3.86728148178745 12.14242027888925  C -3.14202585008159 5.16082857048826 12.68849590865352  C -4.29704045785906 4.09064686647177 10.73086601965455  C -4.85682482978807 3.36387860263225 13.07044660271423  H -2.34027620618159 5.50442149572118 12.02941528032084  H -2.72264879027368 4.98632455222954 13.68275126616127  H -3.89314274974867 5.95163608407562 12.76176522346256  H -4.70154255979937 3.15561961856661 10.33418912171316  H -3.49608393194234 4.43158958557675 10.06928487721017  H -5.09145071476533 4.84153797767289 10.72843920339015  H -5.26950955225310 2.42728322229881 12.68663509256336  H -5.66760049880831 4.09243785403620 13.15339022722105  H -4.45222447576098 3.17897576611036 14.06885214097040  H 2.27438381126430 -1.52217723091236 13.16618506488171  H 0.67875635978288 -1.12225873588835 13.83093752365730  H 1.87598948870801 0.16079239132514 13.56467403696495  H -0.10179926293562 -1.67561889571543 10.21845097567635  H -0.47676221671619 -2.19879387276949 11.87197128265824  H 1.07670487207180 -2.63566935924760 11.13475128275538  H 1.61443550496874 0.14393612707367 9.83091295878450  H 2.83827436139825 -0.76480680748634 10.73984726696986  H 2.43235303853657 0.89683957442780 11.21216960219398 |
